# Burden of gastroesophageal reflux disease in 204 countries and territories, 1990–2019: a systematic analysis for the Global Burden of disease study 2019

**DOI:** 10.1186/s12889-023-15272-z

**Published:** 2023-03-29

**Authors:** Na Li, Wan-Li Yang, Mei-Hong Cai, Xiang Chen, Ran Zhao, Meng-Ting Li, Xia-Lin Yan, Li-Wei Xue, Liu Hong, Ming-Yu Tang

**Affiliations:** 1 Division of Gastroenterology and Hepatology, NHC Key Laboratory of Digestive Disease, Ministry of Health, Renji Hospital, School of Medicine, Shanghai Jiao Tong University, Shanghai Institute of Digestive Disease, Shanghai, 200125 China; 2 State Key Laboratory of Cancer Biology and National Clinical Research Center for Digestive Diseases, Xijing Hospital of Digestive Diseases, Fourth Military Medical University, Xi’an, 710032 Shaanxi Province China; 3 Department of Gastroenterology, Xinhua Hospital Affiliated to Shanghai Jiao Tong University School of Medicine, Shanghai, 200092 China; 4 Division of Pulmonary Medicine, Key Laboratory of Heart and Lung, the First Affiliated Hospital of Wenzhou Medical University, Wenzhou, 325000 Zhejiang Province China; 5 Department of Gastroenterology, Guangdong Provincial People’s Hospital, Guangdong Academy of Medical Sciences, Guangzhou, 510080 China; 6 Department of Gastroenterology, The Affiliated People’s Hospital of Ningbo University, Zhejiang, China; 7 Department of Gastrointestinal Surgery, School of Medicine, Shanghai Tenth People’s Hospital, Tongji University, Shanghai, China; 8 Department of Gastroenterology, Ruian People’s Hospital, Zhejiang Province, Wenzhou, 325200 China

**Keywords:** Gastro-esophageal reflux disease, Global burden disease, Age-standardized rate, Epidemiology

## Abstract

**Introduction:**

For effective preventive strategies against GORD (gastro-esophageal reflux disease), we assessed the GORD burden from 1990 to 2019.

**Methods:**

The burden of GORD between 1990 and 2019 was evaluated globally, regionally, and nationally. Using ASIR (age-standardized incidence), ASYLDs (age-standardized years lived with disabilitys), we compared them to the GBD world population per 100,000. The estimates were based on 95% uncertainty intervals (UIs). The AAPC (average annual percent change) in incidence, YLDs, along with prevalence rates with associated 95% CIs were estimated.

**Results:**

Data to estimate the burden of GORD are scarce till now. The global ASIR of GORD in 2019 was 3792.79 per 100,000, an increase AAPC of 0.112% from 1990. The prevalence of GORD increased with a AAPC of 0.096% to 9574.45 per 100,000. Global ASYLDs in 2019 was 73.63, an increase AAPC of 0.105% from 1990. The GORD burden varies greatly depending on the development level and geographical location. USA demonstrated the most obvious decreasing trend in burden of GORD, while Sweden had an increasing trend. That the increase in GORD YLDs was mediated primarily by the growth and aging of population, was revealed by decomposition analyses. There was an inverse relationship between SDI (socio-demographic index) and GORD-burden. Frontier analyses revealed significant scope of improvement in the status of development at all levels.

**Conclusion:**

GORD is a public health challenge, especially in Latin America. Some SDI quintiles had declining rates, while some countries experienced increased rates. Thus, resources should be allocated for preventative measures based on country-specific estimates.

**Supplementary Information:**

The online version contains supplementary material available at 10.1186/s12889-023-15272-z.

## Introduction

Gastro-esophageal reflux disease (GORD) is a chronic, commonly occurring, persistent disease affecting the upper tract of the digestive system [1]. It affects up to 20% of populations in Western countries and is increasing in prevalence worldwide [2]. GORD is defined as recurring symptoms or mucosal damage that develops when the distal esophagus is exposed to the gastric content reflux [1, 3]. Typical symptoms of GORD include but are not limited to regurgitation, heartburn, and chest pain [4]. Some extra-esophageal symptoms can also present with GORD, such as laryngitis, asthma, dental erosions, and chronic cough [4, 5]. In addition, GORD may be an important risk factor for many diseases, including esophageal squamous epithelium inflammation [6], cancer [7], mental disorders [8], head and neck diseases [9], respiratory disease [10], and cardiovascular diseases [11].

Although several systematic reviews and cross-sectional surveys have been published that describe the evaluation of prevalence and incidence of GORD globally and in specific regions or countries, data are limited; besides, interpretation of symptoms has been a challenge due to cultural and language differences [12]. For example, a meta-analysis performed by Eusebi et al. reported the prevalence of gastric reflux symptoms instead of GORD prevalence; however, this study presented the prevalence of disease and risk factors based on pooled estimates globally and regionally [4]. Besides, they did not account for non-standard studies. The prevalence of GORD at a global level was assessed by El-Serag et al. in their systemic review, but they included only 28 studies reported until 2011; in the interim, many surveys have been published [13]. Moreover, they did not include studies from Africa and those not in English. A recent meta-analysis performed by Nirwan and colleagues comprehensively analyzed and investigated GORD distribution and the effect of risk factors on the GORD prevalence in all UN geoscheme regions. But significant heterogeneity was still present between studies [14]. As a result of these systematic reviews didn’t include enough studies across geography and time, the authors could not systematically evaluate the incidence and burden of GORD symptoms. The GBD 2017 gastroesophageal reflux disease collaborators gave a systematic analysis of GORD burden worldwide [15]. Nevertheless, the burden of GORD varies between and within regions and over time periods.

These findings highlight again how little we still know about the GORD and indicate that GORD threatens human health and further aggravates the burden of patients and society. The last three decades witnessed accelerated global population growth, aging, and epidemiologic transition [16]. It is crucial to assess the current global burden of GORD and evaluate the trend over time to prepare strategies for prevention that are more effective. With the Global Burden of Diseases, Injuries, and Risk Factors Study data 2019, this study evaluated the burden of GORD in terms of incidence, prevalence, and years lived with disability (YLDs) for GORD at the global, SDI quintile, regional, and national levels. We then investigated the demographic and epidemiologic factors that mediated the changes in GORD burden over the past three decades. The relationship between the burden of GORD and any country’s economic prosperity was also analyzed.

## Methods

### Overview

GBD 2019 is the latest data that reports the trends and levels of the epidemiology of different global injuries and diseases. The methods of GBD 2019 have been detailed in earlier reports [17, 18]

The detailed information on incidence, prevalence, and YLDs can be obtained from the IHME website (http://ghdx.healthdata.org/gbd-results-tool) [19]. Each estimate was presented as counts and age-standardized rates/ 100,000 individuals with 95% UIs (95% uncertainty intervals). The screening rules used were: the cause was “gastro-esophageal reflux disease,” and the location name was “global,” and as measures, “incidence,” “prevalence,” and “YLDs” were chosen as the measures.

### Statistical analysis

The age-standardized rate estimates and counts per 100,000 individuals were presented applying the GBD structure for the standard population. Apropos to the GBD framework for all estimates, 95% UIs were provided [19]. The final estimated were calculated using the mean estimate of 1000 draws, with the lower and upper 95% UI bounds being the 2.5th and 97.5th ranked values, respectively, throughout the 1000 draws. To calculate the AAPC (average annual percentage change) in GORD case incidences from 1990 to 2019, the Joinpoint Trend Analysis Software (v 4.7.0.0) was used. For a better insight into aspects that mediated variation in GORD YLDs within 1990 and 2019, decomposition analyses were conducted by age structure, population size, and changes in epidemiology [16]. To assess the association of sociodemographic development and GORD burden, using the SDI as a method for estimating the least achievable rate of YLDs, a frontier analysis was performed to determine the least achievable rate [16]. The detailed description of the decomposition assessment and frontier analysis was described in the supplementary method.

## Results

### Incidence rate of GORD (during 1990—2019 data)

**Fig. 1:**
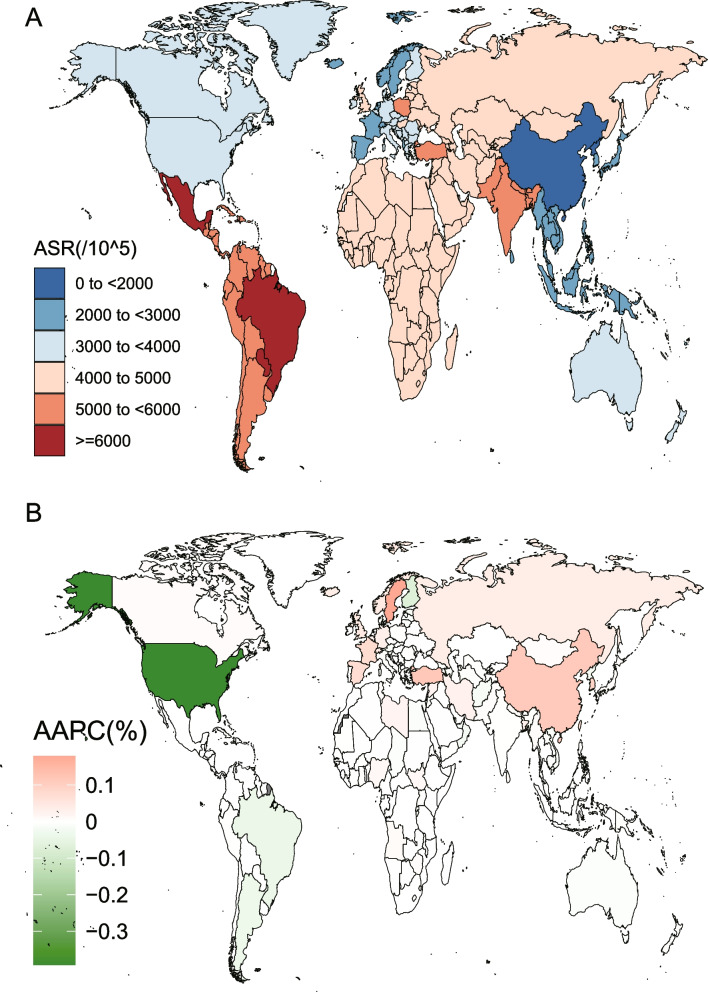
The incidence of GORD for both sexes in 204 countries and territories. **A** The ASIR of GORD in 2019; (**B**) The AAPC of ASIR of GORD from 1990 to 2019. GORD, Gastro-oesophageal reflux disease; ASIR, age-standardized incidence rate; AAPC, average annual percentage change

Table 1 and Table S1 present the age-standardized estimates of GORD incidence for all locations in GBD 2019. In 2019, the global mean age-standardized estimates of GORD incidence for all locations was 3793 for a population per 100,000 (Table 1). For any region, the range of the estimate was 6145 cases per population of 100,000 in Tropical Latin America to 1847 per population of 100,000 in East Asia (Table 1). The age-standardized incidence rate (ASIR) of GORD decreased with increasing SDI values in 2019 (Table 1). At the national or territory strata, the calculated mean of age-standardized GORD incidence for all locations in 2019 was 6147 (95% UI 5457.64 to 6790.57) cases per population of 100,000 in Brazil to1842 (95% UI 1607.09 to 2133.51) per population of 100,000 in China (Table S1). China (1841.66 (95% UI 1607.09 to 2133.51)) and North Korea (1931.72 (95%UI 1691.95 to 2244.82)) exhibited the lowest estimates ASIR in 2019. The highest estimates were for Brazil (6146.6 (95% UI 5457.64 to 6790.57)) and Paraguay (6069.06 (95%UI 5387.11 to 6728.66)). Figure 1A shows the variation in the ASIR based on the geographical location of GORD in 2019. When standardizing GORD for age, it experiences the highest incidence in Latin America (Andean, Central, Tropical, and Southern), South Asia, and at the Caribbean, at greater than 5000 cases per population of 100,000. ASIR of GORD was lowest in Asia Pacific with High-income, Southeast Asia, East Asia, and Oceania, at less than 3000 cases per population of 100, 000. Country/territory-specific data showed that Brazil, Mexico, and Paraguay experienced higher ASIR for GORD (> 6000 per 100,000). The territories/countries with the lowest GORD ASIR were North Korea and China (< 2000 per 100,000) (Fig. 1A; Table S1).

**Table 1:** Incidence of gastro-oesophageal reflux disease in 1990 and 2019 for both sexes and all locations, with AAPC from 1990 and 2019

location	1990		2019		AAPC % (95% CI) 1990–2019
	Cases (95% UI)	Age-standardised incidence per 100 000 population (95% UI)	Cases (95% UI)	Age-standardised incidence per 100 000 population (95% UI)	
Global	177,004,114 (154,833,551 to 201,151,187)	3687.27 (3256.92 to 4165.86)	309,381,599 (272,527,195 to 349,510,437)	3792.79 (3341.66 to 4280.02)	0.112 (0.0887 to 0.1354)
**Sex**					
Female	92,635,001 (81,195,363 to 104,991,220)	3825.6 (3378.2 to 4311.11)	162,117,782 (143,288,942 to 182,238,845)	3929.78 (3465.46 to 4430.41)	0.1004 (0.0763 to 0.1245)
Male	84,369,113 (73,743,127 to 96,136,377)	3548.43 (3129.86 to 4016.03)	147,263,817 (129,482,109 to 166,975,925)	3655 (3218.54 to 4142.05)	0.1168 (0.0829 to 0.1506)
**SDI**					
High SDI	31,618,586 (27,664,986 to 35,989,331)	3388.61 (2968.37 to 3866.89)	42,820,565 (37,890,499 to 49,042,946)	3319.5 (2906.37 to 3810.77)	-0.0688 (-0.0985 to -0.0391)
High-middle SDI	40,934,868 (35,807,097 to 46,496,531)	3524.13 (3116.29 to 3994.07)	59,657,101 (52,761,656 to 67,694,442)	3410.01 (3011.96 to 3854.01)	-0.0929 (-0.1344 to -0.0514)
Middle SDI	47,288,734 (41,153,433 to 53,994,580)	3203.91 (2827.63 to 3614.98)	90,256,897 (79,322,383 to 102,221,182)	3461.2 (3052.34 to 3901.86)	0.3001 (0.2665 to 0.3337)
Low-middle SDI	39,928,729 (34,917,892 to 45,373,810)	4603.9 (4087.96 to 5177.94)	77,640,490 (68,485,515 to 87,712,250)	4650.41 (4124.9 to 5226.35)	0.0448 (0.026 to 0.0637)
Low SDI	17,127,223 (14,930,930 to 19,502,159)	4758.96 (4219.43 to 5379.93)	38,817,852 (33,856,901 to 44,260,966)	4741.79 (4202.29 to 5361.43)	-0.0128 (-0.0167 to -0.0089)
**Region**					
High-income Asia Pacific	4,987,761 (4,331,624 to 5,774,219)	2532.38 (2208.26 to 2926)	6,773,592 (5,959,335 to 7,831,094)	2572.68 (2244.2 to 2974.08)	0.088 (0.0268 to 0.1491)
High-income North America	12,903,826 (11,185,018 to 14,660,689)	4095.69 (3560.93 to 4654.85)	16,745,460 (14,814,489 to 19,114,605)	3727.97 (3253.14 to 4285.69)	-0.362 (-0.437 to -0.2869)
Western Europe	15,182,432 (13,383,136 to 17,345,731)	3304.06 (2909.15 to 3774.92)	18,779,722 (16,645,762 to 21,466,481)	3303.3 (2898.46 to 3775.1)	-0.0026 (-0.0242 to 0.019)
Australasia	780,400 (683,949 to 898,525)	3516.72 (3073.84 to 4048.01)	1,243,583 (1,099,620 to 1,422,203)	3511.78 (3070.46 to 4043.23)	-0.0101 (-0.1179 to 0.0978)
Andean Latin America	1,807,399 (1,582,992 to 2,032,179)	5976.14 (5315.06 to 6644.79)	3,753,606 (3,318,993 to 4,182,895)	5974.25 (5312.88 to 6641.29)	-0.0013 (-0.0015 to -0.0011)
Tropical Latin America	8,115,960 (7,151,426 to 9,051,242)	6164.75 (5485.23 to 6806.08)	15,160,898 (13,456,970 to 16,783,418)	6144.66 (5455.45 to 6787.85)	-0.0394 (-0.082 to 0.0032)
Central Latin America	7,837,685 (6,870,971 to 8,793,414)	6039.93 (5379.89 to 6694.89)	15,486,351 (13,756,037 to 17,199,541)	6040.22 (5378.11 to 6691.82)	0.0002 (-0.0001 to 0.0004)
Southern Latin America	2,394,344 (2,105,407 to 2,723,237)	5003.17 (4406.68 to 5687.32)	3,710,048 (3,275,988 to 4,214,316)	5002.39 (4405.74 to 5686.77)	-0.0241 (-0.0647 to 0.0166)
Caribbean	1,904,049 (1,678,441 to 2,128,661)	5976.8 (5315.24 to 6644.09)	2,986,873 (2,664,282 to 3,321,929)	5975.49 (5313.4 to 6642.33)	-0.0007 (-0.0008 to -0.0006)
Central Europe	5,781,318 (5,071,870 to 6,555,032)	4262.46 (3756.68 to 4830.14)	6,438,335 (5,709,327 to 7,286,614)	4301.11 (3789.35 to 4872.82)	0.0318 (0.031 to 0.0326)
Eastern Europe	11,431,851 (10,080,437 to 13,020,759)	4487.1 (3948.05 to 5093)	11,935,372 (10,564,743 to 13,560,206)	4478.85 (3938.02 to 5080.2)	0.0189 (-0.0697 to 0.1076)
Central Asia	2,420,746 (2,108,738 to 2,753,255)	4171.74 (3663.06 to 4766.35)	3,824,162 (3,325,278 to 4,381,321)	4168.45 (3658.11 to 4764.72)	-0.0026 (-0.0028 to -0.0025)
North Africa and Middle East	12,468,509 (10,783,617 to 14,236,294)	4781.05 (4214.49 to 5396.43)	28,325,327 (24,825,600 to 32,106,727)	4799.43 (4259.75 to 5391.27)	0.0206 (0.0011 to 0.0402)
South Asia	44,134,149 (38,514,110 to 50,110,470)	5222.37 (4632.67 to 5883.96)	90,165,761 (79,572,298 to 102,133,256)	5223.34 (4636.37 to 5882.55)	-0.0009 (-0.0168 to 0.015)
Southeast Asia	8,369,178 (7,205,877 to 9,699,036)	2213.29 (1932.35 to 2562.26)	15,456,438 (13,366,180 to 17,875,431)	2211.43 (1931.03 to 2560.16)	-0.0029 (-0.0031 to -0.0026)
East Asia	21,642,039 (18,659,707 to 25,044,722)	1853.82 (1617.2 to 2149.28)	33,939,051 (29,518,619 to 39,643,842)	1847.31 (1612.24 to 2139.6)	0.1076 (-0.0663 to 0.2817)
Oceania	105,654 (90,640 to 122,176)	2164.68 (1891.94 to 2493.36)	237,822 (204,406 to 275,720)	2165.05 (1892.06 to 2495.02)	0.0004 (0.0003 to 0.0006)
Western Sub-Saharan Africa	5,861,254 (5,103,901 to 6,730,922)	4465.94 (3929.26 to 5079.05)	14,237,389 (12,381,401 to 16,433,018)	4469.45 (3926.83 to 5086.96)	0.0028 (0.0026 to 0.003)
Eastern Sub-Saharan Africa	5,444,074 (4,729,652 to 6,280,442)	4451.93 (3906.53 to 5069.11)	12,795,708 (11,077,327 to 14,751,190)	4454.59 (3909.59 to 5068.92)	0.0021 (0.0019 to 0.0023)
Central Sub-Saharan Africa	1,615,620 (1,403,484 to 1,865,969)	4388.2 (3855.17 to 5009.03)	4,054,442 (3,514,547 to 4,687,251)	4388.34 (3853.19 to 5007.99)	0.0001 (0 to 0.0002)
Southern Sub-Saharan Africa	1,815,866 (1,581,964 to 2,082,343)	4522.2 (3978.59 to 5137.46)	3,331,659 (2,900,599 to 3,812,232)	4524.95 (3983 to 5139.59)	0.0024 (0.002 to 0.0029)

*UI* uncertainty interval, *CI* confidence interval, *AAPC* average annual percent change

As shown in Figure S1, increase in incidence counts over time was observed across all GBD regions. The global estimates of the ASIR of GORD increased over time, at 3687 (95%UI 3257 to 4166) in 1990 and 3793 (95%UI 3342 to 4280) cases in 2019 per population of 100,000, with an AAPC of 0.112% (95%CI 0.088% to 0.135%). Between 1990 and 2019, a larger increase was observed in the world count of prevalent cases-from 177 million (155 to 201; 1990) to 309 million (273 to 350; 2019) (Table 1). At the regional level, the ASIR of GORD was stable over time in Australasia, Tropical Latin America, Latin America (Southern and Central), Western Europe, Eastern Europe, Asia (South and East), with the AAPC exhibiting a confidence interval including zero (Table 1). However, the estimates decreased by 0.069% (95% CI to 0.039% to 0.099%), 0.093% (95% CI 0.051% to 0.134%), 0.013% (95% CI 0.009% to 0.017%), and 0.362% (95%CI 0.287% to 0.437%) across countries with High SDI, High-middle SDI, Low SDI, and High-income North America, respectively. In comparison, the increasing trend was high in countries with Middle SDI, Low-middle SDI, High-income Asia Pacific, and Central Europe (Table 1). From 1990 to 2019, the AAPC in the estimates for ASIR differed significantly among countries. Sweden (0.178% (95% CI 0.150% to 0.205%)) and Turkey (0.115% (95%CI 0.090% to 0.140%)) showed the largest increases. United States of America (USA) (-0.391% (95% CI -0.472% to -0.311%)) and Finland (-0.069% (95% CI -0.128% to -0.010%)) showed significant decreasing trends (Table S1). Geographical variation in the AAPC of ASIR of GORD from 1990 to 2019 is shown in Fig. 1B. From 1990 until 2019, a worse trend of the change for GORD incidence rate presents (AAPC > 0%) in High-income Asia Pacific, North Africa and the Middle East, Central Europe, East Asia, Oceania, and Sub-Saharan Africa. However, the GORD incidence trend improved (AAPC < 0%) in Andean Latin America, High-income North America, Caribbean, Central Asia, and Southeast Asia.

### Prevalence rate of GORD throughout 1990 to 2019

Worldwide ASPR (age-standardized prevalence rate) of GORD in 1990 and 2019 is presented in Table S2 and Table S3. Globally, prevalence cases of GORD increased largely from 441.6 million to 783.9 million in 1990 to 2019, respectively, but ASPR increased slightly from 9344.52 persons per 100,000 in 1990 to 9574.45 persons per 100,000 in 2019. Considering SDI quintiles, High and High-middle, and Low SDI quintiles experienced a decline in prevalence, but increasing in the other two SDI quintiles saw increases from 1990 to 2019. Alarmingly, Middle SDI quintiles had the most considerable increase with AAPC at 0.296% (95% CI 0.261% to 0.332%). As for geographical regions, the ASPR of GORD per 100,000 was the highest in Tropical Latin America (16,207.5), followed by Central Latin America (15,949.53), Caribbean (15,935.32), and Andean Latin America (15,932.06) in 2019 (Table S2, Figure S2A). From 1990 to 2019, the ASPR trend was stably decreasing in majority of geographical regions except for High-income Asia Pacific (Table S2). On examining territories and countries, in 2019, China, the USA, and India had the highest cases of incidence at 81.6 million, 39.7 million, and 181.6 million, respectively (Table S3). Brazil and Paraguay had the highest ASPR at 16,204.14/100,000 and 16,310.51/100,000 persons in 2019, respectively (Table S3). Sweden (0.216% (95% CI 0.183% to 0.249%)) and USA (-0.497% (95% CI -0.600% to -0.393%)), ASPR increases and declines are the most prevalent in these countries (Table S3, Figure S2B). Geographical variation in the AAPC of ASIR of GORD from 1990 to 2019 is shown in Fig. 1B. From 1990 until 2019, a worse trend of the change for GORD incidence rate presents (AAPC > 0%) in High-income Asia Pacific, North Africa and the Middle East, Central Europe, East Asia, Oceania, and Sub-Saharan Africa. However, the GORD incidence trend improved (AAPC < 0%) in Andean Latin America, High-income North America, Caribbean, Central Asia, and Southeast Asia.

### The YLDs (years lived with disability) of GORD (1990 to 2019 data)

ASYLDs also increased globally over time, from 71.68 (95% UI 36.95 to 128.64) per population of 100,000 in 1990 to 73.63 (95% UI 38.03 to 132.08) per population of 100,000 in 2019, an increase AAPC of 0.105% (95%CI 0.078% to 0.131%) (Table 2). ASYLDs did not exhibit significant change between 1990 and 2019 in several regions for GBD estimation, including countries with Low SDI, Western Europe, Tropical and Latin America, North Africa, Eastern Europe, and the Middle East, as well as South and East Asia. From 1990 to 2019, a worsening trend in the pattern of change in ASYLDs for GORD (AAPC > 0%) was observed in Middle SDI and Low-middle SDI countries, Andean Latin America, High-income Asia Pacific, Central Europe, Central Latin America, Southeast Asia, Western, Eastern and Central Sub-Saharan Africa (Figure S3B; Table S4). High-income North America (-0.471% (95% CI -0.564% to -0.377%)), High SDI countries (-0.0962% (95% CI -0.134% to -0.058%)), and High-middle SDI countries (-0.087% (95% CI -0.133% to -0.042%)) were the top three regions with improving trends in ASYLDs rates. Similar trends were observed for ASYLDs by country/territory. A worsening trend (AAPC > 0%) in ASYLDs for GORD was seen in Turkey (0.202% (95% CI 0.153% to 0.250%)) and Sweden (0.211% (95% CI 0.176% to 0.247%)) (Figure S3B), while USA (-0.506% (95% CI -0.606% to -0.405%)) and Finland (-0.057% (95% CI -0.114% to -0.011%)) show an improving trend (AAPC < 0%) in ASYLDs of GORD. According to geographical regions, variation in counts of YLDs suggests variation in incidence and prevalence, and Figure S1 showed a consistent increase in prevalence, incidence, and total YLDs with time throughout all GBD regions. The regions with the highest estimate of the incidence and prevalence rate of GORD in 2019 also exhibited the highest YLDs (Figure S1).

**Table 2:** YLDs of gastro-oesophageal reflux disease in 1990 and 2019 for both sexes and all locations, with AAPC from 1990 and 2019

location	1990		2019		AAPC % (95% CI) 1990–2019
	Cases (95% UI)	Age-standardised YLDs per 100 000 population (95% UI)	Cases (95% UI)	Age-standardised YLDs per 100 000 population (95% UI)	
Global	3,402,303 (1,762,371 to 6,093,058)	71.68 (36.95 to 128.64)	6,028,428 (3,103,988 to 10,815,634)	73.63 (38.03 to 132.08)	0.1046 (0.0784 to 0.1309)
**Sex**					
Female	1,779,408 (922,964 to 3,181,335)	74.25 (38.28 to 132.56)	3,155,918 (1,623,853 to 5,632,673)	76.13 (39.35 to 135.76)	0.0964 (0.0693 to 0.1235)
Male	1,622,895 (839,407 to 2,902,319)	69.08 (35.62 to 124.33)	2,872,510 (1,479,428 to 5,183,408)	71.11 (36.71 to 127.76)	0.1149 (0.0803 to 0.1495)
**SDI**					
High SDI	613,882 (313,803 to 1,109,140)	65.37 (33.27 to 118.08)	829,714 (420,693 to 1,492,911)	63.4 (32.17 to 114.26)	-0.0962 (-0.1343 to -0.0581)
High-middle SDI	802,970 (414,860 to 1,445,578)	69.26 (35.58 to 124.07)	1,193,576 (610,303 to 2,157,747)	67.09 (34.59 to 120.4)	-0.0874 (-0.1327 to -0.0422)
Middle SDI	900,248 (468,334 to 1,613,342)	62.16 (32.01 to 111.64)	1,763,594 (907,809 to 3,164,464)	67.19 (34.68 to 120.62)	0.3016 (0.2664 to 0.3368)
Low-middle SDI	760,700 (398,400 to 1,354,386)	89.83 (46.6 to 161.42)	1,504,588 (784,025 to 2,678,872)	90.99 (47.14 to 163.13)	0.0593 (0.0325 to 0.0861)
Low SDI	322,418 (169,182 to 571,645)	92.18 (47.98 to 165.24)	733,185 (384,712 to 1,299,601)	92.25 (48.02 to 165.73)	0.0009 (-0.0052 to 0.007)
**Region**					
High-income Asia Pacific	93,511 (47,357 to 169,565)	47.29 (24.05 to 85.78)	129,322 (65,755 to 234,499)	48.25 (24.6 to 87.46)	0.1117 (0.0353 to 0.1882)
High-income North America	257,045 (132,009 to 459,535)	81.07 (41.69 to 145.33)	325,592 (164,304 to 579,722)	71.62 (36.26 to 128.42)	-0.4706 (-0.5642 to -0.3768)
Western Europe	293,589 (149,481 to 534,277)	63.26 (32.24 to 113.81)	366,507 (186,145 to 663,160)	63.3 (32.3 to 114.14)	0.0094 (-0.0358 to 0.0547)
Australasia	14,851 (7585 to 26,922)	66.71 (34.03 to 120.31)	23,867 (12,122 to 43,405)	66.66 (34.06 to 121.04)	-0.0076 (-0.1184 to 0.1034)
Andean Latin America	36,034 (18,786 to 63,657)	123.03 (63.86 to 217.88)	76,946 (39,952 to 136,341)	123.15 (64 to 218.31)	0.0036 (0.0027 to 0.0045)
Tropical Latin America	161,031 (84,450 to 287,389)	125.29 (65.3 to 221.8)	309,932 (160,236 to 553,965)	124.78 (64.6 to 222.07)	-0.0453 (-0.1004 to 0.0098)
Central Latin America	154,007 (80,562 to 272,619)	122.69 (63.8 to 218.43)	314,343 (163,301 to 558,984)	122.92 (63.83 to 218.74)	0.0053 (0.0034 to 0.0073)
Southern Latin America	50,135 (25,797 to 90,201)	105.21 (54.14 to 189.23)	78,553 (40,647 to 140,874)	105.12 (54.12 to 187.46)	-0.0382 (-0.1035 to 0.0271)
Caribbean	38,417 (19,999 to 68,157)	122.99 (64.07 to 217.41)	61,606 (32,186 to 109,087)	122.78 (64.04 to 216.84)	-0.006 (-0.0071 to -0.0049)
Central Europe	113,974 (57,965 to 204,307)	83.43 (42.78 to 148.19)	129,192 (65,727 to 230,366)	84.64 (43.44 to 150.2)	0.0501 (0.0475 to 0.0526)
Eastern Europe	223,903 (113,783 to 406,443)	86.98 (44.47 to 157.52)	236,818 (120,535 to 428,492)	87.11 (44.45 to 157.69)	0.037 (-0.0739 to 0.148)
Central Asia	46,360 (23,783 to 83,401)	80.89 (41.35 to 146.25)	74,174 (38,068 to 133,702)	80.81 (41.3 to 145.7)	-0.0032 (-0.0042 to -0.0022)
North Africa and Middle East	239,878 (124,957 to 431,913)	94.3 (48.44 to 170.89)	559,710 (289,618 to 1,006,943)	95.07 (48.91 to 170.82)	0.0157 (-0.0148 to 0.0461)
South Asia	840,433 (438,714 to 1,494,105)	101.66 (52.54 to 183.28)	1,744,755 (909,045 to 3,124,842)	102.04 (52.78 to 183.85)	0.0132 (-0.0117 to 0.038)
Southeast Asia	155,379 (79,473 to 278,926)	41.77 (21.23 to 75.63)	293,183 (148,900 to 531,697)	41.85 (21.26 to 76.1)	0.007 (0.0059 to 0.0081)
East Asia	406,712 (207,739 to 731,861)	35.12 (17.82 to 63.31)	654,695 (329,593 to 1,192,670)	35.05 (17.78 to 63.23)	0.1215 (-0.0698 to 0.3132)
Oceania	1940 (993 to 3523)	40.54 (20.7 to 73.93)	4403 (2243 to 7993)	40.48 (20.57 to 73.75)	-0.0054 (-0.0088 to -0.0019)
Western Sub-Saharan Africa	109,885 (57,346 to 194,256)	86.14 (44.53 to 154.02)	266,797 (139,635 to 471,353)	86.45 (44.83 to 154.98)	0.0125 (0.012 to 0.013)
Eastern Sub-Saharan Africa	101,115 (52,718 to 178,171)	85.62 (44.37 to 152.89)	238,775 (124,573 to 420,008)	86.02 (44.6 to 154.04)	0.0169 (0.0156 to 0.0182)
Central Sub-Saharan Africa	30,039 (15,570 to 53,247)	84.13 (43.69 to 149.18)	75,814 (39,443 to 133,500)	84.58 (43.95 to 151.08)	0.018 (0.0158 to 0.0203)
Southern Sub-Saharan Africa	34,064 (17,852 to 60,250)	87.11 (45.02 to 155.84)	63,445 (32,819 to 113,194)	86.82 (44.8 to 155.01)	-0.0135 (-0.0169 to -0.0102)

*UI* uncertainty interval, *CI* confidence interval, *AAPC* average annual percent change, *YLDs* Years Lived with Disability

### The YLDs (years lived with disability) of GORD (1990 to 2019 data)

Age-standardized YLDs (ASYLDs) due to GORD for each location calculated in GBD 2019 are mentioned in Table 2 and Table S4. Globally, GORD incurred 3.40 million (95% UI 1.76–6.09) ASYLDs in 1990. In 2019, it had raised to 6.03 million (95% UI 3.10–10.82), an increase AAPC of 0.105% (95%CI 0.078% to 0.131%) (Table 2). Regionally, the mean ASYLDs of GORD per 100,000 in 2019 was the highest in Tropical Latin America (124.78), followed by Andean Latin America (123.15), Central Latin (122.92), and Caribbean (122.78); each of these were greater than the global ASYLDs of GORD (73.63) (Table 2). However, East Asia (35.05 (95% UI 17.78 to 63.23)), Southeast Asia (41.85 (95% UI 21.26 to 76.1)), and Oceania (40.48 (95% UI 20.57 to 73.75)) had the lowest rates of ASYLDs (Table 2). The ASYLDs of GORD increased with decreasing values of SDI in 2019 (Table 2). Geographical variation in ASYLDs was shown in Figure S3A. Country/territory-specific data showed that Paraguay, Brazil, Bermuda, Bahamas, Colombia, Barbados, Dominican Republic, Costa Rica, Ecuador, United States Virgin, Peru, and Venezuela (Bolivarian Republic) experienced higher ASYLDs of GORD per 100,000 (> 123 per 100, 000). The nations/territories with the lowest ASYLDs of GORD per 100, 000 were China, North Korea, Norway, and Switzerland (< 40 per 100, 000) (Figure S3A; Table S4).

Figure S1 showed a consistent increase in prevalence, incidence, and total YLDs with time throughout all GBD regions. The regions with the highest estimate of the incidence and prevalence rate of GORD in 2019 also exhibited the highest YLDs (Figure S1). The detailed information was described in the Supplementay results.

### Distribution of incidence, prevalence and YLDs by age and sex

Globally, the ASIR of GORD per 100,000 population over time showed decreased incidence rate from 1990 to 2010, followed by an increase from 2010 to 2019 (Figure S4). Similarly, the time-incidence trends in countries with High SDI and High-middle were more pronounced. Notably, the ASIR in Middle SDI countries has been rising steadily (Figure S4). A similar trend can be seen in ASPR and ASYLDs (Figure S4). Globally, the ASIR, ASPR and ASYLDs of GORD increased slightly both in males and females. By sex subgroup, females had a higher the ASIR, ASPR and ASYLDs of GORD over time than in males in all SDI quintiles (Figure S4). At all times, low SDI quintiles have the highest ASPR, ASIR, and ASYLDs during 1990 to 2019 for both sexes. For females, Middle SDI quintiles had the lowest ASIR, ASPR and ASYLDs in 1990, but now in 2019, High SDI quintiles have the lowest values. For males, Middle SDI quintiles have the lowest ASIR, ASPR and ASYLDs in 1990, but now in 2019, High SDI quintiles have the lowest ASPR and ASYLDs, and High-middle SDI had the lowest ASIR. The ASIR, ASPR and ASYLDs in 1990 and 2019 all increased initially with age, peaking at 70–74, 70–74, and 60–64 years of age respectively, and then decreased overall and for both sexes (Figure S5). Males older than 89 years had a higher ASIR, ASPR and ASYLDs than females (Figure S5).

### The SDI and age-standardized rate correlation in 2019

We evaluated the coefficient of Pearson correlation among SDI level and age-standardized rate in 2019. ASIR (*ρ* = -0.179, *p* = 0.010), ASPR (*ρ* = -0.163, *p* = 0.020) and ASYLDs (*ρ* = -0.159, *p* = 0.023) in 2019 was correlated negatively with SDI (Figure S6). These findings suggested that the ASIR, ASPR and ASYLDs of countries with higher SDI in 2019 might show a steeper trend of decline.

### GORD epidemiology is driven by population growth, aging, and epidemiological changes

**Fig. 2:**
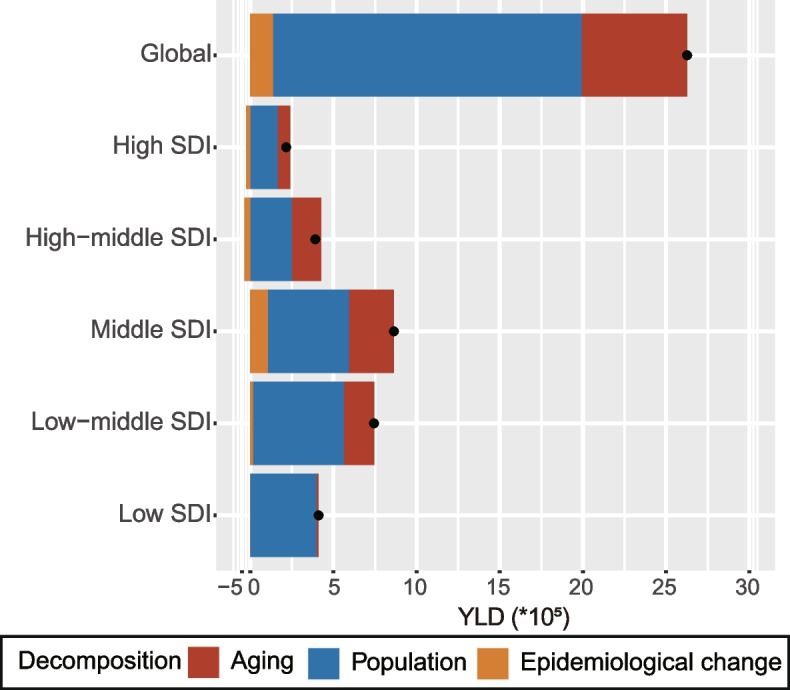
At the global level and by SDI quintile, changes in GORD YLDs as influenced by factors such as population growth, aging, and epidemiological change. A positive magnitude indicates an increase in GORD YLDs attributable to the component; a negative magnitude indicates a decrease in GORD YLDs attributable to the component. GORD, Gastro-oesophageal reflux disease; YLDs, Years lived with disability; SDI, socio-demographic index

Table S5 presents decomposition analyses by population growth, aging, and epidemiologic changes at the global, SDI quintiles, regional and national levels. Globally and for each SDI quintile, GORD YLDs increased significantly in the past 30 years but it was most pronounced among the Middle SDI and Low-middle SDI quintiles, where the YLD increase was the largest (Fig. 2). GORD YLD burden increased by 70.65% following population growth and 24.12% following aging of the world population between 1990 and 2019 (Table S5). YLDs contributed most substantially to aging in the High-middle SDI (45.01%) and declined in the High SDI (35.34%), Middle SDI (31.35%), Low-middle SDI (24.27%), and Low-SDI quintile (3.16%). A significant amount of the increase in GORD YLDs came from population growth in countries with a Low-SDI quintile (96.89%) and High-SDI quintile (75.98%); population growth was a smaller contributor to the increase in GORD YLDs in Middle-SDI countries (56.44%). Over the past 30 years, differences in morbidity and mortality rates between SDI quintiles can be traced to changes in morbidity and mortality associated with underlying changes in age and population. The decrease was least pronounced in High SDI, High-middle SDI, and low-SDI quintiles, while the increase was obvious in other two SDI quintiles (Table S5). The demographic and epidemiologic trends were considerably heterogeneous across GBD regions. GORD YLDs in most GBD regions changed significantly due to aging and population growth (Table S5). The global trend of increasing epidemiological changes was also evident in most GBD regions. In several GBD regions, epidemiological changes decreased, including High-income North America (-52.18%), Western Europe (-0.47%), Australasia (-0.2%), Tropical Latin America (-0.69%), Southern Latin America (-0.21%), Caribbean (-0.37%), Central Asia (-0.11%), East Asia (-0.32%), Oceania (-0.1%), and Southern Sub-Saharan Africa (-0.56%) (Table S5).

### Frontier analysis for the relationship between YLDs of GORD and status of the country’s development

**Fig. 3:**
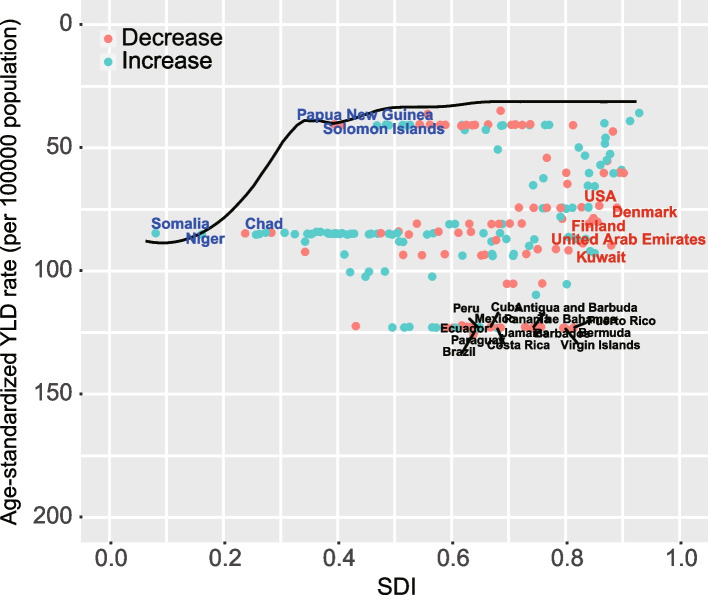
Frontier analysis based on SDI and ASYLDs of GORD in 2019. Black solid lines delineate the frontier; black dots indicate the countries and territories.Black represents the top 15 countries with the largest effective difference (largest GORD DALYs gap from the frontier) while blue represents frontier countries with low SDI (< 0.45) and low effective difference (e.g., Somalia, Niger, Chad, Solomon Islands, Papua, and New Guinea), and red label indicates countries and territories with high SDI (> 0.85) and relatively high effective difference for their level of development (e.g., USA, Finland, Denmark, United Arab Emirates, and Kuwait). A Red dot indicates an increase in age-standardized GORD ASYLDs rates between 1990 and 2019; a blue dot indicates a decrease. GORD, Gastro-oesophageal reflux disease; YLDs, Years lived with disability; SDI, socio-demographic index; ASYLDs, age-standardized YLDs

Based on Frontier analysis, ASYLDs and SDI data in 2019 was built to explore the relationship between the YLDs rates of GORD and country’s development status (Fig. 3). Frontier lines indicate the areas with the lowest YLD rates (optimal performers) based on their SDI. A country's effective Distance from the frontier is defined as the gap between a country’s observed and potentially achievable YLDs; this gap can be reduced or eliminated based on the country or territory’s sociodemographic resources. In 2019, the SDI and YLDs were used to calculate the effective difference between each country and territory. (Fig. 3, Table S6). As SDI increased, the effective difference tends to be smaller and less variable. There were 10 countries with the highest effective difference from the frontier (range of effective difference: 94.4–91.39) Paraguay, Brazil, Bermuda, Barbados, Peru, Virgin Islands, Costa Rica, The Bahamas, Jamaica, and Panama; these countries have highly elevated GORD YLDs rates compared to other countries with comparable sociodemographic resources. Compared to their place on the development spectrum and thus their effective difference, these 10 countries have the lowest YLDs rates (range: 0.14–4.52) included Somalia, Papua New Guinea, Solomon Islands, Niger, Chad, Mali, Burkina Faso, North Korea, China, and Switzerland.

### Patient and public involvement

Patient and public involvement statement is not applicable in this paper since the patients or the public were not involved in either the design, conduct, reporting or dissemination plans of our research.

## Discussion

This study furnishes the most updated determinations of GORD burden in 204 nations and territories from 1990 to 2019. Herein, we assessed the incidence, prevalence, and YLDs of GORD at global, SDI quintile, regional, and national levels. In 2019, the estimates for GORD were 6.0 million YLDs, 783 million prevalent cases, and 309 million incident cases. Change in GORD burden varies with development and geography. For instance, Tropical Latin America has the highest incidence rate of GORD, while the lowest incidence rate was observed in East Asia. The wide geographical differences may be related to several factors, including obesity, alcohol, smoking, ageing, and race [1, 4, 20]. Importantly, the Asian continent is large and heterogenous with possibly significant differences in dietary and socio-economic factors that can influence the GORD burden, compared to what was expected, especially in a few regions, such as North Africa and the Middle East, and South Asia. Our analyses revealed several significant findings. First, the ASIR, ASPR and ASYLDs increased from 1990 to 2019 at the global level, emphasizing the impact of the GORD burden worldwide. The observations were likewise in previous studies, suggesting an increased GORD burden with time and predicting it as one of the primary reasons for global YLDs [13]. Thus, it is important to prioritize GORD management, prevention measures, and treatment. ASIR of GORD is estimated to be highest in Brazil, Paraguay, Latin American nations, the Caribbean, and South Asia. Second, decomposition of GORD YLDs on the other hand, showed that the increase was mostly driven by population growth and aging, and was tempered (though not completely offset) by decreases in epidemiological changes in several GBD regions including High-income North America, Western Europe, Australasia, Tropical Latin America, Southern Latin America, Caribbean, Central Asia, East Asia, Oceania, and Southern Sub-Saharan Africa. Efforts to reduce the burden of GORD are available at all levels of development, but the burden is borne most heavily by countries with the least resources. Compared to most other regions and the global trend, from 1990 to 2019, High-income Asia Pacific and Central Europe showed increased rates of ASYLDs due to GORD. Beyond just demographic changes, these regions may have factors adding to the increase in the burden of GORD. Third, GORD has a greater impact on poorer, less well-developed countries and territories. Fourth, frontier analysis indicates that many low-income countries are leading the field in reducing GORD YLDs even at the low end of the development spectrum. Typical cases of success, indicating that a country or territory with a different development status should not be discouraged from enacting policies and leveraging available resources to reduce its GORD burden.

For GBD regions, ASPR of GORD calculated using the data from the previous 2017 GBD Study has been reported [15]. The earlier report did not mention AAPC and the ASIR for GORD burden from 1990 to 2019. To date, published studies about GORD incidence are very limited. GORD incidence of 0.84 per 1000 person-years in pediatric patients of ages 1–17 years in England from 2000 to 2005 was reported by Ruigómez et al. [21]. Other systematic reviews have only discussed GORD incidence and prevalence for some selected regions and nations [4, 13, 14]. ASIR of GORD is estimated to be lowest in China, concurrent with the findings by El-Serag et al. and Nirwan et al. [13, 14]. However, these studies did not discuss a temporal difference for post-1995 published studies. Notably, our analysis results showed that the USA showed significant improving trends in ASIR, ASPR and ASYLDs for GORD. This is a novel observation for GORD burden changes. The following may be possible reasons. First, a negative association was found between GORD burden and SDI values in this study; this has not been previously reported, which may be due to the higher effective recognition, increased attention and management of GORD in high SDI countries. Secondly, Advances in medical science and technology (these include the development of new drugs, the improvement of diagnosis and treatment methods, and the real-time update of diagnosis and treatment guidelines) are largely responsible for the improving trends of GORD burden [22–24].

This study found that countries with less developed economies are more heavily burdened with the cost of GORD. Additionally, ADYLD rates were inversely related to SDI. Despite an optimistic assessment of our frontier analysis, many countries on all scales of development have YLDs that are distant from the frontier (with a large effective difference from the frontier), suggesting unrealized possibilities to close the YLD gap.Even though frontier countries exist at all SDI levels, those with low SDIs are most noteworthy for their outstanding performance despite limited resources. There might be a role for these countries as typical cases for optimizing the health outcomes in low-resource economics. The observation that several high SDI countries have lagged behind other countries (e.g., Kuwait, United Arab Emirates) suggests that health progress enabled by sociodemographic prosperity may be overcome by other factors. In the future, it will be important to identify the factors contributing to success in exemplar countries and the factors impeding progress in laggard countries; filling this knowledge gap will likely aid in alleviating the GORD burden.

The study also has certain limitations. First, although the burden of GORD was analyzed globally, regionally, and nationally, it was not assessed at the local level; besides, rural–urban-rural discrepancies were also not considered. The gaps between examinations at national/ provincial/ district levels were limited to measuring a shift in the local disease burden and specifically and effectively influencing public policies, which emphasizes the need for studies at the district level. Second, the heterogeneous nature of estimation of GORD makes the comparison of estimates across regions and populations difficult, and it may cause disarranged classifications [19]. Definitions applied for individual studies have not been reported, and data used for analysis by DisMod-MR 2.1 were from reports that included GORD diagnosis by self-reported diagnosis, diagnostic codes, and symptom-based questionnaire surveys [15]. At the same time, potential biases were partially overcome in the framework of DisMod modeling. In the DisMod modeling, adjustments were modeled as difference in logit prevalence between alternative and reference data. The estimated mean logit differences were applied to non-reference data types as bias correction prior to modeling. Finally, because the data of cause factors are not available for GORD, we did not report the contribution of cause factors to the change of GORD burden.

In conclusion, GORD is a public health challenge with increasing ASIR, ASPR, and YLDs rates globally in the past 30 years. The ASIR, ASPR, and YLDs of GORD vary between countries. A rising population and aging have largely contributed to the increase in YLD due to GORD. GORD burden is more heavily skewed toward less developed countries due to significant variations in demographics and epidemiology by geographic region. By the increase in the number of GORD patients, healthcare infrastructure needs to be revamped to address associated needs. Mitigation of the burden of GORD in the future requires enhanced awareness, GORD should be diagnosed and treated early, particularly for risk factors. Given the adverse outcomes and costs of symptomatic treatment for GORD and an increase in the risk of oesophageal carcinoma in GORD-affected people, it is crucial to continuously expand the collection of high-quality and large GORD population-based data to assess the burden of disease and to determine if enhanced care is necessary. There should be a global and national health agenda that takes into account the growing burden of GORD.

## Supplementary Information

Additional file 1:  **Figure S1. **Incidence, prevalence, and YLDs due to GORD for all GBD regions, 1990-2019. YLDs, Yearslived with disability; GORD, Gastro-oesophageal reflux disease. **Figure S2.** The prevalence of GORD for both sexes in 204 countries and territories. (**A**) The ASPR of GORD in 2019; (**B**) The AAPC of ASPR of GORD from 1990 to 2019. GORD, Gastro-oesophagealreflux disease; ASPR, age-standardized prevalence rate; AAPC, average annualpercentage change. **Figure S3.** A list of the YLDs for 204 countries and territories for both sexes. (**A**) The ASYLDs of GORD in 2019; (**B**) The AAPC of ASYLDs of GORD from 1990 to 2019. GORD, Gastro-oesophageal reflux disease; ASYLDs, age-standardized YLDs; AAPC, average annual percentage change; YLDs, Years lived withdisability. **Figure S4.** Comparison of thechanging trends of GORD rates between different SDI quintiles and sexes. GORD, Gastro-oesophageal reflux disease; SDI, socio-demographic index. **Figure S5.** ASIR, ASPR, and ASYLDs (per 100000 population) of GORD, by age group, in 1990 and 2019. ASIR, age-standardized incidence rate; ASIR, age-standardized incidence rate; ASYLDs, age-standardized YLDs; GORD, Gastro-oesophageal reflux disease. **Figure S6.** The correlation between SDI and age-standardized rate in 2019. SDI, socio-demographic index, YLDs, Years lived with disability.

Additional file 2:  **Table S1.** Incidence of gastro-oesophageal reflux disease in 1990 and 2019 with AAPC from 1990 and 2019 at countries/territories level, both sexs.

Additional file 3:  **Table S2.** Prevalence of gastro-oesophageal reflux disease in 1990 and 2019 for both sexes and all locations, with AAPC from 1990 and 2019.

Additional file 4:  **Table S3.** Prevalence of gastro-oesophageal reflux disease in 1990 and 2019 with AAPC from 2009 and 2019 at countries/territories level, both sexs.

Additional file 5:  **Table S4.** YLDs of gastro-oesophageal reflux disease in 1990 and 2019 with AAPC from 1990 and 2019 at national level, both sexs.

Additional file 6:  **Table S5.** Changes in YLDs number according to population-level determinants and causes from 1990 to 2019.

Additional file 7: **Table S6.** Frontier YLDs, and effective difference by country or territory.

## Data Availability

The datasets generated during the current study are available in the Global Health Data Exchange query tool (http://ghdx.healthdata.org/gbd-​results-​tool).
