# Supplementary material for: Burden of gastroesophageal reflux disease in 204 countries and territories, 1990–2019: a systematic analysis for the Global Burden of disease study 2019
Source: BMC Public Health. 2023 Mar 29;23:582. doi: 10.1186/s12889-023-15272-z (PMC10053627; doi:10.1186/s12889-023-15272-z)
Supplement: Supplementary file 1 — Figure S1. Incidence, prevalence, and YLDs due to GORD for all GBD regions, 1990-2019. YLDs, Yearslived with disability; GORD, Gastro-oesophageal reflux disease. Figure S2. The prevalence of GORD for both sexes in 204 countries and territories. (A) The ASPR of GORD in 2019; (B) The AAPC of ASPR of GORD from 1990 to 2019. GORD, Gastro-oesophagealreflux disease; ASPR, age-standardized prevalence rate; AAPC, average annualpercentage change. Figure S3. A list of the YLDs for 204 countries and territories for both sexes. (A) The ASYLDs of GORD in 2019; (B) The AAPC of ASYLDs of GORD from 1990 to 2019. GORD, Gastro-oesophageal reflux disease; ASYLDs, age-standardized YLDs; AAPC, average annual percentage change; YLDs, Years lived withdisability. Figure S4. Comparison of thechanging trends of GORD rates between different SDI quintiles and sexes. GORD, Gastro-oesophageal reflux disease; SDI, socio-demographic index. Figure S5. ASIR, ASPR, and ASYLDs (per 100000 population) of GORD, by age group, in 1990 and 2019. ASIR, age-standardized incidence rate; ASIR, age-standardized incidence rate; ASYLDs, age-standardized YLDs; GORD, Gastro-oesophageal reflux disease. Figure S6. The correlation between SDI and age-standardized rate in 2019. SDI, socio-demographic index, YLDs, Years lived with disability. [file 12889_2023_15272_MOESM1_ESM.docx]

**Supplemental Figures:**


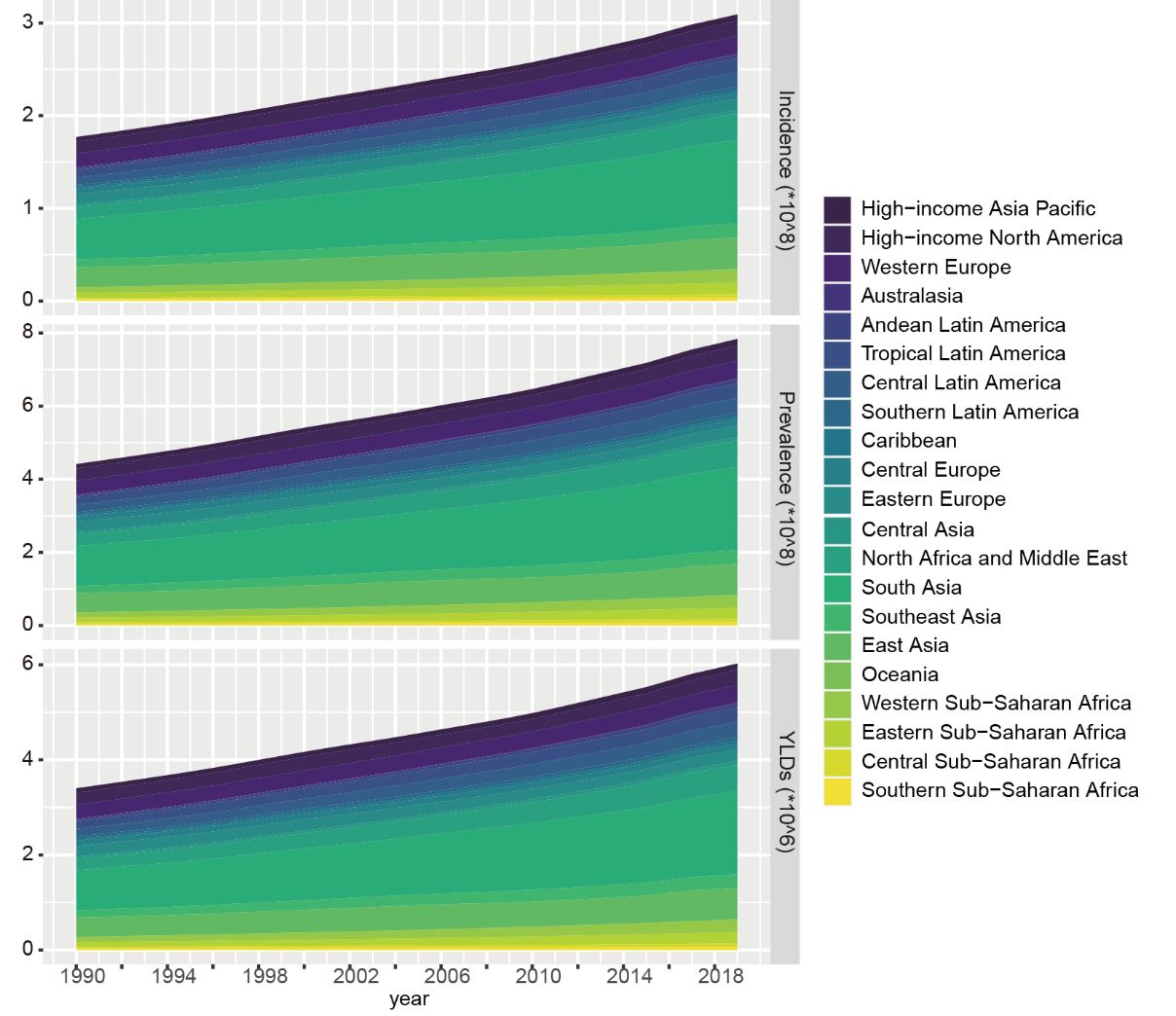


Figure S1. Incidence, prevalence, and YLDs due to GORD for all GBD regions, 1990-2019. YLDs, Years lived with disability; GORD, Gastro-oesophageal reflux disease;


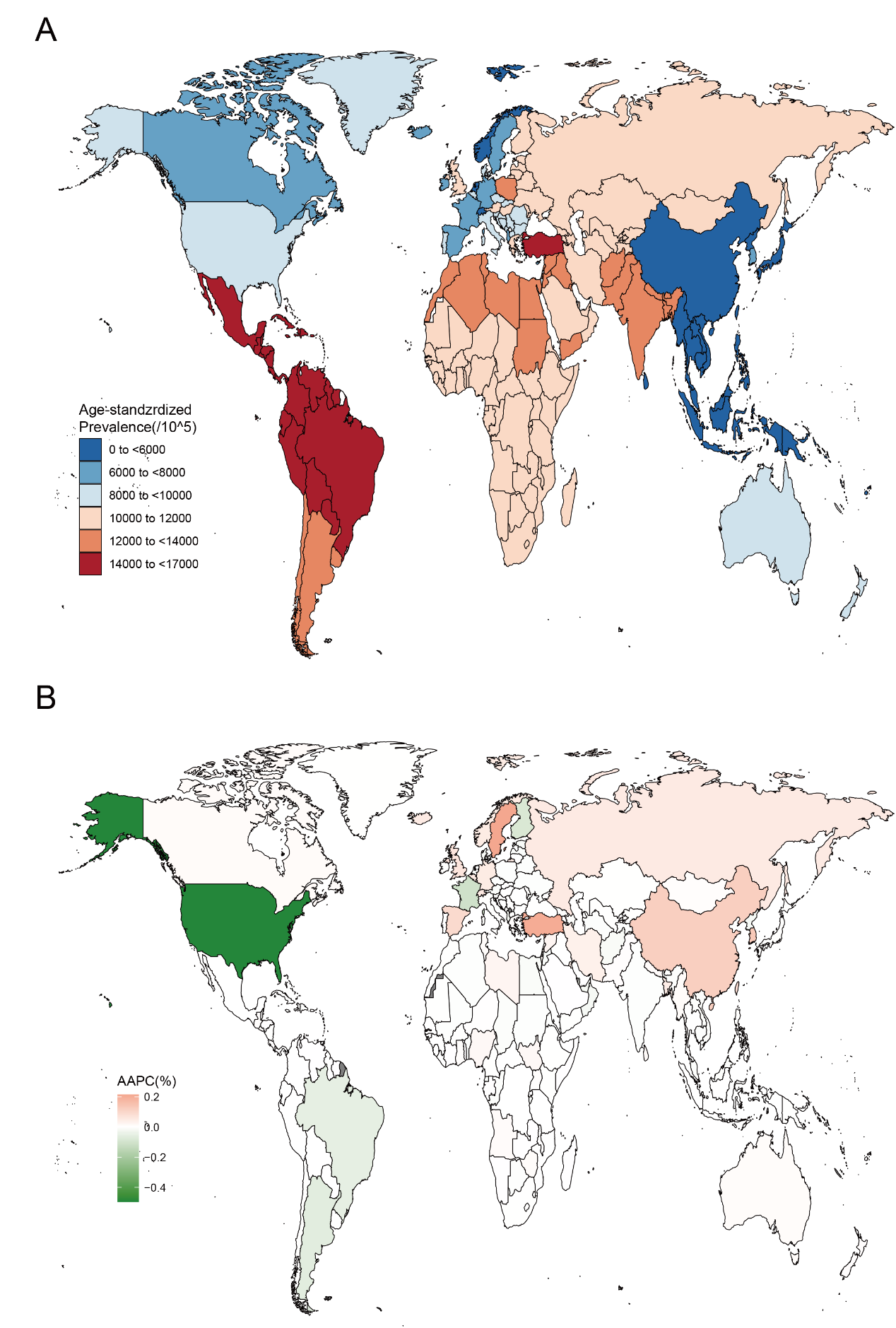


Figure S2. The prevalence of GORD for both sexes in 204 countries and territories. (A) The ASPR of GORD in 2019; (B) The AAPC of ASPR of GORD from 1990 to 2019. GORD, Gastro-oesophageal reflux disease; ASPR, age-standardized prevalence rate; AAPC, average annual percentage change;


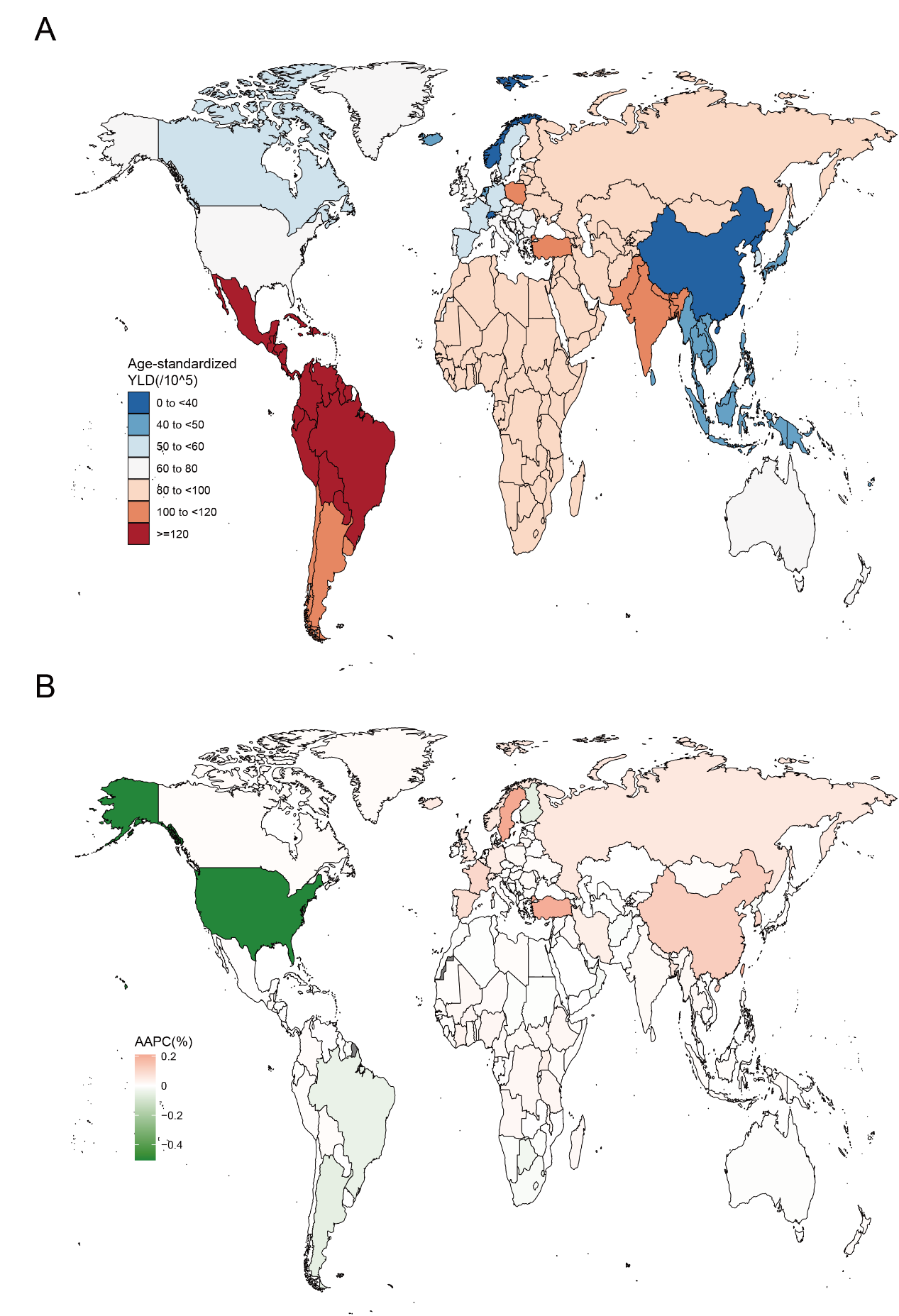


Figure S3. A list of the YLDs for 204 countries and territories for both sexes. (A) The ASYLDs of GORD in 2019; (B) The AAPC of ASYLDs of GORD from 1990 to 2019. GORD, Gastro-oesophageal reflux disease; ASYLDs, age-standardized YLDs; AAPC, average annual percentage change; YLDs, Years lived with disability.


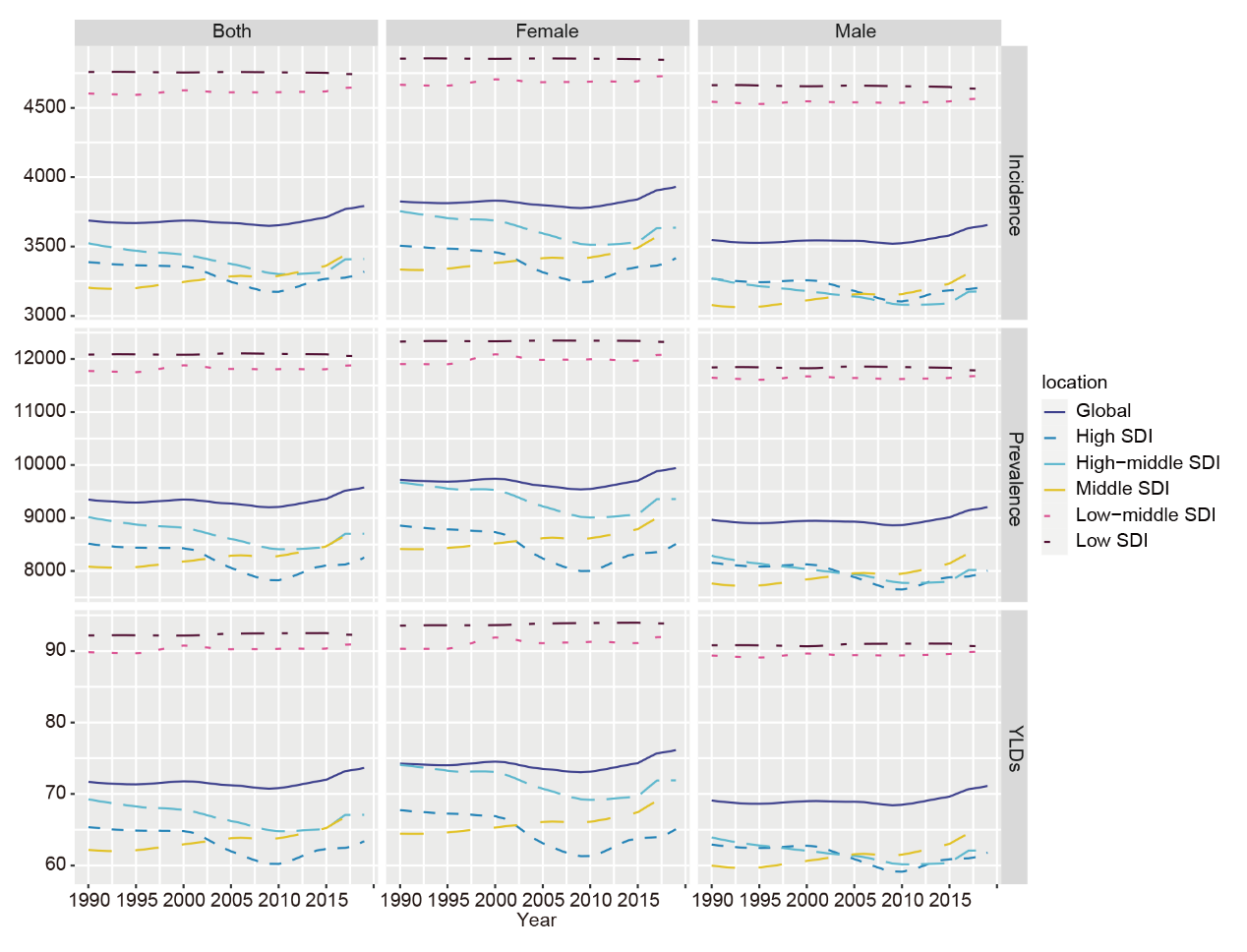


Figure S4. Comparison of the changing trends of GORD rates between different SDI quintiles and sexes. GORD, Gastro-oesophageal reflux disease; SDI, socio-demographic index


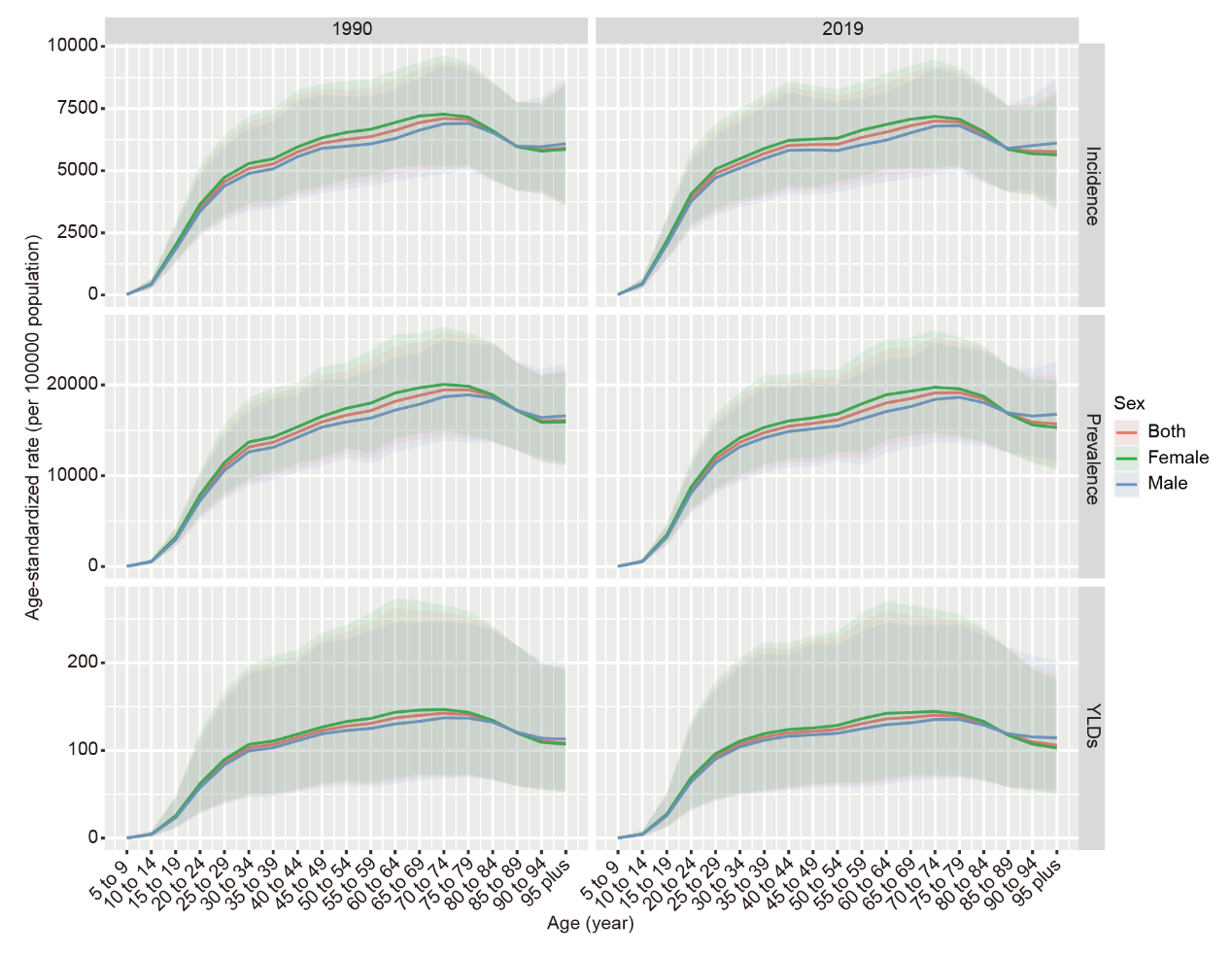


Figure S5. ASIR, ASPR, and ASYLDs (per 100000 population) of GORD, by age group, in 1990 and 2019

ASIR, age-standardized incidence rate; ASIR, age-standardized incidence rate; ASYLDs, age-standardized YLDs; GORD, Gastro-oesophageal reflux disease


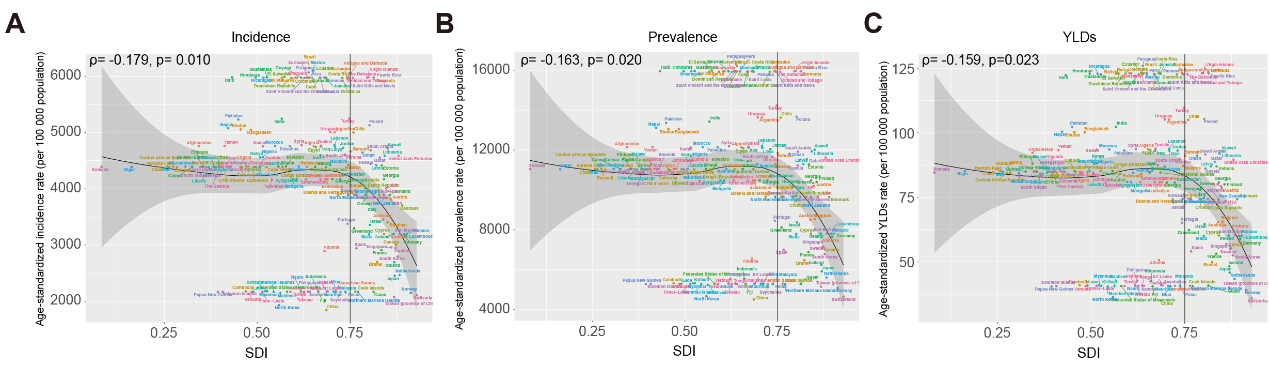


Figure S6. The correlation between SDI and age-standardized rate in 2019.

SDI, socio-demographic index, YLDs, Years lived with disability.
