# Supplementary material for: Burden of gastroesophageal reflux disease in 204 countries and territories, 1990–2019: a systematic analysis for the Global Burden of disease study 2019
Source: BMC Public Health. 2023 Mar 29;23:582. doi: 10.1186/s12889-023-15272-z (PMC10053627; doi:10.1186/s12889-023-15272-z)
Supplement: Supplementary file 2 — Table S1. Incidence of gastro-oesophageal reflux disease in 1990 and 2019 with AAPC from 1990 and 2019 at countries/territories level, both sexs. [file 12889_2023_15272_MOESM2_ESM.docx]

Table S1. Incidence of gastro-oesophageal reflux disease in 1990 and 2019 with AAPC from 1990 and 2019 at countries/territories level, both sexs.

| Countries/territories | 1990 | |  | 2019 | | AAPC % (95% CI)  1990-2019 |
| --- | --- | --- | --- | --- | --- | --- |
|  | Cases (95% UI) | Age-standardised incidence per  100 000 population (95% UI) |  | Cases (95% UI) | Age-standardised incidence per  100 000 population (95% UI) |  |
| Afghanistan | 397567 (349403 to 452058) | 4777.45 (4210.21 to 5409.59) |  | 1249710 (1066175 to 1441362) | 4757.01 (4205.1 to 5398.9) | -0.0148 (-0.0167 to -0.0129) |
| Albania | 81624 (70557 to 94398) | 2891.45 (2530.97 to 3353.77) |  | 97033 (85416 to 112503) | 2891.51 (2529.93 to 3355.75) | -0.0164 (-0.0397 to 0.0069) |
| Algeria | 890749 (770356 to 1017267) | 4759.17 (4202.9 to 5398.58) |  | 1995109 (1733398 to 2282525) | 4755.25 (4196.58 to 5393.53) | -0.003 (-0.0032 to -0.0027) |
| American Samoa | 814 (697 to 943) | 2165.3 (1892.29 to 2492.67) |  | 1142 (994 to 1324) | 2169.82 (1895.83 to 2500.76) | 0.0072 (0.0069 to 0.0075) |
| Andorra | 1990 (1714 to 2287) | 3180.29 (2790.71 to 3641.68) |  | 3558 (3096 to 4123) | 3186.49 (2799.2 to 3648.99) | 0.0068 (-0.0017 to 0.0153) |
| Angola | 302103 (261280 to 349279) | 4382.31 (3847.7 to 5001.96) |  | 881387 (763978 to 1019730) | 4392.67 (3856.26 to 5013.4) | 0.0083 (0.0081 to 0.0084) |
| Antigua and Barbuda | 3407 (3006 to 3800) | 5980.2 (5314.28 to 6642.27) |  | 6103 (5427 to 6784) | 5977.25 (5314.03 to 6640.36) | -0.0017 (-0.0019 to -0.0014) |
| Argentina | 1605119 (1413897 to 1822782) | 5002.85 (4406.29 to 5686.9) |  | 2453344 (2164558 to 2787569) | 5002.64 (4405.97 to 5687.14) | -0.0366 (-0.0983 to 0.0251) |
| Armenia | 133997 (116683 to 153084) | 4170.03 (3660.47 to 4766.45) |  | 149541 (131343 to 171178) | 4170.83 (3658.86 to 4767.61) | 0.0009 (0.0006 to 0.0012) |
| Australia | 639302 (559408 to 735788) | 3453.92 (3015.85 to 3982.81) |  | 1035239 (915228 to 1185014) | 3454.36 (3017.22 to 3984.12) | -0.0072 (-0.1361 to 0.1219) |
| Austria | 359473 (314928 to 411707) | 3843.76 (3362.55 to 4401.33) |  | 448621 (393919 to 514289) | 3835.12 (3352.42 to 4392.28) | -0.0081 (-0.0109 to -0.0053) |
| Azerbaijan | 266600 (231450 to 303680) | 4172.63 (3663.66 to 4769.28) |  | 468339 (407864 to 538737) | 4166.36 (3657.56 to 4763.66) | -0.005 (-0.0052 to -0.0048) |
| Bahamas | 13914 (12163 to 15698) | 5978.16 (5312.62 to 6641.65) |  | 25126 (22252 to 28038) | 5978.01 (5313.75 to 6641.46) | -0.0001 (-0.0002 to 0) |
| Bahrain | 21255 (17877 to 24797) | 4683.44 (4135.86 to 5318.43) |  | 79970 (67812 to 93652) | 4663.17 (4108.43 to 5293.58) | -0.0168 (-0.0194 to -0.0141) |
| Bangladesh | 3771438 (3261934 to 4315596) | 5022.84 (4434.42 to 5665.15) |  | 7847981 (6859982 to 8904964) | 5048.8 (4458.27 to 5688.97) | 0.028 (-0.2075 to 0.264) |
| Barbados | 15827 (14049 to 17611) | 5978.13 (5311.18 to 6638.99) |  | 21778 (19607 to 24007) | 5977.13 (5312.89 to 6640.46) | -0.0005 (-0.0006 to -0.0004) |
| Belarus | 517681 (453556 to 587431) | 4429.58 (3890.17 to 5038.63) |  | 535101 (470363 to 608705) | 4424.32 (3886.63 to 5032.37) | -0.0037 (-0.0042 to -0.0033) |
| Belgium | 406503 (356380 to 464843) | 3369.15 (2951.5 to 3843.31) |  | 495004 (436757 to 567139) | 3401.3 (2983.24 to 3886.2) | 0.0334 (0.0183 to 0.0486) |
| Belize | 8054 (7039 to 9114) | 5971.16 (5313.77 to 6640) |  | 22966 (20210 to 25691) | 5975.75 (5316.65 to 6643.92) | 0.0027 (0.0026 to 0.0028) |
| Benin | 133529 (116026 to 153702) | 4393.83 (3856.02 to 5016.53) |  | 372498 (322402 to 430817) | 4389.48 (3852.66 to 5009) | -0.0034 (-0.0035 to -0.0033) |
| Bermuda | 4040 (3567 to 4508) | 5976.64 (5311.7 to 6642.42) |  | 4985 (4484 to 5487) | 5976.1 (5313.1 to 6644.38) | -0.0002 (-0.0003 to -0.0001) |
| Bhutan | 21942 (19035 to 25043) | 5054.86 (4472.93 to 5709.14) |  | 37543 (32754 to 42689) | 5054.18 (4472.86 to 5708.38) | -0.0014 (-0.0024 to -0.0004) |
| Bolivia (Plurinational State of) | 289092 (253290 to 324974) | 5977.76 (5314.49 to 6643.04) |  | 658187 (581012 to 735276) | 5973.67 (5312.78 to 6642.31) | -0.0024 (-0.0025 to -0.0024) |
| Bosnia and Herzegovina | 183963 (159835 to 212494) | 3896.78 (3409.43 to 4469.33) |  | 170007 (149661 to 195552) | 3894.68 (3405.39 to 4468.33) | -0.0017 (-0.002 to -0.0015) |
| Botswana | 39450 (34284 to 45393) | 4395.25 (3856.79 to 5017) |  | 94590 (81487 to 109373) | 4390.92 (3856.22 to 5012.28) | -0.0033 (-0.0034 to -0.0031) |
| Brazil | 7925275 (6981949 to 8834619) | 6167.02 (5487.94 to 6809.26) |  | 14749241 (13093179 to 16334074) | 6146.6 (5457.64 to 6790.57) | -0.0403 (-0.084 to 0.0034) |
| Brunei Darussalam | 5605 (4742 to 6559) | 2700.79 (2356.13 to 3100.31) |  | 12435 (10676 to 14430) | 2701.6 (2357.39 to 3103.17) | 0.0012 (0.0008 to 0.0016) |
| Bulgaria | 403310 (355269 to 462941) | 3894.65 (3405.19 to 4467.12) |  | 369130 (325800 to 423191) | 3894.12 (3406.49 to 4468.09) | -0.0006 (-0.001 to -0.0003) |
| Burkina Faso | 268874 (234927 to 308938) | 4395.64 (3855.53 to 5016.89) |  | 671154 (583276 to 773754) | 4393.83 (3857.17 to 5015.14) | -0.0014 (-0.0016 to -0.0012) |
| Burundi | 160774 (139561 to 184925) | 4391.63 (3856.94 to 5013.61) |  | 353899 (306082 to 409189) | 4381.5 (3844.19 to 5002.99) | -0.0082 (-0.0088 to -0.0076) |
| Cabo Verde | 10952 (9616 to 12445) | 4401.36 (3863.79 to 5022.8) |  | 23764 (20681 to 27336) | 4384.64 (3852.93 to 5004.27) | -0.013 (-0.0134 to -0.0126) |
| Cambodia | 155280 (133311 to 179597) | 2181.54 (1904.56 to 2516.26) |  | 336998 (290966 to 387606) | 2177.43 (1901.74 to 2509.55) | -0.0065 (-0.0067 to -0.0063) |
| Cameroon | 304462 (264537 to 351052) | 4388.58 (3851.46 to 5010.25) |  | 925636 (800082 to 1069165) | 4386.36 (3849.96 to 5007.13) | -0.0018 (-0.0019 to -0.0017) |
| Canada | 927502 (801126 to 1071717) | 3002.75 (2610.45 to 3488.08) |  | 1401982 (1227210 to 1617608) | 3002.14 (2611.14 to 3486.74) | 0.008 (-0.0072 to 0.0232) |
| Central African Republic | 83159 (72104 to 96197) | 4390.89 (3853.39 to 5011.74) |  | 166700 (144420 to 192997) | 4391.56 (3854 to 5011.56) | 0.0007 (0.0003 to 0.0012) |
| Chad | 171333 (149652 to 196630) | 4392.01 (3854.44 to 5013.78) |  | 429179 (372304 to 495633) | 4383.51 (3844.65 to 5003.51) | -0.0068 (-0.007 to -0.0066) |
| Chile | 623454 (543614 to 711688) | 5003.91 (4407.65 to 5688.4) |  | 1057246 (934447 to 1200729) | 5001.41 (4405.04 to 5685.64) | -0.0018 (-0.0019 to -0.0017) |
| China | 20851665 (17977319 to 24138125) | 1849.31 (1612.82 to 2144.21) |  | 32711266 (28441817 to 38211687) | 1841.66 (1607.09 to 2133.51) | 0.11 (-0.0712 to 0.2915) |
| Colombia | 1636694 (1430804 to 1847223) | 5977.37 (5315.9 to 6645.27) |  | 3083033 (2748258 to 3432735) | 5976.3 (5312.29 to 6639.16) | -0.0006 (-0.0009 to -0.0003) |
| Comoros | 13806 (12071 to 15884) | 4387.63 (3851.52 to 5007.67) |  | 27816 (24212 to 31877) | 4385.92 (3853.23 to 5005.87) | -0.0013 (-0.0014 to -0.0012) |
| Congo | 72786 (63285 to 83777) | 4391.35 (3858 to 5011.82) |  | 186710 (161374 to 215371) | 4385.94 (3850.31 to 5005.8) | -0.0042 (-0.0043 to -0.0041) |
| Cook Islands | 350 (303 to 404) | 2162.68 (1889.76 to 2491.02) |  | 439 (386 to 511) | 2171.96 (1897.5 to 2505.54) | 0.0148 (0.0127 to 0.017) |
| Costa Rica | 151882 (132913 to 171142) | 5974.99 (5314.22 to 6643.88) |  | 309328 (275147 to 344444) | 5978.8 (5314.63 to 6641.03) | 0.0022 (0.0021 to 0.0023) |
| Croatia | 224031 (196623 to 256493) | 3897.53 (3410.62 to 4471.76) |  | 222584 (196571 to 255293) | 3894.29 (3405.56 to 4467.68) | -0.0029 (-0.003 to -0.0028) |
| Cuba | 673405 (596681 to 751931) | 5973.1 (5313.38 to 6642.92) |  | 843400 (760721 to 929595) | 5971 (5310.48 to 6636.25) | -0.0012 (-0.0013 to -0.0012) |
| Cyprus | 26163 (22968 to 30047) | 3195.7 (2812.12 to 3658.88) |  | 52608 (46063 to 60402) | 3197.07 (2813.2 to 3663.14) | 0.0014 (0.0012 to 0.0016) |
| Czechia | 461060 (404128 to 528264) | 3897.14 (3408.54 to 4471.57) |  | 550878 (485893 to 631231) | 3892.82 (3404.95 to 4466.35) | -0.0039 (-0.004 to -0.0037) |
| Côte d'Ivoire | 347459 (298596 to 403287) | 4377.04 (3841.62 to 4997.72) |  | 852732 (736093 to 985620) | 4378.41 (3843.18 to 4998.84) | 0.001 (0.0009 to 0.0011) |
| Democratic People's Republic of Korea | 381612 (331691 to 444777) | 1942.59 (1705.23 to 2258.21) |  | 588799 (515161 to 684949) | 1931.72 (1691.95 to 2244.82) | -0.0197 (-0.0205 to -0.0189) |
| Democratic Republic of the Congo | 1112988 (966754 to 1284652) | 4389.12 (3856.92 to 5010.16) |  | 2708808 (2345608 to 3131081) | 4386.95 (3852.36 to 5006.26) | -0.0018 (-0.0019 to -0.0017) |
| Denmark | 231516 (203609 to 263387) | 3711.16 (3252.88 to 4227.67) |  | 273256 (241058 to 310969) | 3702.28 (3249.03 to 4205.43) | -0.0076 (-0.0109 to -0.0043) |
| Djibouti | 13786 (11821 to 15978) | 4374.64 (3843.11 to 4990.07) |  | 45007 (38604 to 52107) | 4375.62 (3840.26 to 4991.62) | 0.0004 (-0.0003 to 0.001) |
| Dominica | 3970 (3521 to 4402) | 5971.21 (5304.35 to 6633.37) |  | 4602 (4137 to 5100) | 5970.34 (5312.53 to 6639.54) | -0.0004 (-0.0006 to -0.0003) |
| Dominican Republic | 344761 (300693 to 389489) | 5978.5 (5317.6 to 6645.49) |  | 644003 (568215 to 719018) | 5972.25 (5312.45 to 6639.67) | -0.0038 (-0.0041 to -0.0035) |
| Ecuador | 477047 (417654 to 537363) | 5975.69 (5315.28 to 6645.11) |  | 1027438 (909347 to 1144778) | 5974.8 (5313.32 to 6641.85) | -0.0006 (-0.0007 to -0.0005) |
| Egypt | 2082606 (1801115 to 2372991) | 4753.9 (4196.49 to 5393.19) |  | 4241181 (3683121 to 4822568) | 4740.61 (4181.78 to 5382.41) | -0.0098 (-0.0101 to -0.0095) |
| El Salvador | 244144 (214692 to 274514) | 5981.26 (5316.61 to 6645.23) |  | 372636 (329611 to 415272) | 5986.05 (5322.29 to 6649.26) | 0.0029 (0.0027 to 0.0031) |
| Equatorial Guinea | 12513 (10905 to 14376) | 4398.96 (3859.95 to 5019.48) |  | 44232 (37997 to 51144) | 4386.12 (3857.58 to 5002.7) | -0.0104 (-0.0109 to -0.0099) |
| Eritrea | 84617 (72952 to 98077) | 4393.35 (3858.34 to 5011.62) |  | 219852 (189949 to 254760) | 4389.07 (3856.26 to 5008.39) | -0.0033 (-0.0036 to -0.0031) |
| Estonia | 79038 (69531 to 89544) | 4430.27 (3890.54 to 5039.42) |  | 75480 (66721 to 85502) | 4415.54 (3879.77 to 5023.62) | -0.0112 (-0.012 to -0.0103) |
| Eswatini | 22413 (19420 to 25889) | 4397.96 (3857.25 to 5018.74) |  | 40704 (35308 to 46915) | 4395.94 (3860.01 to 5016.97) | -0.0016 (-0.0018 to -0.0014) |
| Ethiopia | 1490958 (1291036 to 1715812) | 4556.79 (3993.35 to 5178.06) |  | 3373821 (2914904 to 3909361) | 4554.23 (3990.07 to 5174.75) | -0.0019 (-0.0021 to -0.0017) |
| Fiji | 13262 (11395 to 15367) | 2168.09 (1894.39 to 2498.64) |  | 19238 (16674 to 22299) | 2169.26 (1895.7 to 2497.82) | 0.0019 (0.0017 to 0.0021) |
| Finland | 235340 (207173 to 269819) | 3936.68 (3462 to 4501.06) |  | 279493 (247361 to 318166) | 3884.74 (3410.25 to 4415.15) | -0.0685 (-0.127 to -0.01) |
| France | 1941879 (1707145 to 2229085) | 2909.44 (2549.9 to 3344.82) |  | 2430552 (2148719 to 2803854) | 2909.69 (2550.36 to 3345.32) | 0.076 (-0.2028 to 0.3555) |
| Gabon | 32070 (28051 to 36793) | 4386.77 (3853.02 to 5009.59) |  | 66604 (57847 to 76839) | 4390.16 (3849.71 to 5010.36) | 0.0027 (0.0025 to 0.0028) |
| Gambia | 27904 (24020 to 32295) | 4380.99 (3840.59 to 5001.27) |  | 71079 (61511 to 82050) | 4387 (3849.22 to 5006.91) | 0.0049 (0.0045 to 0.0052) |
| Georgia | 246260 (216695 to 280605) | 4174.83 (3665.79 to 4770.08) |  | 186920 (165393 to 212692) | 4169.41 (3658.71 to 4764.71) | -0.0044 (-0.0047 to -0.0041) |
| Germany | 3108826 (2731580 to 3562449) | 3114.15 (2722.26 to 3583.68) |  | 3612580 (3189649 to 4147672) | 3150.72 (2751.5 to 3630.15) | 0.0312 (-0.0376 to 0.1) |
| Ghana | 451892 (392469 to 521534) | 4388.09 (3851.67 to 5009.31) |  | 1131057 (981254 to 1305895) | 4393.45 (3857.03 to 5014.44) | 0.0044 (0.0041 to 0.0046) |
| Greece | 471176 (413508 to 540561) | 3844.37 (3355.17 to 4415.12) |  | 530752 (467403 to 609231) | 3843.86 (3354.83 to 4414.25) | 0.0034 (-0.0058 to 0.0127) |
| Greenland | 1833 (1567 to 2139) | 3296.09 (2868.75 to 3815.29) |  | 2158 (1883 to 2514) | 3297.26 (2873.28 to 3814.99) | 0.0007 (-0.0005 to 0.0019) |
| Grenada | 4280 (3794 to 4755) | 5975.09 (5309.87 to 6639.01) |  | 6784 (6057 to 7579) | 5969.69 (5309.95 to 6635.26) | -0.003 (-0.0031 to -0.0029) |
| Guam | 2669 (2284 to 3107) | 2161.78 (1888.95 to 2488.22) |  | 3854 (3371 to 4457) | 2165.74 (1892.42 to 2495.21) | 0.0066 (0.0051 to 0.008) |
| Guatemala | 336520 (295247 to 378855) | 5977.5 (5316.87 to 6645.78) |  | 942153 (824625 to 1060183) | 5981.35 (5314.81 to 6644.63) | 0.0023 (0.0022 to 0.0025) |
| Guinea | 190339 (166099 to 218177) | 4390.24 (3852.27 to 5011.14) |  | 378880 (329587 to 436618) | 4390.69 (3850.78 to 5011.5) | 0.0005 (0.0001 to 0.0009) |
| Guinea-Bissau | 28752 (24972 to 33141) | 4392.75 (3854.2 to 5014.29) |  | 59605 (51405 to 68942) | 4392.78 (3854.77 to 5013.07) | 0.0001 (0 to 0.0002) |
| Guyana | 37488 (32652 to 42448) | 5975.78 (5314.59 to 6644.15) |  | 45735 (40369 to 51158) | 5976.09 (5313.25 to 6642.57) | -0.0002 (-0.0008 to 0.0004) |
| Haiti | 288742 (253336 to 324421) | 5980.7 (5317.76 to 6648.42) |  | 649367 (568794 to 728868) | 5981.04 (5316.69 to 6643.2) | 0.0002 (-0.0003 to 0.0007) |
| Honduras | 192848 (168980 to 217523) | 5977.76 (5316.5 to 6645.27) |  | 514315 (450770 to 578060) | 5981.43 (5316.77 to 6644.18) | 0.0021 (0.002 to 0.0023) |
| Hungary | 492329 (428166 to 566801) | 4019.81 (3480.62 to 4620.88) |  | 522524 (457069 to 602159) | 4016.48 (3476.96 to 4617.74) | -0.0031 (-0.0036 to -0.0027) |
| Iceland | 7088 (6172 to 8118) | 2665.29 (2330.81 to 3072.6) |  | 11175 (9828 to 12874) | 2692.82 (2350.37 to 3115.51) | 0.0379 (0.0319 to 0.0439) |
| India | 35536943 (30952642 to 40366404) | 5247.11 (4653.88 to 5917.17) |  | 71837990 (63576583 to 81351721) | 5245.75 (4654.39 to 5915.26) | -0.0016 (-0.011 to 0.0077) |
| Indonesia | 3371805 (2893210 to 3927976) | 2250.39 (1955.33 to 2592.9) |  | 5997915 (5171530 to 6942484) | 2248.88 (1953.53 to 2591.93) | -0.0023 (-0.0024 to -0.0022) |
| Iran (Islamic Republic of) | 1898429 (1638735 to 2179475) | 4582.96 (4025.77 to 5215.86) |  | 4155278 (3576245 to 4751335) | 4590.85 (4033.41 to 5224.17) | 0.0281 (-0.0395 to 0.0958) |
| Iraq | 578636 (499104 to 660797) | 4754.45 (4198.76 to 5395.65) |  | 1764352 (1519980 to 2018301) | 4753.34 (4197.26 to 5393.37) | -0.0001 (-0.0021 to 0.0019) |
| Ireland | 117221 (103240 to 134378) | 3195.08 (2811.44 to 3657.24) |  | 186446 (163643 to 214582) | 3195.88 (2812.52 to 3659.72) | 0.0013 (-0.0001 to 0.0026) |
| Israel | 158389 (138876 to 181862) | 3358.36 (2943.72 to 3856.39) |  | 323624 (284826 to 370484) | 3355.05 (2940.53 to 3851.54) | 0.0089 (-0.0222 to 0.04) |
| Italy | 2598062 (2291580 to 2964739) | 3725.76 (3271.33 to 4256.14) |  | 3079086 (2721409 to 3514658) | 3724.5 (3270.21 to 4254.95) | -0.0061 (-0.0241 to 0.0119) |
| Jamaica | 120682 (106562 to 135202) | 5977.65 (5314.63 to 6644.47) |  | 182145 (161651 to 203072) | 5975.47 (5314.32 to 6642.86) | -0.0013 (-0.0014 to -0.0012) |
| Japan | 3690932 (3206781 to 4288353) | 2441.38 (2130.24 to 2820.14) |  | 4533287 (3998733 to 5233053) | 2452.04 (2139.86 to 2831.52) | 0.0053 (-0.0292 to 0.0397) |
| Jordan | 123630 (105986 to 142924) | 4740.38 (4184.75 to 5379.83) |  | 495989 (428943 to 566632) | 4726.49 (4173.73 to 5363.62) | -0.0092 (-0.0114 to -0.0069) |
| Kazakhstan | 629674 (548305 to 717262) | 4175.54 (3665.95 to 4766.95) |  | 791115 (690496 to 906380) | 4172.31 (3662.41 to 4767.22) | -0.0025 (-0.0029 to -0.0021) |
| Kenya | 648342 (560621 to 747463) | 4556.62 (3993.72 to 5179.8) |  | 1743804 (1510519 to 2011711) | 4558.03 (3995.29 to 5183.8) | 0.0011 (0.001 to 0.0012) |
| Kiribati | 1250 (1074 to 1447) | 2174.98 (1900.19 to 2506.01) |  | 2191 (1884 to 2534) | 2177.78 (1902.33 to 2510.64) | 0.0041 (0.0026 to 0.0056) |
| Kuwait | 73588 (61923 to 85496) | 4667.18 (4112.83 to 5300.22) |  | 236431 (199631 to 274571) | 4718.47 (4155.84 to 5354.61) | 0.0376 (0.035 to 0.0402) |
| Kyrgyzstan | 152368 (133036 to 173201) | 4172.32 (3663.23 to 4766.95) |  | 249628 (217442 to 285126) | 4169.01 (3658.41 to 4764.83) | -0.0024 (-0.0028 to -0.0019) |
| Lao People's Democratic Republic | 65102 (56225 to 75118) | 2172.98 (1898.17 to 2504.84) |  | 139322 (120089 to 160954) | 2169.43 (1895.41 to 2499.71) | -0.0055 (-0.0058 to -0.0052) |
| Latvia | 135941 (119559 to 154182) | 4431.52 (3891.24 to 5040.71) |  | 112004 (99073 to 126980) | 4421.48 (3884.31 to 5029.6) | -0.0075 (-0.008 to -0.007) |
| Lebanon | 130452 (114467 to 147902) | 4767.31 (4204.9 to 5402.43) |  | 257052 (225137 to 291673) | 4777.96 (4222.24 to 5412.88) | 0.0068 (0.0044 to 0.0092) |
| Lesotho | 58178 (50866 to 66879) | 4391.78 (3854.26 to 5012.33) |  | 80941 (70433 to 93193) | 4390.83 (3857.92 to 5011.65) | -0.0007 (-0.001 to -0.0004) |
| Liberia | 60738 (53254 to 69306) | 4385.33 (3846.18 to 5008.62) |  | 160465 (138509 to 185277) | 4381.95 (3845.32 to 5001.95) | -0.0028 (-0.0039 to -0.0018) |
| Libya | 141726 (122036 to 162222) | 4719.47 (4166.72 to 5351.19) |  | 343117 (297198 to 392855) | 4746.72 (4190.69 to 5386.31) | 0.0199 (0.0193 to 0.0205) |
| Lithuania | 187743 (164947 to 212096) | 4591.62 (4018.35 to 5198.28) |  | 169119 (149635 to 191206) | 4582.48 (4028.19 to 5206.13) | -0.0068 (-0.0078 to -0.0058) |
| Luxembourg | 14839 (13017 to 16975) | 3196.78 (2814.03 to 3657.62) |  | 25008 (21982 to 28708) | 3190.53 (2804.66 to 3652.84) | -0.0066 (-0.0072 to -0.0059) |
| Madagascar | 349683 (303885 to 402685) | 4383.92 (3849.14 to 5004.95) |  | 857500 (742472 to 992722) | 4385.48 (3849.75 to 5005.07) | 0.0012 (0.0012 to 0.0013) |
| Malawi | 277223 (240790 to 320083) | 4388.66 (3853.31 to 5009.28) |  | 560933 (486558 to 646137) | 4391.59 (3854.88 to 5012.62) | 0.0026 (0.0021 to 0.0031) |
| Malaysia | 310192 (266703 to 358371) | 2168.93 (1895.33 to 2498.58) |  | 696235 (602374 to 803771) | 2165.35 (1891.98 to 2493.28) | -0.0058 (-0.0061 to -0.0056) |
| Maldives | 3177 (2731 to 3674) | 2157.58 (1883.65 to 2486.04) |  | 11237 (9543 to 13186) | 2148.35 (1876.45 to 2476.52) | -0.0155 (-0.0173 to -0.0137) |
| Mali | 255467 (222565 to 293822) | 4389.27 (3850.52 to 5010.76) |  | 619731 (538014 to 715158) | 4385.2 (3847.89 to 5005.43) | -0.0032 (-0.0036 to -0.0029) |
| Malta | 13056 (11451 to 15032) | 3199.11 (2816.76 to 3662.23) |  | 18688 (16489 to 21510) | 3190.95 (2805.07 to 3653.52) | -0.0089 (-0.0092 to -0.0085) |
| Marshall Islands | 637 (546 to 739) | 2166.6 (1893.71 to 2493.96) |  | 1100 (947 to 1276) | 2165.25 (1891.76 to 2494.21) | -0.0028 (-0.0037 to -0.0019) |
| Mauritania | 62541 (54523 to 71898) | 4388.12 (3851.02 to 5008.42) |  | 132861 (115747 to 152983) | 4387.01 (3850.53 to 5007.56) | -0.0009 (-0.001 to -0.0008) |
| Mauritius | 22115 (19018 to 25540) | 2171.97 (1897.49 to 2502.06) |  | 33617 (29338 to 39033) | 2170.94 (1896.9 to 2500.62) | -0.0016 (-0.0018 to -0.0014) |
| Mexico | 4078356 (3576995 to 4585804) | 6101.58 (5447.12 to 6746.59) |  | 7869414 (7006914 to 8724821) | 6101 (5446.55 to 6744.55) | -0.0003 (-0.0004 to -0.0002) |
| Micronesia (Federated States of) | 1572 (1356 to 1817) | 2166.53 (1892.42 to 2493.12) |  | 2022 (1744 to 2340) | 2169.43 (1895.43 to 2497.94) | 0.0045 (0.0041 to 0.0048) |
| Monaco | 1348 (1189 to 1547) | 3199.39 (2816.29 to 3662.85) |  | 1670 (1464 to 1919) | 3196.24 (2813.3 to 3660.93) | -0.0031 (-0.0038 to -0.0024) |
| Mongolia | 64839 (56026 to 74095) | 4162.97 (3656.04 to 4762.93) |  | 136988 (118009 to 157526) | 4169.48 (3658.73 to 4765.74) | 0.0055 (0.0053 to 0.0057) |
| Montenegro | 25214 (22066 to 28989) | 3896.24 (3407.53 to 4468.98) |  | 30068 (26524 to 34426) | 3894.31 (3404.86 to 4467.58) | -0.0018 (-0.002 to -0.0016) |
| Morocco | 962752 (831967 to 1098107) | 4764.92 (4205.49 to 5402.1) |  | 1747470 (1532666 to 1992030) | 4760.38 (4201.23 to 5397.62) | -0.0031 (-0.0036 to -0.0025) |
| Mozambique | 390246 (340116 to 448248) | 4394.13 (3853.65 to 5015.36) |  | 844026 (731167 to 976212) | 4394.53 (3855.43 to 5015.65) | 0 (-0.0011 to 0.0012) |
| Myanmar | 713159 (615834 to 820881) | 2172.32 (1897.75 to 2503.69) |  | 1181207 (1023001 to 1368057) | 2177.1 (1901.32 to 2510.65) | 0.0077 (0.0075 to 0.0078) |
| Namibia | 44492 (38787 to 51094) | 4390.12 (3854.85 to 5011.11) |  | 88859 (77208 to 102231) | 4392.7 (3858.32 to 5013.48) | 0.0021 (0.0018 to 0.0024) |
| Nauru | 159 (136 to 185) | 2163.96 (1890.74 to 2491.64) |  | 182 (155 to 212) | 2173.99 (1899.31 to 2504.72) | 0.0161 (0.0144 to 0.0179) |
| Nepal | 713805 (623380 to 809516) | 5064.51 (4482.71 to 5713.66) |  | 1412748 (1241840 to 1599591) | 5076.8 (4491.22 to 5720.77) | 0.0083 (0.0079 to 0.0087) |
| Netherlands | 432632 (379179 to 500706) | 2469.33 (2165.15 to 2851.09) |  | 549899 (483871 to 635726) | 2467.3 (2162.46 to 2849.71) | -0.0029 (-0.0099 to 0.0042) |
| New Zealand | 141098 (123932 to 161472) | 3835.31 (3373.27 to 4409.45) |  | 208344 (184419 to 238710) | 3837.03 (3372.85 to 4409.35) | 0.0049 (-0.001 to 0.0109) |
| Nicaragua | 156883 (136606 to 178010) | 5980.58 (5316.39 to 6644.67) |  | 363976 (319935 to 407152) | 5976.39 (5311.71 to 6638.89) | -0.0024 (-0.0025 to -0.0024) |
| Niger | 214790 (186226 to 248118) | 4383.83 (3845.26 to 5004.99) |  | 594567 (515900 to 684536) | 4387.03 (3851.19 to 5007.22) | 0.0027 (0.0025 to 0.0029) |
| Nigeria | 2896084 (2512109 to 3313451) | 4545.79 (3981.86 to 5169.58) |  | 6775177 (5866886 to 7799951) | 4563.29 (4000.82 to 5187.06) | 0.0135 (0.0131 to 0.014) |
| Niue | 45 (40 to 52) | 2171.34 (1896.47 to 2500.52) |  | 40 (35 to 46) | 2169.77 (1895.97 to 2498.71) | -0.0027 (-0.0033 to -0.0022) |
| North Macedonia | 79810 (69651 to 91703) | 3893.94 (3404.55 to 4465.04) |  | 106007 (93036 to 121694) | 3891.24 (3403.2 to 4463.64) | -0.0024 (-0.0027 to -0.0021) |
| Northern Mariana Islands | 906 (764 to 1067) | 2151.66 (1877.97 to 2477.35) |  | 1084 (939 to 1274) | 2163.56 (1889.93 to 2491.9) | 0.0205 (0.0153 to 0.0258) |
| Norway | 107417 (94552 to 123186) | 2121.75 (1850.54 to 2439.49) |  | 145146 (126829 to 169036) | 2148.18 (1872.32 to 2496.12) | 0.0436 (0.0429 to 0.0444) |
| Oman | 67727 (57688 to 78051) | 4667.51 (4124.35 to 5302.79) |  | 222305 (186510 to 259373) | 4643.82 (4103.37 to 5278.75) | -0.0174 (-0.0493 to 0.0146) |
| Pakistan | 4090022 (3579789 to 4658440) | 5223.63 (4638.21 to 5898.19) |  | 9029499 (7864038 to 10305955) | 5229.41 (4642.48 to 5904.41) | 0.0039 (0.0038 to 0.0039) |
| Palau | 301 (259 to 348) | 2166.69 (1893.2 to 2495.95) |  | 461 (398 to 540) | 2159.22 (1885.78 to 2486.79) | -0.0164 (-0.025 to -0.0077) |
| Palestine | 64843 (55828 to 74426) | 4778.94 (4224.04 to 5414.58) |  | 191464 (164821 to 219569) | 4756.92 (4198.92 to 5394.88) | -0.0157 (-0.0162 to -0.0152) |
| Panama | 122677 (107601 to 137774) | 5972.32 (5314.16 to 6642.42) |  | 251492 (223803 to 279538) | 5972.34 (5312.65 to 6640.49) | 0 (-0.0001 to 0.0002) |
| Papua New Guinea | 66126 (56689 to 76606) | 2164.11 (1891.37 to 2492.55) |  | 172425 (147893 to 200200) | 2163.51 (1890.75 to 2493.68) | -0.0011 (-0.0014 to -0.0008) |
| Paraguay | 190685 (166281 to 213559) | 6070.47 (5388.74 to 6729.56) |  | 411657 (361675 to 459755) | 6069.06 (5387.11 to 6728.66) | -0.0008 (-0.0009 to -0.0008) |
| Peru | 1041260 (912431 to 1171522) | 5975.91 (5315.09 to 6645.11) |  | 2067980 (1829172 to 2304682) | 5974.1 (5312.59 to 6640.55) | -0.0013 (-0.0015 to -0.001) |
| Philippines | 1072234 (919288 to 1246867) | 2249.51 (1954.51 to 2592.35) |  | 2287656 (1973969 to 2661871) | 2249.76 (1953.96 to 2592.8) | 0.0005 (0.0003 to 0.0007) |
| Poland | 2111330 (1867220 to 2368322) | 5132.06 (4543.64 to 5787.23) |  | 2559565 (2276252 to 2857182) | 5128.38 (4540.05 to 5782.8) | -0.0026 (-0.0029 to -0.0024) |
| Portugal | 392629 (343969 to 452034) | 3379.53 (2949.89 to 3887.1) |  | 488094 (428889 to 563233) | 3377.95 (2947.14 to 3884.48) | -0.0022 (-0.0051 to 0.0008) |
| Puerto Rico | 217001 (192779 to 241289) | 5980.14 (5315.23 to 6642.27) |  | 264283 (238882 to 291186) | 5978.35 (5313.22 to 6640.16) | -0.001 (-0.0011 to -0.0008) |
| Qatar | 19658 (16339 to 23098) | 4600.12 (4058.06 to 5231.51) |  | 152798 (127379 to 179775) | 4552.99 (4008.35 to 5179.05) | -0.037 (-0.0397 to -0.0342) |
| Republic of Korea | 1197944 (1030385 to 1384711) | 2801.86 (2455.35 to 3241.54) |  | 2009932 (1761911 to 2339295) | 2797.25 (2451.56 to 3239.66) | 0.0906 (-0.1464 to 0.3281) |
| Republic of Moldova | 200876 (174731 to 228725) | 4428.24 (3886.75 to 5036.35) |  | 207888 (182504 to 237043) | 4419.87 (3881.12 to 5028.36) | -0.0065 (-0.0069 to -0.0061) |
| Romania | 997821 (877348 to 1143041) | 3895.55 (3407.09 to 4468.58) |  | 988930 (873475 to 1134323) | 3894.33 (3406.29 to 4468.68) | -0.001 (-0.0012 to -0.0007) |
| Russian Federation | 7499711 (6590086 to 8536505) | 4447.45 (3909.14 to 5054.19) |  | 8185591 (7240578 to 9322319) | 4443.44 (3906.61 to 5057.64) | 0.0369 (-0.0941 to 0.1681) |
| Rwanda | 205110 (177755 to 235826) | 4391.8 (3857.67 to 5013.36) |  | 432381 (374730 to 498097) | 4393.23 (3858.33 to 5013.47) | 0.0017 (-0.0024 to 0.0057) |
| Saint Kitts and Nevis | 2186 (1933 to 2438) | 5975.89 (5310.52 to 6639.03) |  | 4083 (3637 to 4544) | 5973.68 (5313.64 to 6641.96) | -0.0012 (-0.0013 to -0.0011) |
| Saint Lucia | 6633 (5840 to 7452) | 5978.85 (5312.94 to 6640.73) |  | 12199 (10873 to 13554) | 5973.83 (5312.73 to 6642.47) | -0.003 (-0.003 to -0.0029) |
| Saint Vincent and the Grenadines | 5315 (4679 to 5969) | 5973.43 (5310.29 to 6638.2) |  | 7484 (6695 to 8309) | 5971.15 (5314.01 to 6641.51) | -0.0012 (-0.0014 to -0.001) |
| Samoa | 2672 (2317 to 3078) | 2166.39 (1892.73 to 2496.15) |  | 3931 (3408 to 4523) | 2166.3 (1893.48 to 2494.21) | -0.0002 (-0.0005 to 0.0002) |
| San Marino | 875 (771 to 1005) | 3193.61 (2808.28 to 3655.19) |  | 1353 (1194 to 1554) | 3201.18 (2818.89 to 3667.35) | 0.0081 (0.008 to 0.0083) |
| Sao Tome and Principe | 3566 (3122 to 4072) | 4388.92 (3852.75 to 5008.22) |  | 7304 (6328 to 8421) | 4383.72 (3849.21 to 5003.76) | -0.0043 (-0.0045 to -0.004) |
| Saudi Arabia | 561628 (479012 to 646755) | 4680.37 (4129.1 to 5311.57) |  | 1827651 (1551267 to 2115740) | 4683.42 (4130.31 to 5313.92) | 0.0019 (0.0006 to 0.0032) |
| Senegal | 216426 (188634 to 249455) | 4387.49 (3849.34 to 5008.74) |  | 497163 (432560 to 572675) | 4387.16 (3851.8 to 5007.26) | -0.0003 (-0.0005 to -0.0002) |
| Serbia | 413040 (363111 to 475403) | 3894.75 (3404.96 to 4467.47) |  | 437125 (385444 to 501626) | 3893.62 (3404.47 to 4466.44) | -0.001 (-0.0012 to -0.0007) |
| Seychelles | 1415 (1231 to 1621) | 2171.19 (1897.1 to 2499.8) |  | 2481 (2153 to 2886) | 2163.42 (1890.17 to 2490.89) | -0.0122 (-0.013 to -0.0113) |
| Sierra Leone | 115661 (100724 to 132766) | 4386.54 (3849.19 to 5008.24) |  | 267468 (231224 to 309014) | 4384.24 (3847.04 to 5004.14) | -0.0019 (-0.0027 to -0.0012) |
| Singapore | 93280 (80159 to 108172) | 2943.68 (2570.47 to 3366.67) |  | 217937 (190270 to 252257) | 2958.38 (2594.24 to 3397.14) | 0.0159 (0.004 to 0.0278) |
| Slovakia | 219913 (192133 to 251011) | 3897.71 (3408.22 to 4470.39) |  | 275720 (242365 to 316051) | 3894.65 (3406.53 to 4468.72) | -0.0027 (-0.0029 to -0.0026) |
| Slovenia | 87874 (77010 to 100707) | 3897.99 (3411.71 to 4471.65) |  | 108764 (96259 to 125102) | 3891.64 (3403.79 to 4464.47) | -0.0056 (-0.0058 to -0.0055) |
| Solomon Islands | 4928 (4231 to 5701) | 2159.99 (1886.14 to 2488.78) |  | 11049 (9488 to 12826) | 2166.86 (1893.43 to 2497.19) | 0.011 (0.0109 to 0.0112) |
| Somalia | 200637 (173906 to 231234) | 4386.67 (3853.32 to 5006.64) |  | 572217 (494273 to 666424) | 4388.47 (3856.59 to 5008.63) | 0.0013 (0.0004 to 0.0022) |
| South Africa | 1357919 (1181025 to 1551168) | 4563.87 (4001.04 to 5192.76) |  | 2528274 (2192497 to 2890894) | 4561.36 (3998.74 to 5189.6) | -0.0019 (-0.0024 to -0.0015) |
| South Sudan | 172551 (149373 to 198782) | 4371.43 (3835.18 to 4987.55) |  | 276952 (240922 to 317510) | 4386.25 (3847.68 to 5005.94) | 0.0125 (0.0112 to 0.0138) |
| Spain | 1314984 (1149711 to 1513710) | 2944.89 (2562.72 to 3404.1) |  | 1804017 (1580863 to 2091042) | 2941.01 (2559.04 to 3399.83) | 0.0733 (-0.0945 to 0.2415) |
| Sri Lanka | 331066 (285437 to 382089) | 2167.71 (1894.07 to 2498.39) |  | 523171 (456888 to 605609) | 2174.69 (1899.65 to 2506.79) | 0.0111 (0.0108 to 0.0113) |
| Sudan | 681916 (588056 to 777562) | 4758.77 (4194.62 to 5395.84) |  | 1535322 (1321037 to 1759247) | 4753.35 (4189.23 to 5389.46) | -0.0039 (-0.0043 to -0.0035) |
| Suriname | 20265 (17828 to 22755) | 5972.36 (5311.66 to 6638.83) |  | 36227 (32285 to 40299) | 5975.94 (5312.85 to 6643.75) | 0.0022 (0.0019 to 0.0024) |
| Sweden | 295078 (258255 to 338251) | 2798.17 (2438.21 to 3223.4) |  | 383784 (338496 to 442127) | 2947.12 (2564.19 to 3405.32) | 0.1777 (0.15 to 0.2054) |
| Switzerland | 177392 (155824 to 204122) | 2110.4 (1853.84 to 2437.52) |  | 243921 (215840 to 280254) | 2106.58 (1850.1 to 2435.22) | 0.0425 (-0.0402 to 0.1253) |
| Syrian Arab Republic | 410050 (353044 to 471022) | 4749.27 (4189.68 to 5388.94) |  | 671987 (590714 to 769532) | 4778.11 (4210.21 to 5414.37) | 0.0208 (0.0184 to 0.0233) |
| Taiwan (Province of China) | 408762 (349516 to 468897) | 2024.02 (1748.7 to 2316.06) |  | 638986 (556558 to 743773) | 2077.02 (1809.67 to 2394.84) | 0.0627 (-0.0103 to 0.1357) |
| Tajikistan | 159787 (138394 to 181888) | 4164.83 (3657.15 to 4763.35) |  | 337420 (291706 to 386135) | 4161.98 (3656.66 to 4761.54) | -0.0021 (-0.0025 to -0.0017) |
| Thailand | 1127675 (971262 to 1300487) | 2172.31 (1897.68 to 2503.89) |  | 1913935 (1669877 to 2234065) | 2172.37 (1897.65 to 2503.57) | 0 (-0.0006 to 0.0006) |
| Timor-Leste | 12199 (10428 to 14204) | 2165.38 (1892.38 to 2495.15) |  | 23122 (20071 to 26668) | 2167.66 (1894.74 to 2497.61) | 0.0043 (0.0031 to 0.0056) |
| Togo | 100289 (86743 to 115980) | 4392.44 (3854.67 to 5012.84) |  | 266871 (230895 to 308074) | 4394.47 (3857.48 to 5013.79) | 0.0018 (0.0015 to 0.002) |
| Tokelau | 29 (26 to 33) | 2176 (1900.04 to 2509.22) |  | 29 (25 to 33) | 2165.86 (1892.3 to 2495.68) | -0.0149 (-0.0266 to -0.0032) |
| Tonga | 1588 (1381 to 1832) | 2171.53 (1896.55 to 2504.63) |  | 1938 (1689 to 2232) | 2172.41 (1898 to 2505.55) | 0.0013 (0.0011 to 0.0015) |
| Trinidad and Tobago | 64486 (56694 to 72221) | 5973.68 (5312.27 to 6641.6) |  | 96969 (86430 to 107280) | 5972.06 (5311.97 to 6639.71) | -0.0009 (-0.001 to -0.0009) |
| Tunisia | 328208 (284737 to 374213) | 4756.96 (4197.06 to 5395.48) |  | 608257 (533157 to 692315) | 4765.92 (4205.95 to 5403.48) | 0.0066 (0.0064 to 0.0067) |
| Turkey | 2545364 (2209759 to 2904147) | 5057.71 (4454.92 to 5709.69) |  | 4851733 (4315371 to 5427680) | 5248.27 (4687.28 to 5842.6) | 0.1151 (0.09 to 0.1403) |
| Turkmenistan | 115295 (99696 to 131668) | 4170.14 (3661.66 to 4765.7) |  | 203336 (176842 to 233148) | 4163.57 (3658.07 to 4758.4) | -0.0055 (-0.0057 to -0.0053) |
| Tuvalu | 178 (154 to 206) | 2180.58 (1904.17 to 2514.44) |  | 247 (215 to 284) | 2167.53 (1893.83 to 2495.51) | -0.0209 (-0.0211 to -0.0206) |
| Uganda | 471063 (407602 to 541635) | 4387.14 (3850.54 to 5007.85) |  | 1157109 (999038 to 1336883) | 4393.1 (3857.1 to 5013.54) | 0.0047 (0.0045 to 0.0049) |
| Ukraine | 2810860 (2481853 to 3181436) | 4612.41 (4054.26 to 5226.03) |  | 2650188 (2336870 to 2992678) | 4608.28 (4050.17 to 5221.74) | -0.0032 (-0.0038 to -0.0025) |
| United Arab Emirates | 78225 (65016 to 92178) | 4609.02 (4064.42 to 5242.05) |  | 527547 (434567 to 629823) | 4586.93 (4035.18 to 5218.52) | -0.0145 (-0.025 to -0.0041) |
| United Kingdom | 2755951 (2429780 to 3129803) | 4026.73 (3545.66 to 4583.27) |  | 3375015 (2982218 to 3839513) | 4005.9 (3509.66 to 4579.98) | 0.0468 (-0.1203 to 0.2142) |
| United Republic of Tanzania | 742470 (646128 to 853577) | 4390.13 (3852.06 to 5012.04) |  | 1758855 (1528018 to 2030870) | 4390.11 (3851.68 to 5010.98) | -0.0001 (-0.0003 to 0) |
| United States of America | 11974196 (10383074 to 13594483) | 4216 (3666.27 to 4788.3) |  | 15341054 (13550603 to 17501632) | 3810.93 (3325.21 to 4379.04) | -0.3912 (-0.4719 to -0.3105) |
| United States Virgin Islands | 6164 (5448 to 6874) | 5981.71 (5316.27 to 6644.32) |  | 7454 (6701 to 8225) | 5981.26 (5314.65 to 6643.68) | 0.0001 (-0.0003 to 0.0004) |
| Uruguay | 165675 (146751 to 187252) | 5003.18 (4406.71 to 5687.51) |  | 199272 (177141 to 224700) | 5003.96 (4407.45 to 5688.29) | 0.0006 (0.0005 to 0.0006) |
| Uzbekistan | 651926 (564691 to 743007) | 4168.74 (3660.32 to 4763.96) |  | 1300876 (1127631 to 1494906) | 4167.27 (3657.4 to 4763.65) | -0.0011 (-0.0014 to -0.0009) |
| Vanuatu | 2322 (1994 to 2683) | 2161.3 (1887.65 to 2489.82) |  | 5218 (4499 to 6021) | 2165.6 (1891.8 to 2495.65) | 0.0067 (0.0064 to 0.007) |
| Venezuela (Bolivarian Republic of) | 917683 (801637 to 1035016) | 5975.6 (5313.56 to 6643.72) |  | 1780002 (1580068 to 1981665) | 5978.01 (5314.78 to 6644.69) | 0.0017 (0.0012 to 0.0023) |
| Viet Nam | 1172635 (1009734 to 1349463) | 2178.79 (1902.53 to 2512.29) |  | 2289292 (1980485 to 2658065) | 2173.79 (1899.16 to 2503.79) | -0.0079 (-0.0081 to -0.0077) |
| Yemen | 401112 (345640 to 459345) | 4762.14 (4200.31 to 5400.78) |  | 1141827 (981907 to 1311933) | 4760.94 (4202.96 to 5400.36) | -0.001 (-0.0015 to -0.0006) |
| Zambia | 218813 (189314 to 252479) | 4384.54 (3848.13 to 5006.26) |  | 561305 (483920 to 649563) | 4385.96 (3850.75 to 5006.52) | 0.0012 (0.0011 to 0.0013) |
| Zimbabwe | 293414 (254837 to 337281) | 4388.85 (3849.22 to 5008.99) |  | 498291 (432239 to 574055) | 4396.02 (3861.56 to 5017.81) | 0.0058 (0.0055 to 0.0062) |

UI: uncertainty interval, CI: confidence interval, AAPC, average annual percent change.
