# Supplementary material for: Burden of gastroesophageal reflux disease in 204 countries and territories, 1990–2019: a systematic analysis for the Global Burden of disease study 2019
Source: BMC Public Health. 2023 Mar 29;23:582. doi: 10.1186/s12889-023-15272-z (PMC10053627; doi:10.1186/s12889-023-15272-z)
Supplement: Supplementary file 3 — Table S2. Prevalence of gastro-oesophageal reflux disease in 1990 and 2019 for both sexes and all locations, with AAPC from 1990 and 2019. [file 12889_2023_15272_MOESM3_ESM.docx]

Table S2. Prevalence of gastro-oesophageal reflux disease in 1990 and 2019 for both sexes and all locations, with AAPC from 1990 and 2019.

| location | 1990 | |  | 2019 | | AAPC % (95% CI)  1990-2019 |
| --- | --- | --- | --- | --- | --- | --- |
|  | Cases (95% UI) | Age-standardised YLDs per  100 000 population (95% UI) |  | Cases (95% UI) | Age-standardised YLDs per 100 000 population (95% UI) |  |
| Global | 441574363 (383480879 to 496840028) | 9344.52 (8213.69 to 10456.84) |  | 783945161 (689550733 to 876534233) | 9574.45 (8416.42 to 10698.38) | 0.0959 (0.0693 to 0.1225) |
| **Sex** |  |  |  |  |  |  |
| Female | 232217037 (202644666 to 261401141) | 9719.69 (8537.36 to 10915.06) |  | 412645692 (362642384 to 463196184) | 9941.9 (8717.74 to 11155.47) | 0.0865 (0.0586 to 0.1144) |
| Male | 209357326 (180883033 to 236527916) | 8965.06 (7872.25 to 10037.6) |  | 371299469 (325065564 to 416959488) | 9202.85 (8087.69 to 10290.43) | 0.1054 (0.0694 to 0.1414) |
| **SDI** |  |  |  |  |  |  |
| High SDI | 80129581 (70492518 to 90046784) | 8512.56 (7442.29 to 9594.13) |  | 108963554 (96339328 to 123535247) | 8252.43 (7193.12 to 9291.2) | -0.1018 (-0.1402 to -0.0633) |
| High-middle SDI | 104258233 (91355482 to 117108757) | 9014.77 (7927.55 to 10110.23) |  | 155329203 (137887005 to 173542022) | 8700.91 (7677.19 to 9701.44) | -0.0995 (-0.1452 to -0.0537) |
| Middle SDI | 116152841 (100500968 to 131537450) | 8082.43 (7099.76 to 9064.77) |  | 228622408 (199772197 to 257258463) | 8720.16 (7652.32 to 9769.9) | 0.2963 (0.2606 to 0.3321) |
| Low-middle SDI | 98861524 (86013501 to 111479239) | 11773.99 (10401.16 to 13164.3) |  | 195611491 (171095393 to 219430480) | 11887.87 (10505.45 to 13282.26) | 0.0477 (0.022 to 0.0735) |
| Low SDI | 41902732 (36208673 to 47368554) | 12085.16 (10597.1 to 13536.65) |  | 94929363 (81954829 to 107279120) | 12054.64 (10577.33 to 13501.86) | -0.0098 (-0.0153 to -0.0042) |
| **Region** |  |  |  |  |  |  |
| High-income Asia Pacific | 12104351 (10586660 to 13847459) | 6119.1 (5333.23 to 6997.03) |  | 16884767 (14885589 to 19363357) | 6225.69 (5431.16 to 7113.27) | 0.1037 (0.028 to 0.1795) |
| High-income North America | 33730400 (29427491 to 38105207) | 10614.3 (9254.75 to 11985.62) |  | 43136676 (38017272 to 48933947) | 9401.6 (8190.07 to 10614.97) | -0.462 (-0.5585 to -0.3655) |
| Western Europe | 38312284 (33873905 to 43118747) | 8218.38 (7188.66 to 9247.73) |  | 48043927 (42555669 to 54147516) | 8217.57 (7182.45 to 9250.17) | 0.0098 (-0.0127 to 0.0324) |
| Australasia | 1937387 (1693605 to 2201797) | 8694.45 (7579.92 to 9896.39) |  | 3130260 (2769824 to 3551039) | 8679.81 (7566.16 to 9881.92) | -0.0022 (-0.1335 to 0.1292) |
| Andean Latin America | 4639874 (4055224 to 5225552) | 15934.38 (14135.36 to 17780.23) |  | 9934970 (8774283 to 11099233) | 15932.06 (14135.41 to 17774.71) | -0.0007 (-0.0009 to -0.0005) |
| Tropical Latin America | 20853832 (18269389 to 23294497) | 16335.09 (14509.64 to 18040.17) |  | 40245627 (35511521 to 44588035) | 16207.5 (14319.22 to 17944.65) | -0.0537 (-0.1112 to 0.0038) |
| Central Latin America | 19850194 (17256564 to 22247938) | 15940.7 (14148 to 17600.1) |  | 40724057 (36024577 to 45037586) | 15949.53 (14156.95 to 17601.72) | 0.002 (0.0019 to 0.0022) |
| Southern Latin America | 6488805 (5641310 to 7308025) | 13628.01 (11862.59 to 15357.49) |  | 10204386 (8924162 to 11472586) | 13625.51 (11860.68 to 15353.48) | -0.0423 (-0.1101 to 0.0257) |
| Caribbean | 4960970 (4365282 to 5556878) | 15937.21 (14138.44 to 17780.66) |  | 8001360 (7113300 to 8920132) | 15935.32 (14137.14 to 17777.84) | -0.0004 (-0.0005 to -0.0003) |
| Central Europe | 14863126 (13071525 to 16779346) | 10863.54 (9520.36 to 12246.17) |  | 16918847 (15047428 to 19041022) | 10987.32 (9631.12 to 12376.53) | 0.0392 (0.0373 to 0.041) |
| Eastern Europe | 29286216 (25878125 to 32854853) | 11356.33 (9995.84 to 12756.35) |  | 31008844 (27494156 to 34804676) | 11331.34 (9974.89 to 12728.49) | 0.0275 (-0.0843 to 0.1395) |
| Central Asia | 5980338 (5166428 to 6772665) | 10482.56 (9135.45 to 11801.76) |  | 9568453 (8202434 to 10836308) | 10471.5 (9125.15 to 11787.88) | -0.0034 (-0.0038 to -0.0029) |
| North Africa and Middle East | 30903227 (26652912 to 35130358) | 12240.02 (10688.33 to 13786.42) |  | 72251838 (63102947 to 80947469) | 12341.45 (10898.84 to 13766.4) | 0.0166 (-0.0103 to 0.0436) |
| South Asia | 109492566 (95107235 to 123796718) | 13377.44 (11777.46 to 14977.74) |  | 227493099 (198749134 to 256183186) | 13377.84 (11769.1 to 14984.49) | -0.0005 (-0.0232 to 0.0222) |
| Southeast Asia | 20032216 (17125909 to 23042896) | 5432.59 (4716.44 to 6211.09) |  | 37884332 (32476039 to 43387628) | 5427.51 (4711.75 to 6203.74) | -0.0033 (-0.0035 to -0.0031) |
| East Asia | 52254125 (44678263 to 60165723) | 4542.27 (3937 to 5164.94) |  | 84692834 (73460206 to 96979616) | 4523.4 (3912.03 to 5149.59) | 0.1153 (-0.0764 to 0.3074) |
| Oceania | 250166 (212303 to 289356) | 5283.72 (4563.88 to 6047.55) |  | 568997 (483274 to 659313) | 5284.59 (4563.2 to 6048.44) | 0.0004 (0.0002 to 0.0006) |
| Western Sub-Saharan Africa | 14214179 (12205423 to 16096956) | 11224.51 (9779.71 to 12643.62) |  | 34382064 (29505102 to 38936097) | 11236.58 (9782.82 to 12626.62) | 0.0039 (0.0036 to 0.0041) |
| Eastern Sub-Saharan Africa | 13101295 (11224877 to 14858628) | 11191.12 (9742 to 12561.77) |  | 30799118 (26347324 to 35017174) | 11199.13 (9750.44 to 12572.49) | 0.0025 (0.0024 to 0.0027) |
| Central Sub-Saharan Africa | 3906319 (3333613 to 4457041) | 11036.02 (9606.3 to 12450.75) |  | 9802005 (8357525 to 11178502) | 11035.93 (9621.69 to 12451.49) | -0.0001 (-0.0001 to 0) |
| Southern Sub-Saharan Africa | 4412492 (3777350 to 5002431) | 11369.36 (9899.33 to 12811.4) |  | 8268701 (7098563 to 9350800) | 11379.36 (9906.25 to 12826.2) | 0.0034 (0.0024 to 0.0044) |

UI: uncertainty interval, CI: confidence interval, AAPC: average annual percent change, YLDs: Years Lived with Disability.
