# Supplementary material for: Burden of gastroesophageal reflux disease in 204 countries and territories, 1990–2019: a systematic analysis for the Global Burden of disease study 2019
Source: BMC Public Health. 2023 Mar 29;23:582. doi: 10.1186/s12889-023-15272-z (PMC10053627; doi:10.1186/s12889-023-15272-z)
Supplement: Supplementary file 4 — Table S3. Prevalence of gastro-oesophageal reflux disease in 1990 and 2019 with AAPC from 2009 and 2019 at countries/territories level, both sexs. [file 12889_2023_15272_MOESM4_ESM.docx]

Table S3. Prevalence of gastro-oesophageal reflux disease in 1990 and 2019 with AAPC from 2009 and 2019 at countries/territories level, both sexs.

| Countries/territories | 1990 | |  | 2019 | | AAPC % (95% CI)  1990-2019 |
| --- | --- | --- | --- | --- | --- | --- |
|  | Cases (95% UI) | Age-standardised prevalence per  100 000 population (95% UI) |  | Cases (95% UI) | Age-standardised prevalence per  100 000 population (95% UI) |  |
| Afghanistan | 978152 (846627 to 1108765) | 12188.41 (10593.17 to 13761.34) |  | 3044987 (2585243 to 3508217) | 12136.52 (10560.51 to 13698.99) | -0.0142 (-0.0158 to -0.0127) |
| Albania | 182107 (156545 to 209399) | 6560.35 (5673.2 to 7521.67) |  | 222941 (194943 to 254334) | 6557.53 (5673.7 to 7510.29) | -0.0248 (-0.0561 to 0.0064) |
| Algeria | 2188852 (1887895 to 2502979) | 12139.21 (10555.12 to 13701.23) |  | 5092797 (4387057 to 5783724) | 12126.75 (10542.52 to 13691.37) | -0.0037 (-0.0039 to -0.0034) |
| American Samoa | 1933 (1640 to 2237) | 5285.56 (4566.34 to 6052.05) |  | 2755 (2363 to 3154) | 5296.57 (4569.92 to 6057.75) | 0.0072 (0.0068 to 0.0076) |
| Andorra | 4872 (4216 to 5560) | 7762.63 (6746.7 to 8819.98) |  | 8894 (7772 to 10214) | 7779.31 (6763.08 to 8846.91) | 0.0077 (-0.0013 to 0.0166) |
| Angola | 729565 (620608 to 834601) | 11017.24 (9604.43 to 12434.98) |  | 2128463 (1815030 to 2429678) | 11048.66 (9623.91 to 12465.06) | 0.0099 (0.0097 to 0.0101) |
| Antigua and Barbuda | 8949 (7896 to 10005) | 15951.8 (14147.63 to 17785.43) |  | 16431 (14559 to 18342) | 15942.05 (14143.14 to 17778.62) | -0.002 (-0.0023 to -0.0018) |
| Argentina | 4362874 (3801650 to 4913047) | 13626.98 (11861.15 to 15356.24) |  | 6729433 (5882781 to 7569746) | 13626.28 (11861.68 to 15354.31) | -0.0651 (-0.1679 to 0.0378) |
| Armenia | 334636 (287956 to 379276) | 10476.46 (9127.99 to 11794.68) |  | 382422 (334627 to 430981) | 10477.15 (9128.81 to 11792.08) | 0.0005 (0.0001 to 0.0008) |
| Australia | 1582009 (1382163 to 1799361) | 8508.9 (7412.5 to 9705.51) |  | 2596210 (2293197 to 2952857) | 8509.09 (7408.69 to 9708.47) | 0.0069 (-0.1485 to 0.1626) |
| Austria | 970811 (842281 to 1108234) | 10222.09 (8754.5 to 11692.28) |  | 1227960 (1069511 to 1404636) | 10190.2 (8720.43 to 11656.46) | -0.0113 (-0.0149 to -0.0077) |
| Azerbaijan | 658629 (568646 to 747162) | 10484.21 (9133.98 to 11804.15) |  | 1181275 (1011639 to 1341384) | 10465.45 (9120.04 to 11781.43) | -0.0059 (-0.0062 to -0.0057) |
| Bahamas | 35957 (31280 to 40456) | 15946.54 (14144.8 to 17781.53) |  | 67272 (59395 to 75205) | 15944.78 (14144.01 to 17781.05) | -0.0004 (-0.0005 to -0.0003) |
| Bahrain | 53352 (45118 to 61984) | 11917.25 (10373.32 to 13462.44) |  | 206155 (175334 to 237296) | 11851.81 (10314.64 to 13403.39) | -0.0212 (-0.0241 to -0.0182) |
| Bangladesh | 9310245 (8008871 to 10538358) | 12946.88 (11362.69 to 14477.06) |  | 20040189 (17412520 to 22475494) | 13044.64 (11445.08 to 14572.4) | 0.0413 (-0.4155 to 0.5002) |
| Barbados | 42142 (37513 to 46924) | 15949.19 (14146.31 to 17782.58) |  | 59749 (53300 to 66464) | 15942.98 (14142.91 to 17779.32) | -0.0013 (-0.0014 to -0.0012) |
| Belarus | 1338427 (1174705 to 1509392) | 11321.18 (9933.92 to 12747.09) |  | 1403585 (1239014 to 1583513) | 11302.83 (9920.3 to 12728.54) | -0.0052 (-0.0058 to -0.0047) |
| Belgium | 1032506 (907548 to 1170680) | 8410.58 (7343.03 to 9479.24) |  | 1274335 (1116016 to 1438081) | 8528.38 (7365.25 to 9673.44) | 0.0487 (0.028 to 0.0693) |
| Belize | 20480 (17931 to 23071) | 15916.38 (14123.69 to 17761.24) |  | 59731 (52279 to 67095) | 15931.73 (14136.86 to 17779.71) | 0.0033 (0.0032 to 0.0035) |
| Benin | 323399 (276734 to 368647) | 11050.89 (9619.23 to 12472.7) |  | 897863 (765421 to 1025116) | 11038.88 (9614.86 to 12451.26) | -0.0037 (-0.0038 to -0.0036) |
| Bermuda | 10843 (9562 to 12141) | 15941.8 (14140.9 to 17777.68) |  | 13866 (12397 to 15356) | 15936.3 (14135.3 to 17781.69) | -0.0012 (-0.0012 to -0.0011) |
| Bhutan | 53915 (46541 to 61211) | 13049.66 (11475.15 to 14583.36) |  | 95433 (83118 to 107738) | 13046.01 (11462.68 to 14582.06) | -0.0018 (-0.0027 to -0.0008) |
| Bolivia (Plurinational State of) | 741996 (647874 to 835554) | 15941.87 (14141.43 to 17783.11) |  | 1728561 (1518463 to 1937429) | 15929.31 (14133.14 to 17775.3) | -0.0027 (-0.0028 to -0.0027) |
| Bosnia and Herzegovina | 454872 (395277 to 518756) | 9663.84 (8458.67 to 10945.14) |  | 431327 (379510 to 491600) | 9657.35 (8453.02 to 10944.31) | -0.0022 (-0.0025 to -0.002) |
| Botswana | 95093 (81262 to 108301) | 11055.76 (9633.76 to 12475.04) |  | 233656 (199664 to 267066) | 11043.57 (9630.31 to 12459.36) | -0.0038 (-0.004 to -0.0036) |
| Brazil | 20358919 (17834680 to 22739149) | 16335.57 (14523.39 to 18037.91) |  | 39154618 (34579542 to 43371836) | 16204.14 (14316.26 to 17944.68) | -0.0551 (-0.114 to 0.0037) |
| Brunei Darussalam | 13083 (11156 to 15239) | 6468.52 (5631.41 to 7333.68) |  | 29630 (25344 to 33965) | 6471.07 (5636.62 to 7337.59) | 0.0017 (0.001 to 0.0024) |
| Bulgaria | 1013827 (888749 to 1162610) | 9656.86 (8453.38 to 10943.9) |  | 942872 (830445 to 1074157) | 9656.75 (8453.43 to 10939.53) | -0.0002 (-0.0004 to 0.0001) |
| Burkina Faso | 652766 (559859 to 740502) | 11057.07 (9622.1 to 12484.48) |  | 1623120 (1386604 to 1851643) | 11052.35 (9622.64 to 12475.18) | -0.0014 (-0.0016 to -0.0013) |
| Burundi | 389631 (333156 to 444956) | 11045.51 (9615.63 to 12462.73) |  | 854153 (726951 to 978050) | 11012.57 (9604.32 to 12429.66) | -0.0106 (-0.0113 to -0.01) |
| Cabo Verde | 26781 (23318 to 30243) | 11077.44 (9620.24 to 12506.57) |  | 59114 (50968 to 67038) | 11025.04 (9618.39 to 12445.61) | -0.0162 (-0.0168 to -0.0157) |
| Cambodia | 366906 (312494 to 424013) | 5325.72 (4599.15 to 6101.88) |  | 814612 (693725 to 938387) | 5316.33 (4591.17 to 6081.24) | -0.0061 (-0.0063 to -0.0059) |
| Cameroon | 737623 (631077 to 838267) | 11034.97 (9612.36 to 12447.98) |  | 2237147 (1901632 to 2563499) | 11029.07 (9612.6 to 12443.81) | -0.0019 (-0.002 to -0.0018) |
| Canada | 2229681 (1936950 to 2545917) | 7165.07 (6232.2 to 8169.35) |  | 3416950 (2990046 to 3916509) | 7162.6 (6229.36 to 8165.56) | 0.0112 (-0.0107 to 0.0331) |
| Central African Republic | 201437 (171727 to 229383) | 11042.97 (9618.54 to 12455.68) |  | 402918 (343980 to 459273) | 11044.91 (9636.99 to 12457.67) | 0.0007 (0.0003 to 0.0011) |
| Chad | 416033 (357504 to 471647) | 11046.4 (9612.58 to 12462.35) |  | 1027113 (875260 to 1170662) | 11019.88 (9596.02 to 12431.93) | -0.0084 (-0.0086 to -0.0082) |
| Chile | 1670660 (1444391 to 1896845) | 13630.22 (11865.29 to 15360.69) |  | 2921560 (2556272 to 3293475) | 13622.28 (11856.44 to 15349.85) | -0.0021 (-0.0022 to -0.0019) |
| China | 50338705 (43013935 to 57975682) | 4532.19 (3927.36 to 5154.28) |  | 81636483 (70733802 to 93513614) | 4509.32 (3899.11 to 5133.17) | 0.1173 (-0.0827 to 0.3178) |
| Colombia | 4212461 (3666196 to 4749555) | 15937.55 (14137.75 to 17783.77) |  | 8253065 (7325038 to 9195712) | 15941.41 (14142.51 to 17777.35) | 0.0007 (0.0005 to 0.0009) |
| Comoros | 33391 (28648 to 37791) | 11033.78 (9608.8 to 12446.19) |  | 68654 (59119 to 77875) | 11028.79 (9614.62 to 12446.71) | -0.0015 (-0.0017 to -0.0014) |
| Congo | 175609 (150018 to 199776) | 11045.56 (9622.78 to 12458.91) |  | 457783 (391717 to 521535) | 11027.97 (9618.88 to 12442.49) | -0.0055 (-0.0058 to -0.0053) |
| Cook Islands | 838 (716 to 964) | 5278.61 (4557.2 to 6042.16) |  | 1083 (944 to 1242) | 5301.52 (4573.92 to 6064.68) | 0.015 (0.0128 to 0.0172) |
| Costa Rica | 392420 (342483 to 441348) | 15931.64 (14133.96 to 17778.12) |  | 829399 (734302 to 925504) | 15946.75 (14145.24 to 17783.44) | 0.0033 (0.0032 to 0.0034) |
| Croatia | 562359 (492088 to 641592) | 9665.62 (8458.09 to 10953.33) |  | 567321 (499525 to 644569) | 9656.43 (8452.11 to 10941.22) | -0.0033 (-0.0033 to -0.0032) |
| Cuba | 1772407 (1565176 to 1977389) | 15926.6 (14132.03 to 17772.93) |  | 2317396 (2070997 to 2580857) | 15923.16 (14130.01 to 17769.23) | -0.0007 (-0.0008 to -0.0006) |
| Cyprus | 63998 (55600 to 73046) | 7805.36 (6777.77 to 8882.85) |  | 130886 (114300 to 149313) | 7808.77 (6782.82 to 8885.45) | 0.0014 (0.0011 to 0.0017) |
| Czechia | 1154882 (1013442 to 1311581) | 9663.85 (8455.47 to 10950.82) |  | 1403776 (1237991 to 1602054) | 9653.03 (8450.87 to 10936.24) | -0.0039 (-0.004 to -0.0038) |
| Côte d'Ivoire | 835046 (708045 to 957935) | 10999.54 (9592.16 to 12420.09) |  | 2071077 (1764926 to 2371587) | 11004.54 (9604.64 to 12423.99) | 0.0015 (0.0013 to 0.0017) |
| Democratic People's Republic of Korea | 917220 (787534 to 1048106) | 4698.82 (4092.61 to 5344.99) |  | 1439218 (1250478 to 1642833) | 4672.09 (4069.89 to 5322.27) | -0.0201 (-0.0208 to -0.0193) |
| Democratic Republic of the Congo | 2690875 (2297730 to 3068401) | 11039.22 (9602.89 to 12456.72) |  | 6543528 (5573266 to 7464052) | 11031.83 (9620.71 to 12448.72) | -0.0024 (-0.0027 to -0.0022) |
| Denmark | 613268 (538463 to 691738) | 9681.37 (8426.27 to 10927.77) |  | 729084 (643928 to 823442) | 9641.73 (8436.6 to 10918.63) | -0.0139 (-0.0193 to -0.0086) |
| Djibouti | 32833 (27862 to 37819) | 10996.4 (9589.24 to 12419.69) |  | 110842 (94539 to 126831) | 10999.04 (9591.77 to 12416.35) | 0.0011 (0.0002 to 0.002) |
| Dominica | 10427 (9281 to 11590) | 15931.71 (14133.4 to 17773.42) |  | 12419 (11028 to 13818) | 15915.79 (14124.57 to 17761.57) | -0.0034 (-0.0035 to -0.0033) |
| Dominican Republic | 881209 (768212 to 993643) | 15939.99 (14140.74 to 17785.38) |  | 1701171 (1498529 to 1901584) | 15923.82 (14129.58 to 17772.58) | -0.0037 (-0.0041 to -0.0034) |
| Ecuador | 1223469 (1067881 to 1378203) | 15932.17 (14133.39 to 17779.76) |  | 2710469 (2394241 to 3027719) | 15933.12 (14136.28 to 17776.29) | 0.0002 (0.0001 to 0.0003) |
| Egypt | 5172188 (4438922 to 5885302) | 12124.79 (10544.95 to 13688.56) |  | 10658406 (9190474 to 12109702) | 12081.08 (10503.77 to 13639.71) | -0.0127 (-0.0133 to -0.0121) |
| El Salvador | 625360 (548868 to 702660) | 15952.31 (14148.57 to 17789.31) |  | 984765 (870430 to 1096207) | 15976.02 (14173 to 17791.38) | 0.0053 (0.0051 to 0.0055) |
| Equatorial Guinea | 30450 (26049 to 34691) | 11068.09 (9633.56 to 12500.59) |  | 105541 (89647 to 121349) | 11034.8 (9604.67 to 12454.38) | -0.0106 (-0.0112 to -0.01) |
| Eritrea | 202930 (172607 to 232382) | 11052.71 (9635.36 to 12468.57) |  | 531009 (451726 to 608635) | 11038.97 (9630.55 to 12456.54) | -0.0043 (-0.0044 to -0.0042) |
| Estonia | 204649 (180623 to 229992) | 11323.02 (9935.16 to 12749.19) |  | 198607 (175729 to 222860) | 11276.06 (9904.73 to 12699.6) | -0.0139 (-0.0149 to -0.0129) |
| Eswatini | 53695 (45783 to 61173) | 11063.77 (9644.37 to 12485.42) |  | 99246 (84645 to 113489) | 11059.06 (9648.11 to 12482.51) | -0.0014 (-0.0016 to -0.0012) |
| Ethiopia | 3588898 (3061322 to 4092094) | 11450.19 (9970.42 to 12968.41) |  | 8088339 (6885891 to 9231971) | 11445.27 (9973.45 to 12962.82) | -0.0014 (-0.0016 to -0.0012) |
| Fiji | 31562 (26731 to 36631) | 5292.12 (4569.11 to 6054.07) |  | 46754 (39875 to 53886) | 5295.76 (4574.59 to 6058.34) | 0.0023 (0.002 to 0.0026) |
| Finland | 644833 (568603 to 721895) | 10570.51 (9281.87 to 11839.26) |  | 772284 (683484 to 872232) | 10401.52 (9107.46 to 11688.82) | -0.0863 (-0.1 to -0.0726) |
| France | 4645426 (4091688 to 5259571) | 6879.69 (6007.17 to 7816.23) |  | 5880314 (5191858 to 6660499) | 6879.84 (6004.83 to 7814.37) | -0.1097 (-0.3704 to 0.1516) |
| Gabon | 78384 (67549 to 88960) | 11029.9 (9617.82 to 12449.73) |  | 163772 (140668 to 185981) | 11039.8 (9619.37 to 12451.6) | 0.0032 (0.003 to 0.0034) |
| Gambia | 67066 (57180 to 76765) | 11010.77 (9613.3 to 12425.69) |  | 171477 (146226 to 196138) | 11030.89 (9613 to 12443.56) | 0.0065 (0.0061 to 0.0068) |
| Georgia | 622694 (541948 to 704087) | 10490.19 (9140.46 to 11809.04) |  | 480727 (424857 to 539930) | 10474.87 (9129.37 to 11789.74) | -0.005 (-0.0052 to -0.0047) |
| Germany | 7644790 (6716868 to 8590353) | 7528.11 (6579.44 to 8487.26) |  | 9040426 (7969692 to 10277731) | 7658.63 (6681.36 to 8666.84) | 0.0467 (-0.0524 to 0.1458) |
| Ghana | 1094077 (932309 to 1248141) | 11034.13 (9617.42 to 12448.33) |  | 2762878 (2361147 to 3151238) | 11051.34 (9626.52 to 12472.09) | 0.0056 (0.0053 to 0.0059) |
| Greece | 1269759 (1112816 to 1444509) | 10207.87 (8841.43 to 11640.07) |  | 1459951 (1293135 to 1662356) | 10205.6 (8839.14 to 11639.59) | 0.0057 (-0.0106 to 0.022) |
| Greenland | 4463 (3801 to 5155) | 8124.04 (7100.21 to 9248.7) |  | 5375 (4660 to 6161) | 8121.54 (7091.66 to 9250.19) | -0.0017 (-0.0032 to -0.0003) |
| Grenada | 11187 (9934 to 12455) | 15939.68 (14140.83 to 17776.22) |  | 18137 (16052 to 20243) | 15916.93 (14124.84 to 17757.75) | -0.0049 (-0.005 to -0.0048) |
| Guam | 6409 (5423 to 7436) | 5277 (4556.53 to 6042.48) |  | 9441 (8171 to 10810) | 5286.68 (4567.31 to 6050.11) | 0.0074 (0.0058 to 0.009) |
| Guatemala | 860656 (750230 to 969572) | 15936.8 (14136.98 to 17782.59) |  | 2435067 (2130647 to 2737075) | 15959.2 (14157.98 to 17783.48) | 0.005 (0.0048 to 0.0052) |
| Guinea | 466298 (402233 to 528299) | 11040.6 (9613.14 to 12451.07) |  | 917183 (782339 to 1045744) | 11041.64 (9608.98 to 12451.61) | 0.0003 (-0.0001 to 0.0007) |
| Guinea-Bissau | 69336 (59129 to 79094) | 11048.65 (9613.49 to 12467.02) |  | 143770 (122029 to 164966) | 11049.18 (9623.57 to 12464.54) | 0.0002 (0.0001 to 0.0003) |
| Guyana | 95683 (83188 to 107917) | 15933.9 (14136.07 to 17780.14) |  | 120250 (106155 to 134597) | 15937.33 (14137.13 to 17778.31) | 0.0004 (-0.0001 to 0.001) |
| Haiti | 743087 (649415 to 837534) | 15946.33 (14142.88 to 17788.35) |  | 1684267 (1466907 to 1900889) | 15952.27 (14148.26 to 17787.19) | 0.0013 (0.0009 to 0.0018) |
| Honduras | 490162 (428110 to 552036) | 15938.44 (14138.59 to 17784.58) |  | 1329334 (1161770 to 1495399) | 15953.31 (14148.49 to 17787.84) | 0.0033 (0.0031 to 0.0035) |
| Hungary | 1258732 (1090894 to 1439581) | 10131.43 (8666.13 to 11585.9) |  | 1353876 (1178155 to 1542521) | 10121.85 (8656.66 to 11575.39) | -0.0029 (-0.0049 to -0.0009) |
| Iceland | 16437 (14335 to 18643) | 6169.85 (5393.98 to 7037.17) |  | 26344 (23091 to 29796) | 6251.15 (5423.64 to 7096.89) | 0.0485 (0.0412 to 0.0558) |
| India | 88274714 (76551427 to 99781642) | 13433.44 (11844.09 to 15077.69) |  | 181552690 (158485093 to 204380384) | 13423.86 (11828.07 to 15065.83) | -0.0037 (-0.0209 to 0.0135) |
| Indonesia | 8099123 (6914979 to 9336116) | 5551.77 (4827.87 to 6310.81) |  | 14734723 (12647009 to 16908372) | 5548.24 (4827.47 to 6305.82) | -0.0022 (-0.0024 to -0.0021) |
| Iran (Islamic Republic of) | 4549645 (3892677 to 5195403) | 11409.38 (9947.09 to 12910.68) |  | 10406617 (8985571 to 11872520) | 11433.69 (9963.6 to 12938.89) | 0.0351 (-0.048 to 0.1183) |
| Iraq | 1420264 (1224653 to 1620005) | 12127.16 (10550.11 to 13691.22) |  | 4397322 (3770700 to 5021402) | 12124.16 (10547.23 to 13687.46) | -0.0001 (-0.0028 to 0.0027) |
| Ireland | 286310 (250034 to 326138) | 7804.17 (6782.38 to 8884.43) |  | 463291 (405400 to 531143) | 7805.28 (6782.21 to 8882.7) | 0.001 (-0.0003 to 0.0022) |
| Israel | 393220 (344056 to 445484) | 8372.6 (7314.88 to 9477.53) |  | 812620 (714264 to 919013) | 8364.97 (7307.88 to 9465.69) | 0.0185 (-0.0276 to 0.0647) |
| Italy | 6870343 (6067825 to 7756648) | 9698.72 (8496.6 to 10954.12) |  | 8321445 (7377296 to 9400920) | 9697.97 (8496.99 to 10948.32) | -0.0015 (-0.0268 to 0.0239) |
| Jamaica | 312861 (276513 to 350526) | 15940.27 (14138.32 to 17783.82) |  | 484975 (429653 to 539946) | 15933.59 (14135.39 to 17776.72) | -0.0013 (-0.0015 to -0.0012) |
| Japan | 8998669 (7859770 to 10307199) | 5892.17 (5129.8 to 6733.56) |  | 11321847 (9986889 to 12925107) | 5920.25 (5159.24 to 6767.49) | -0.0034 (-0.0473 to 0.0406) |
| Jordan | 299423 (256312 to 344873) | 12087.21 (10504.98 to 13652.26) |  | 1240794 (1062180 to 1411378) | 12044.92 (10471.57 to 13608.46) | -0.011 (-0.0134 to -0.0085) |
| Kazakhstan | 1568173 (1352647 to 1773785) | 10493.68 (9148.86 to 11812.64) |  | 1996549 (1720606 to 2257841) | 10482.68 (9134.88 to 11800.97) | -0.0035 (-0.0038 to -0.0032) |
| Kenya | 1546667 (1319381 to 1765633) | 11451.5 (9978.57 to 12973) |  | 4213624 (3586499 to 4819698) | 11455.98 (9978.82 to 12971.11) | 0.0013 (0.0012 to 0.0014) |
| Kiribati | 2974 (2530 to 3445) | 5310.27 (4584.51 to 6073.18) |  | 5255 (4452 to 6084) | 5317.25 (4592.24 to 6083.38) | 0.0042 (0.0025 to 0.006) |
| Kuwait | 184328 (156261 to 213093) | 11863.18 (10321.73 to 13419.45) |  | 608886 (515389 to 700088) | 12012.63 (10436.53 to 13567.28) | 0.0432 (0.0405 to 0.0459) |
| Kyrgyzstan | 376223 (325671 to 426567) | 10484.54 (9137.5 to 11802.97) |  | 620682 (533643 to 702666) | 10472.86 (9126.37 to 11788.95) | -0.0034 (-0.0042 to -0.0026) |
| Lao People's Democratic Republic | 154296 (131570 to 178176) | 5304.42 (4575.46 to 6067.32) |  | 334637 (283650 to 387037) | 5295.61 (4571.58 to 6056.58) | -0.0057 (-0.006 to -0.0055) |
| Latvia | 352372 (311190 to 396038) | 11326.92 (9937.87 to 12753.23) |  | 295333 (261977 to 331958) | 11294.18 (9915.31 to 12718.93) | -0.0095 (-0.0102 to -0.0089) |
| Lebanon | 327011 (282025 to 370329) | 12164.43 (10568.57 to 13731.74) |  | 661104 (574679 to 749222) | 12200.07 (10596.55 to 13759.66) | 0.0093 (0.007 to 0.0117) |
| Lesotho | 141634 (121790 to 160477) | 11045.3 (9636 to 12459.73) |  | 198690 (170232 to 226408) | 11043.59 (9633.24 to 12462.21) | -0.0005 (-0.0008 to -0.0002) |
| Liberia | 148999 (128848 to 168428) | 11022.7 (9609.97 to 12436.24) |  | 389027 (332002 to 444524) | 11015.24 (9607.22 to 12431.25) | -0.0029 (-0.004 to -0.0017) |
| Libya | 346910 (299523 to 396970) | 12022.81 (10451.82 to 13587.26) |  | 873439 (748144 to 991226) | 12102.68 (10526.14 to 13666.37) | 0.023 (0.0223 to 0.0237) |
| Lithuania | 492512 (433970 to 555496) | 11932.16 (10471.05 to 13446.73) |  | 453805 (403393 to 506727) | 11934.23 (10449.2 to 13393.52) | 0.0007 (0.0002 to 0.0012) |
| Luxembourg | 36813 (32057 to 41879) | 7810.22 (6789.23 to 8892.23) |  | 62312 (54479 to 70887) | 7791.01 (6771.66 to 8864.24) | -0.0083 (-0.0089 to -0.0077) |
| Madagascar | 845024 (721388 to 963168) | 11021.73 (9594.84 to 12437.62) |  | 2074287 (1767628 to 2368197) | 11027.16 (9605.55 to 12442.31) | 0.0017 (0.0016 to 0.0018) |
| Malawi | 668510 (570192 to 762215) | 11036.03 (9614.56 to 12450.63) |  | 1345606 (1148990 to 1539508) | 11044.89 (9632.86 to 12457.36) | 0.0031 (0.0025 to 0.0037) |
| Malaysia | 740273 (628024 to 857190) | 5294.26 (4570.91 to 6057.17) |  | 1692761 (1446736 to 1950408) | 5285.28 (4564.63 to 6047.4) | -0.006 (-0.0063 to -0.0057) |
| Maldives | 7461 (6370 to 8656) | 5264.38 (4542.57 to 6030.8) |  | 27472 (23112 to 32136) | 5243.59 (4522.52 to 6010.18) | -0.0143 (-0.0161 to -0.0125) |
| Mali | 622839 (534618 to 708639) | 11037.14 (9615.07 to 12448.17) |  | 1489345 (1270421 to 1692590) | 11025.43 (9606.33 to 12438.03) | -0.0038 (-0.0042 to -0.0034) |
| Malta | 32159 (28018 to 36631) | 7815.67 (6785.94 to 8898.04) |  | 46906 (41319 to 53315) | 7792.34 (6769.2 to 8865.27) | -0.0105 (-0.0108 to -0.0103) |
| Marshall Islands | 1489 (1259 to 1724) | 5288.98 (4570.67 to 6053.66) |  | 2645 (2239 to 3067) | 5284.55 (4562.74 to 6046.58) | -0.0032 (-0.0044 to -0.002) |
| Mauritania | 152038 (130407 to 172225) | 11034.5 (9613.54 to 12448.03) |  | 323114 (276921 to 366378) | 11031.02 (9600.72 to 12443.91) | -0.0011 (-0.0013 to -0.001) |
| Mauritius | 53231 (45358 to 61436) | 5302.55 (4577.08 to 6063.16) |  | 83022 (72104 to 95468) | 5299.84 (4575.36 to 6061.76) | -0.0017 (-0.0019 to -0.0014) |
| Mexico | 10199233 (8893565 to 11465454) | 15948.74 (14119.5 to 17648.19) |  | 20500311 (18098543 to 22748369) | 15949.98 (14121.96 to 17645.72) | 0.0003 (0.0002 to 0.0004) |
| Micronesia (Federated States of) | 3701 (3142 to 4272) | 5288.73 (4568.23 to 6051.77) |  | 4853 (4131 to 5609) | 5296.29 (4575.03 to 6058.74) | 0.0048 (0.0044 to 0.0051) |
| Monaco | 3394 (2996 to 3852) | 7816.29 (6790.06 to 8898.57) |  | 4213 (3702 to 4780) | 7806.45 (6784.69 to 8884.38) | -0.0039 (-0.0048 to -0.0031) |
| Mongolia | 157055 (135297 to 179028) | 10456.43 (9105.36 to 11774.21) |  | 343108 (293194 to 391954) | 10474.43 (9125.51 to 11789.73) | 0.006 (0.0057 to 0.0063) |
| Montenegro | 62539 (54514 to 70891) | 9661.74 (8456.56 to 10943.74) |  | 75908 (66777 to 86670) | 9656.43 (8452.39 to 10940.46) | -0.0019 (-0.0021 to -0.0017) |
| Morocco | 2386495 (2052323 to 2721385) | 12155.07 (10563.81 to 13718.3) |  | 4444727 (3839151 to 5036649) | 12142.82 (10554.67 to 13706.76) | -0.0033 (-0.004 to -0.0025) |
| Mozambique | 949321 (815917 to 1081107) | 11051.43 (9623.85 to 12467.8) |  | 2027061 (1728156 to 2312895) | 11053.34 (9628.62 to 12469.66) | 0.0003 (-0.0011 to 0.0017) |
| Myanmar | 1699069 (1449333 to 1960647) | 5303.02 (4575.85 to 6064.02) |  | 2869044 (2451579 to 3302885) | 5315.08 (4588.53 to 6081.63) | 0.0079 (0.0078 to 0.0081) |
| Namibia | 107789 (92755 to 122096) | 11041.01 (9623.1 to 12454.01) |  | 218170 (187374 to 247602) | 11049.47 (9629.15 to 12465.42) | 0.0027 (0.0024 to 0.0029) |
| Nauru | 377 (317 to 438) | 5281.64 (4564.93 to 6050.66) |  | 431 (362 to 502) | 5307.11 (4584.16 to 6072.76) | 0.0163 (0.0144 to 0.0182) |
| Nepal | 1781333 (1542793 to 2014179) | 13072.38 (11492.06 to 14620.79) |  | 3575087 (3117156 to 4014574) | 13109.28 (11526.51 to 14672.08) | 0.0096 (0.0091 to 0.0101) |
| Netherlands | 992421 (868031 to 1127366) | 5616.74 (4881.79 to 6403.16) |  | 1276904 (1125006 to 1449642) | 5610.34 (4876.73 to 6393.86) | -0.005 (-0.0145 to 0.0045) |
| New Zealand | 355379 (309943 to 401001) | 9636.64 (8389.47 to 10887.46) |  | 534050 (469941 to 604760) | 9639.88 (8393.39 to 10893.21) | 0.0042 (-0.0011 to 0.0095) |
| Nicaragua | 395922 (344735 to 447504) | 15948.79 (14145.48 to 17787.91) |  | 950909 (832406 to 1068652) | 15942.51 (14143.48 to 17777.68) | -0.0013 (-0.0014 to -0.0013) |
| Niger | 517073 (441970 to 589948) | 11019.73 (9604.52 to 12434.11) |  | 1419705 (1208909 to 1616014) | 11031.56 (9597.66 to 12445.14) | 0.0038 (0.0036 to 0.0041) |
| Nigeria | 7029802 (6012250 to 7996182) | 11418.88 (9950.88 to 12941.05) |  | 16326334 (13929150 to 18621977) | 11469.85 (9987.25 to 12985.97) | 0.0157 (0.0153 to 0.0162) |
| Niue | 109 (95 to 125) | 5301.55 (4576.52 to 6062.17) |  | 98 (86 to 113) | 5297.51 (4572.98 to 6063.1) | -0.0027 (-0.0031 to -0.0023) |
| North Macedonia | 197411 (172035 to 224549) | 9654.94 (8453.68 to 10935.18) |  | 267459 (234340 to 306870) | 9649.06 (8449.16 to 10927.71) | -0.0021 (-0.0023 to -0.0019) |
| Northern Mariana Islands | 2175 (1816 to 2564) | 5251.1 (4535.5 to 6005.08) |  | 2658 (2292 to 3074) | 5281.29 (4558.55 to 6042.37) | 0.0214 (0.0162 to 0.0265) |
| Norway | 257260 (225075 to 293033) | 5014.45 (4353.65 to 5711.52) |  | 350474 (307033 to 401584) | 5082.83 (4399.26 to 5818.16) | 0.0478 (0.047 to 0.0485) |
| Oman | 168110 (143123 to 193622) | 11877.81 (10340.07 to 13428.35) |  | 564966 (475602 to 658378) | 11807.61 (10282.6 to 13351.54) | -0.0195 (-0.0564 to 0.0175) |
| Pakistan | 10072358 (8762404 to 11352165) | 13342.78 (11762.37 to 14896.93) |  | 22229700 (19220139 to 25083163) | 13359.46 (11775.69 to 14908.83) | 0.0044 (0.0043 to 0.0044) |
| Palau | 722 (617 to 835) | 5288.78 (4567.58 to 6050.55) |  | 1138 (979 to 1312) | 5270.85 (4550.23 to 6034.27) | -0.0161 (-0.0246 to -0.0075) |
| Palestine | 158339 (136330 to 181380) | 12201.24 (10598.45 to 13756.34) |  | 474374 (406618 to 543242) | 12135.31 (10553.92 to 13700.65) | -0.0184 (-0.019 to -0.0179) |
| Panama | 317071 (277243 to 356800) | 15921.01 (14127.1 to 17767.17) |  | 669729 (593093 to 746701) | 15924.4 (14130.24 to 17772.53) | 0.0008 (0.0006 to 0.0009) |
| Papua New Guinea | 156466 (132833 to 181044) | 5282.28 (4562.76 to 6046.66) |  | 411534 (348779 to 477855) | 5280.57 (4559.93 to 6044.76) | -0.0013 (-0.0016 to -0.001) |
| Paraguay | 494912 (432457 to 555515) | 16314.15 (14397.45 to 18170.37) |  | 1091009 (959495 to 1219126) | 16310.51 (14395.46 to 18167.8) | -0.0008 (-0.0008 to -0.0007) |
| Peru | 2674409 (2339777 to 3010171) | 15933.38 (14134.59 to 17779.73) |  | 5495940 (4857033 to 6139347) | 15932.34 (14135.52 to 17773.58) | -0.0004 (-0.0007 to -0.0001) |
| Philippines | 2562197 (2190022 to 2949889) | 5549.45 (4827.69 to 6308.53) |  | 5566045 (4783871 to 6392905) | 5550.86 (4830.54 to 6311.02) | 0.001 (0.0008 to 0.0013) |
| Poland | 5685187 (4980700 to 6397514) | 13682.67 (12013.64 to 15329.3) |  | 7050652 (6252689 to 7903104) | 13668.06 (11998.99 to 15316.07) | -0.0041 (-0.0092 to 0.001) |
| Portugal | 991759 (863156 to 1132812) | 8457.9 (7296.38 to 9630.73) |  | 1258885 (1105123 to 1439317) | 8452.74 (7292.76 to 9628.74) | -0.0028 (-0.0067 to 0.0012) |
| Puerto Rico | 576682 (512505 to 642830) | 15951.16 (14147.85 to 17785.38) |  | 729015 (654404 to 807228) | 15947.1 (14144.94 to 17782.26) | -0.0008 (-0.001 to -0.0007) |
| Qatar | 49673 (41421 to 58066) | 11677.95 (10183.92 to 13236.27) |  | 390279 (326703 to 456039) | 11536.01 (10067.61 to 13070.27) | -0.0436 (-0.0466 to -0.0406) |
| Republic of Korea | 2863967 (2457966 to 3276780) | 6802.59 (5958.48 to 7784.93) |  | 4982276 (4378194 to 5742303) | 6784.53 (5942.11 to 7757.37) | 0.1329 (-0.175 to 0.4417) |
| Republic of Moldova | 514820 (450496 to 580760) | 11311.88 (9926.08 to 12737.36) |  | 542853 (478314 to 611340) | 11288.07 (9910.59 to 12710.21) | -0.007 (-0.0074 to -0.0065) |
| Romania | 2489516 (2179576 to 2842911) | 9659.83 (8455.45 to 10946.92) |  | 2517417 (2224020 to 2870353) | 9657.15 (8453.25 to 10940.9) | -0.0008 (-0.001 to -0.0006) |
| Russian Federation | 19155806 (16836407 to 21559377) | 11232.73 (9858.86 to 12650.75) |  | 21217288 (18767607 to 23892031) | 11220.55 (9860.61 to 12639.47) | 0.0518 (-0.1155 to 0.2193) |
| Rwanda | 495256 (422675 to 566726) | 11046.39 (9612.52 to 12465.47) |  | 1051623 (897382 to 1202178) | 11051.27 (9623.44 to 12471.2) | 0.0031 (0.0011 to 0.005) |
| Saint Kitts and Nevis | 5728 (5074 to 6418) | 15939.77 (14137.51 to 17781.89) |  | 11013 (9730 to 12333) | 15925.94 (14129.4 to 17770.38) | -0.003 (-0.0031 to -0.0028) |
| Saint Lucia | 17074 (15043 to 19208) | 15947.95 (14143.96 to 17785.63) |  | 32900 (29251 to 36665) | 15929.51 (14133.54 to 17774.42) | -0.004 (-0.0041 to -0.004) |
| Saint Vincent and the Grenadines | 13665 (12031 to 15387) | 15932.32 (14136.03 to 17774.94) |  | 20145 (17929 to 22444) | 15917.08 (14124.54 to 17764.46) | -0.0033 (-0.0035 to -0.0031) |
| Samoa | 6291 (5392 to 7234) | 5288.49 (4567.08 to 6049.4) |  | 9406 (8037 to 10816) | 5288.13 (4569.3 to 6053.41) | -0.0003 (-0.0006 to 0.0001) |
| San Marino | 2156 (1885 to 2447) | 7799.23 (6776.85 to 8876.11) |  | 3379 (2967 to 3840) | 7818.94 (6791.87 to 8900.61) | 0.0087 (0.0085 to 0.0088) |
| Sao Tome and Principe | 8651 (7476 to 9784) | 11038.27 (9611.55 to 12449.83) |  | 17821 (15248 to 20288) | 11021.38 (9607.17 to 12439.01) | -0.0055 (-0.0059 to -0.0051) |
| Saudi Arabia | 1381200 (1180139 to 1591396) | 11907.64 (10361.33 to 13460.43) |  | 4648684 (3936029 to 5336535) | 11914.66 (10361.7 to 13458.51) | 0.0016 (-0.0001 to 0.0032) |
| Senegal | 522698 (446635 to 593964) | 11031.81 (9604.14 to 12443.67) |  | 1208073 (1034100 to 1370437) | 11032.96 (9606.29 to 12446.74) | 0.0004 (0.0002 to 0.0007) |
| Serbia | 1033791 (904330 to 1182398) | 9657.33 (8453.67 to 10940.9) |  | 1108016 (974707 to 1261503) | 9654.71 (8452.82 to 10938.26) | -0.001 (-0.0013 to -0.0007) |
| Seychelles | 3401 (2931 to 3903) | 5300.81 (4580.51 to 6061.84) |  | 6109 (5245 to 7011) | 5281.37 (4560.19 to 6043.54) | -0.0125 (-0.0134 to -0.0116) |
| Sierra Leone | 282723 (242856 to 320504) | 11028.44 (9606.41 to 12440.93) |  | 646616 (550775 to 739573) | 11022.15 (9607.52 to 12436.7) | -0.0025 (-0.0031 to -0.002) |
| Singapore | 228633 (196056 to 261105) | 7286.65 (6355.91 to 8224.79) |  | 551015 (478040 to 628107) | 7335.27 (6373.68 to 8317.94) | 0.0215 (0.0058 to 0.0372) |
| Slovakia | 547964 (480228 to 621885) | 9664.68 (8456.97 to 10950.28) |  | 699458 (615216 to 798470) | 9657.7 (8452.82 to 10942.84) | -0.0025 (-0.0027 to -0.0024) |
| Slovenia | 219940 (192675 to 249494) | 9667.09 (8459.71 to 10954.52) |  | 277823 (244981 to 316625) | 9649.88 (8447.72 to 10929.97) | -0.0062 (-0.0063 to -0.0061) |
| Solomon Islands | 11534 (9826 to 13329) | 5270.97 (4548.37 to 6034.73) |  | 26269 (22210 to 30487) | 5288.83 (4564.61 to 6052.71) | 0.0117 (0.0115 to 0.0119) |
| Somalia | 484208 (413428 to 555092) | 11031.91 (9614.26 to 12447.77) |  | 1367849 (1167496 to 1567540) | 11037.38 (9626.07 to 12456.86) | 0.0015 (0.0009 to 0.002) |
| South Africa | 3310762 (2833526 to 3768860) | 11472.91 (9991.45 to 12986.59) |  | 6308655 (5417195 to 7175995) | 11467.17 (9985.68 to 12982.6) | -0.0017 (-0.0022 to -0.0013) |
| South Sudan | 414230 (354262 to 473046) | 10984.47 (9578.79 to 12405.46) |  | 667916 (572209 to 757368) | 11029.44 (9603.12 to 12438.37) | 0.0151 (0.0138 to 0.0163) |
| Spain | 3157132 (2766564 to 3594863) | 7011.94 (6095.1 to 7963.25) |  | 4413017 (3862538 to 4986140) | 7000.31 (6082.16 to 7946.51) | 0.0901 (-0.1109 to 0.2915) |
| Sri Lanka | 794530 (675493 to 918102) | 5290.9 (4567.03 to 6052.99) |  | 1284276 (1110152 to 1474311) | 5309.03 (4582.23 to 6072.13) | 0.0118 (0.0115 to 0.0121) |
| Sudan | 1677724 (1442959 to 1914803) | 12133.81 (10548.65 to 13701.29) |  | 3794497 (3251923 to 4349137) | 12118.61 (10535.58 to 13691.27) | -0.0044 (-0.0048 to -0.004) |
| Suriname | 52570 (46123 to 59023) | 15927.64 (14135.6 to 17767.14) |  | 97038 (85929 to 108270) | 15937.19 (14137.12 to 17777.55) | 0.002 (0.0017 to 0.0024) |
| Sweden | 717117 (629712 to 808841) | 6688.55 (5848.49 to 7562.39) |  | 951288 (834579 to 1085119) | 7124.05 (6180.66 to 8116.26) | 0.2159 (0.1829 to 0.249) |
| Switzerland | 396760 (346806 to 448095) | 4660.12 (4046.51 to 5304.87) |  | 550188 (480244 to 626731) | 4649.86 (4038.28 to 5292.21) | 0.0491 (-0.0456 to 0.1439) |
| Syrian Arab Republic | 999763 (859777 to 1146059) | 12107.84 (10527.73 to 13672.08) |  | 1686365 (1469093 to 1910510) | 12189.29 (10587.24 to 13764.69) | 0.0233 (0.0211 to 0.0255) |
| Taiwan (Province of China) | 998200 (859896 to 1142721) | 4970.5 (4322.53 to 5635.88) |  | 1617133 (1414226 to 1837863) | 5150.38 (4464.23 to 5858.15) | 0.0827 (-0.0171 to 0.1825) |
| Tajikistan | 389180 (336209 to 442285) | 10463.64 (9111.76 to 11781.85) |  | 830225 (708336 to 949221) | 10451.87 (9098.01 to 11764.79) | -0.0038 (-0.0043 to -0.0033) |
| Thailand | 2703826 (2298730 to 3130808) | 5302.96 (4575.73 to 6064.06) |  | 4749586 (4130654 to 5458807) | 5303.14 (4575.94 to 6063.49) | 0 (-0.0006 to 0.0007) |
| Timor-Leste | 28905 (24403 to 33498) | 5285.47 (4565.01 to 6049.13) |  | 54793 (46878 to 63063) | 5291.01 (4566.15 to 6056.99) | 0.0032 (0.0019 to 0.0045) |
| Togo | 240454 (204936 to 275226) | 11048.32 (9613.18 to 12466.89) |  | 650808 (555763 to 740884) | 11055.75 (9638.41 to 12476.86) | 0.0025 (0.0022 to 0.0028) |
| Tokelau | 70 (61 to 81) | 5311.57 (4584.05 to 6078.16) |  | 70 (60 to 80) | 5286.48 (4563.41 to 6049.76) | -0.0151 (-0.0265 to -0.0037) |
| Tonga | 3753 (3210 to 4308) | 5300.55 (4572.53 to 6065.46) |  | 4665 (4019 to 5343) | 5303.22 (4576.36 to 6066.05) | 0.0016 (0.0014 to 0.0018) |
| Trinidad and Tobago | 168436 (147307 to 189407) | 15929 (14132.51 to 17775.9) |  | 263953 (234481 to 294273) | 15923.1 (14128.04 to 17770.08) | -0.0013 (-0.0014 to -0.0012) |
| Tunisia | 815443 (702535 to 928821) | 12130.96 (10547.14 to 13695.32) |  | 1565491 (1358400 to 1765225) | 12159.64 (10565.7 to 13724.44) | 0.0082 (0.008 to 0.0084) |
| Turkey | 6546387 (5651344 to 7426743) | 13346.28 (11668.33 to 15000.21) |  | 13227257 (12207283 to 14247243) | 14226.8 (13132.37 to 15338.41) | 0.2016 (0.1621 to 0.2412) |
| Turkmenistan | 281053 (242078 to 320467) | 10478.21 (9129.18 to 11796.73) |  | 507939 (436190 to 573904) | 10459.35 (9106.55 to 11773.59) | -0.0061 (-0.0064 to -0.0057) |
| Tuvalu | 431 (368 to 496) | 5323.99 (4597.42 to 6099.6) |  | 597 (513 to 687) | 5291.57 (4572.96 to 6054.31) | -0.0212 (-0.0214 to -0.021) |
| Uganda | 1129337 (965238 to 1286843) | 11030.88 (9609.69 to 12445.79) |  | 2764055 (2353038 to 3164567) | 11050.46 (9627.9 to 12467.45) | 0.0062 (0.0059 to 0.0064) |
| Ukraine | 7227630 (6342238 to 8128793) | 11678.51 (10198.96 to 13060.93) |  | 6897374 (6069560 to 7755200) | 11664.72 (10187.24 to 13045.1) | -0.0041 (-0.0048 to -0.0034) |
| United Arab Emirates | 197045 (164645 to 230135) | 11701.97 (10190.67 to 13234) |  | 1369742 (1125122 to 1610981) | 11624.76 (10132.8 to 13163.22) | -0.0204 (-0.0302 to -0.0106) |
| United Kingdom | 7236932 (6373584 to 8141689) | 10390.96 (9079.17 to 11766.56) |  | 8936644 (7907755 to 10096884) | 10335 (9002.04 to 11713.93) | 0.0634 (-0.1526 to 0.2799) |
| United Republic of Tanzania | 1789203 (1527918 to 2034906) | 11039.52 (9613.17 to 12451.85) |  | 4259690 (3643902 to 4846923) | 11039.84 (9614.6 to 12452) | -0.0001 (-0.0003 to 0.0002) |
| United States of America | 31495485 (27470837 to 35624833) | 10993.58 (9577.18 to 12428.79) |  | 39713666 (34961099 to 45039243) | 9659.8 (8405.19 to 10935.68) | -0.4966 (-0.6 to -0.3931) |
| United States Virgin Islands | 16319 (14388 to 18270) | 15953.83 (14148.61 to 17787.89) |  | 20589 (18379 to 22877) | 15955.27 (14149.14 to 17786.14) | 0.0004 (0.0001 to 0.0007) |
| Uruguay | 455008 (399487 to 510776) | 13628.57 (11864.93 to 15357.56) |  | 552881 (487816 to 619860) | 13631.52 (11868.97 to 15361.11) | 0.0007 (0.0007 to 0.0008) |
| Uzbekistan | 1592696 (1373058 to 1814002) | 10474.77 (9126.54 to 11794.6) |  | 3225525 (2758812 to 3666699) | 10467.93 (9122.81 to 11784.4) | -0.0021 (-0.0025 to -0.0017) |
| Vanuatu | 5489 (4655 to 6351) | 5274.43 (4554.36 to 6038.77) |  | 12471 (10631 to 14405) | 5285.43 (4561.7 to 6049.72) | 0.0069 (0.0066 to 0.0072) |
| Venezuela (Bolivarian Republic of) | 2356908 (2052793 to 2657408) | 15934.83 (14136.05 to 17778.18) |  | 4771478 (4227119 to 5326240) | 15941.49 (14138.75 to 17783.93) | 0.0017 (0.0012 to 0.0022) |
| Viet Nam | 2792372 (2394737 to 3213117) | 5319.57 (4593.02 to 6089.07) |  | 5617619 (4803811 to 6452749) | 5307.52 (4581.83 to 6068.58) | -0.0079 (-0.0081 to -0.0077) |
| Yemen | 982139 (844897 to 1123303) | 12150.94 (10566.77 to 13718.77) |  | 2821544 (2420964 to 3224125) | 12144.72 (10557.29 to 13707.9) | -0.0019 (-0.0024 to -0.0014) |
| Zambia | 522241 (444843 to 595374) | 11022.69 (9593.19 to 12437.08) |  | 1349787 (1145999 to 1548573) | 11027.46 (9619.57 to 12443.53) | 0.0016 (0.0014 to 0.0017) |
| Zimbabwe | 703520 (600058 to 800725) | 11036.32 (9616.78 to 12447.1) |  | 1210284 (1033952 to 1381878) | 11059.06 (9641.1 to 12485.05) | 0.0072 (0.0069 to 0.0075) |

UI: uncertainty interval, CI: confidence interval, AAPC, average annual percent change.
