# Supplementary material for: Burden of gastroesophageal reflux disease in 204 countries and territories, 1990–2019: a systematic analysis for the Global Burden of disease study 2019
Source: BMC Public Health. 2023 Mar 29;23:582. doi: 10.1186/s12889-023-15272-z (PMC10053627; doi:10.1186/s12889-023-15272-z)
Supplement: Supplementary file 5 — Table S4. YLDs of gastro-oesophageal reflux disease in 1990 and 2019 with AAPC from 1990 and 2019 at national level, both sexs. [file 12889_2023_15272_MOESM5_ESM.docx]

Table S4. YLDs of gastro-oesophageal reflux disease in 1990 and 2019 with AAPC from 1990 and 2019 at national level, both sexs.

| location | 1990 | |  | 2019 | | AAPC % (95% CI)  1990-2019 |
| --- | --- | --- | --- | --- | --- | --- |
|  | Cases (95% UI) | Age-standardised YLDs per  100 000 population (95% UI) |  | Cases (95% UI) | Age-standardised YLDs per  100 000 population (95% UI) |  |
| Afghanistan | 7460 (3812 to 13462) | 92.58 (47.62 to 167.21) |  | 23476 (12178 to 42528) | 92.21 (47.65 to 167.08) | -0.012 (-0.0154 to -0.0087) |
| Albania | 1412 (720 to 2539) | 50.58 (25.74 to 91.38) |  | 1714 (875 to 3131) | 50.67 (25.97 to 92.11) | -0.0018 (-0.0335 to 0.0299) |
| Algeria | 17040 (8842 to 30946) | 93.81 (48.57 to 169.81) |  | 39494 (20374 to 71252) | 93.66 (48.63 to 169.01) | -0.0052 (-0.0064 to -0.004) |
| American Samoa | 15 (8 to 27) | 40.64 (20.77 to 74.11) |  | 21 (11 to 39) | 40.5 (20.66 to 74.2) | -0.0101 (-0.0139 to -0.0063) |
| Andorra | 38 (19 to 69) | 59.98 (30.66 to 108.61) |  | 68 (34 to 125) | 60.04 (30.75 to 109.03) | 0.002 (-0.007 to 0.0111) |
| Angola | 5641 (2897 to 9944) | 84.41 (43.7 to 150.1) |  | 16495 (8551 to 29267) | 84.83 (44.07 to 151.86) | 0.0178 (0.0153 to 0.0202) |
| Antigua and Barbuda | 69 (36 to 124) | 123.29 (64.13 to 218.12) |  | 127 (66 to 224) | 122.96 (63.84 to 217.33) | -0.0084 (-0.0101 to -0.0067) |
| Argentina | 33697 (17313 to 60602) | 105.24 (54.03 to 189.43) |  | 51839 (26830 to 92995) | 105.14 (54.26 to 187.94) | -0.0569 (-0.1546 to 0.0409) |
| Armenia | 2590 (1337 to 4762) | 80.74 (41.46 to 147.27) |  | 2945 (1496 to 5386) | 80.92 (41.33 to 147.23) | 0.0074 (0.0057 to 0.0092) |
| Australia | 12133 (6175 to 22050) | 65.32 (33.35 to 118.04) |  | 19799 (10095 to 35988) | 65.36 (33.27 to 118.9) | -0.0074 (-0.144 to 0.1295) |
| Austria | 7434 (3797 to 13345) | 78.64 (40.07 to 140.56) |  | 9379 (4722 to 16829) | 78.52 (40.18 to 140.19) | -0.0069 (-0.0105 to -0.0032) |
| Azerbaijan | 5117 (2601 to 9212) | 81.06 (41.53 to 146.43) |  | 9163 (4671 to 16796) | 80.87 (41.37 to 146.52) | -0.0082 (-0.0091 to -0.0074) |
| Bahamas | 280 (145 to 499) | 123.31 (63.79 to 219.46) |  | 520 (270 to 919) | 123.09 (64.04 to 217.48) | -0.0061 (-0.008 to -0.0042) |
| Bahrain | 417 (213 to 752) | 91.81 (47.14 to 166.35) |  | 1600 (819 to 2889) | 91.14 (46.92 to 164.49) | -0.0258 (-0.0282 to -0.0234) |
| Bangladesh | 71930 (37146 to 126778) | 99.1 (51.12 to 176.9) |  | 154463 (79488 to 276852) | 100.18 (52.01 to 179.08) | 0.0606 (-0.3323 to 0.4551) |
| Barbados | 326 (170 to 577) | 123.54 (64.11 to 219.19) |  | 459 (237 to 817) | 123.24 (64.16 to 218.35) | -0.0086 (-0.0102 to -0.007) |
| Belarus | 10270 (5226 to 18510) | 87.06 (44.24 to 155.22) |  | 10749 (5435 to 19226) | 87.13 (44.48 to 156.14) | 0.0036 (0.0022 to 0.0051) |
| Belgium | 7915 (3971 to 14295) | 64.78 (32.75 to 115.77) |  | 9707 (4937 to 17405) | 65.6 (33.14 to 117.89) | 0.0406 (0.0154 to 0.0659) |
| Belize | 159 (83 to 283) | 123.25 (64.18 to 219) |  | 463 (240 to 818) | 123 (63.83 to 218.39) | -0.0065 (-0.0082 to -0.0049) |
| Benin | 2499 (1286 to 4439) | 84.78 (43.88 to 151.48) |  | 6979 (3608 to 12326) | 85.06 (43.87 to 152.23) | 0.0113 (0.0084 to 0.0143) |
| Bermuda | 84 (43 to 150) | 123.5 (64.23 to 218.61) |  | 107 (55 to 187) | 123.42 (64.09 to 218.38) | -0.0028 (-0.0036 to -0.0019) |
| Bhutan | 418 (216 to 749) | 100.11 (51.9 to 180.94) |  | 738 (381 to 1342) | 100.27 (51.81 to 182.11) | 0.0053 (0.0035 to 0.0071) |
| Bolivia (Plurinational State of) | 5746 (2993 to 10186) | 122.64 (63.52 to 216.77) |  | 13385 (6938 to 23713) | 122.87 (63.88 to 218.07) | 0.0075 (0.0064 to 0.0085) |
| Bosnia and Herzegovina | 3515 (1785 to 6336) | 74.41 (37.8 to 133.77) |  | 3288 (1671 to 5921) | 74.24 (37.97 to 133.88) | -0.009 (-0.0152 to -0.0028) |
| Botswana | 735 (382 to 1297) | 84.75 (43.92 to 151.69) |  | 1795 (929 to 3195) | 84.1 (43.77 to 150.21) | -0.0267 (-0.0288 to -0.0247) |
| Brazil | 157188 (82468 to 280613) | 125.27 (65.32 to 221.84) |  | 301481 (155911 to 539032) | 124.74 (64.6 to 222.06) | -0.0466 (-0.1031 to 0.01) |
| Brunei Darussalam | 102 (52 to 183) | 49.69 (25.42 to 89.61) |  | 230 (117 to 416) | 49.83 (25.58 to 90.21) | 0.0111 (0.0099 to 0.0123) |
| Bulgaria | 7775 (3953 to 13928) | 74.36 (38.01 to 134.18) |  | 7194 (3674 to 13109) | 74.47 (38.12 to 134.13) | 0.0052 (0.0037 to 0.0068) |
| Burkina Faso | 5032 (2612 to 8933) | 84.68 (43.91 to 150.57) |  | 12618 (6537 to 22440) | 85.21 (44.09 to 152) | 0.0202 (0.011 to 0.0294) |
| Burundi | 3009 (1551 to 5325) | 84.65 (43.81 to 151.2) |  | 6607 (3431 to 11808) | 84.44 (43.59 to 150.92) | -0.0093 (-0.0109 to -0.0077) |
| Cabo Verde | 207 (108 to 371) | 85.65 (44.47 to 153.56) |  | 459 (236 to 815) | 85.18 (43.99 to 152.87) | -0.0191 (-0.0215 to -0.0168) |
| Cambodia | 2843 (1450 to 5126) | 40.87 (20.73 to 73.95) |  | 6311 (3209 to 11525) | 40.92 (20.81 to 74.92) | 0.0042 (0.0011 to 0.0073) |
| Cameroon | 5695 (2971 to 10009) | 84.52 (43.83 to 150.37) |  | 17368 (8899 to 30611) | 84.82 (43.83 to 151.72) | 0.0116 (0.0091 to 0.0142) |
| Canada | 17225 (8766 to 31297) | 55.39 (28.27 to 100.81) |  | 26176 (13360 to 47729) | 55.3 (28.34 to 100.28) | 0.0075 (-0.0136 to 0.0285) |
| Central African Republic | 1550 (797 to 2752) | 84.18 (43.58 to 149.34) |  | 3110 (1604 to 5479) | 84.43 (43.87 to 150.61) | 0.0139 (0.0106 to 0.0171) |
| Chad | 3217 (1658 to 5708) | 84.9 (43.97 to 152.46) |  | 7969 (4130 to 14099) | 84.71 (43.81 to 151.35) | -0.0081 (-0.0095 to -0.0067) |
| Chile | 12930 (6642 to 23155) | 105.07 (54.36 to 188.71) |  | 22469 (11610 to 40351) | 105.03 (53.95 to 187.75) | -0.0012 (-0.0035 to 0.0011) |
| China | 391785 (200086 to 704710) | 35.04 (17.8 to 63.08) |  | 631038 (317697 to 1149229) | 34.94 (17.73 to 63.02) | 0.1235 (-0.0764 to 0.3238) |
| Colombia | 32725 (16996 to 58050) | 122.89 (63.86 to 217.98) |  | 63768 (33177 to 112914) | 123.23 (63.88 to 218.22) | 0.0106 (0.0086 to 0.0127) |
| Comoros | 258 (134 to 455) | 84.83 (43.91 to 151.42) |  | 532 (275 to 943) | 85.1 (44.13 to 152.4) | 0.0091 (0.0068 to 0.0115) |
| Congo | 1355 (703 to 2400) | 84.54 (43.8 to 150.63) |  | 3540 (1832 to 6291) | 84.62 (43.94 to 150.79) | 0.0045 (0.0008 to 0.0083) |
| Cook Islands | 6 (3 to 12) | 40.69 (20.72 to 74.41) |  | 8 (4 to 15) | 40.72 (20.95 to 74.13) | 0.0006 (-0.004 to 0.0052) |
| Costa Rica | 3050 (1593 to 5400) | 123.06 (64.05 to 217.74) |  | 6403 (3327 to 11406) | 123.1 (64.09 to 218.52) | 0.0017 (-0.0016 to 0.0049) |
| Croatia | 4310 (2189 to 7786) | 74.24 (38.01 to 133.45) |  | 4311 (2176 to 7698) | 74.25 (37.85 to 133.59) | 0.001 (-0.0019 to 0.004) |
| Cuba | 13710 (7151 to 24193) | 122.98 (64 to 216.73) |  | 17766 (9139 to 31501) | 122.85 (64.23 to 217.61) | -0.0028 (-0.004 to -0.0016) |
| Cyprus | 494 (252 to 895) | 60.23 (30.68 to 109.4) |  | 1006 (511 to 1824) | 60.23 (30.83 to 108.84) | 0.0012 (-0.0009 to 0.0032) |
| Czechia | 8835 (4496 to 15871) | 74.21 (38.12 to 133.66) |  | 10651 (5454 to 19114) | 74.09 (38.08 to 133.37) | -0.0054 (-0.0106 to -0.0001) |
| Côte d'Ivoire | 6451 (3315 to 11450) | 83.96 (43.49 to 148.69) |  | 16076 (8363 to 28495) | 84.61 (44.1 to 151.08) | 0.0266 (0.0235 to 0.0296) |
| Democratic People's Republic of Korea | 7149 (3649 to 12851) | 36.44 (18.72 to 66.31) |  | 11157 (5671 to 20118) | 36.24 (18.5 to 65.15) | -0.018 (-0.0199 to -0.0162) |
| Democratic Republic of the Congo | 20657 (10735 to 36656) | 84.01 (43.67 to 148.73) |  | 50587 (26381 to 88887) | 84.51 (43.89 to 150.49) | 0.019 (0.014 to 0.024) |
| Denmark | 4697 (2442 to 8390) | 74.52 (38.68 to 132.31) |  | 5569 (2866 to 9860) | 74.28 (38.37 to 132.3) | -0.0106 (-0.0175 to -0.0036) |
| Djibouti | 256 (132 to 456) | 84.76 (43.83 to 151.35) |  | 862 (447 to 1528) | 84.86 (43.81 to 150.7) | 0.0043 (0.0029 to 0.0057) |
| Dominica | 81 (42 to 142) | 123.14 (64.1 to 217.91) |  | 95 (50 to 170) | 122.64 (63.77 to 217.36) | -0.0139 (-0.0157 to -0.0122) |
| Dominican Republic | 6860 (3533 to 12226) | 123.35 (64.22 to 219.08) |  | 13175 (6841 to 23384) | 123.03 (63.96 to 217.58) | -0.0092 (-0.0114 to -0.007) |
| Ecuador | 9513 (4955 to 16854) | 123.12 (63.7 to 217.22) |  | 20990 (10919 to 37301) | 123.09 (63.94 to 217.87) | -0.0016 (-0.0022 to -0.0011) |
| Egypt | 40190 (20917 to 72354) | 93.6 (48.26 to 169.07) |  | 82884 (42971 to 149220) | 93.37 (48.28 to 168.4) | -0.0073 (-0.0105 to -0.0041) |
| El Salvador | 4837 (2516 to 8573) | 122.76 (64.14 to 217.09) |  | 7588 (3955 to 13347) | 122.96 (63.99 to 217.11) | 0.0061 (0.0047 to 0.0074) |
| Equatorial Guinea | 233 (121 to 412) | 84.15 (43.74 to 149.97) |  | 819 (416 to 1462) | 84.6 (43.77 to 151.22) | 0.0191 (0.016 to 0.0222) |
| Eritrea | 1565 (814 to 2757) | 84.4 (43.93 to 151.17) |  | 4114 (2127 to 7276) | 84.62 (43.68 to 151.77) | 0.0085 (0.0045 to 0.0125) |
| Estonia | 1567 (802 to 2796) | 87 (44.44 to 155.06) |  | 1517 (768 to 2705) | 87 (44.46 to 155.83) | -0.0011 (-0.0046 to 0.0024) |
| Eswatini | 417 (215 to 738) | 84.98 (44.3 to 152.29) |  | 761 (391 to 1351) | 84.01 (43.55 to 149.39) | -0.0396 (-0.0416 to -0.0375) |
| Ethiopia | 27721 (14351 to 49035) | 87.6 (45.17 to 156.48) |  | 62805 (32744 to 110773) | 88.06 (45.23 to 158.29) | 0.0199 (0.0175 to 0.0223) |
| Fiji | 245 (125 to 438) | 40.64 (20.68 to 73.77) |  | 360 (183 to 661) | 40.52 (20.47 to 74.46) | -0.0096 (-0.0178 to -0.0014) |
| Finland | 4938 (2533 to 8807) | 81.25 (42.02 to 144.48) |  | 5877 (3008 to 10475) | 80.06 (41.03 to 143.9) | -0.0574 (-0.1136 to -0.0011) |
| France | 35713 (18022 to 64339) | 53.09 (26.72 to 95.82) |  | 45017 (22699 to 81472) | 53.16 (26.92 to 95.68) | 0.098 (-0.2721 to 0.4694) |
| Gabon | 604 (312 to 1075) | 84.55 (43.71 to 150.7) |  | 1264 (657 to 2228) | 84.65 (43.69 to 151.12) | 0.0033 (0.0011 to 0.0054) |
| Gambia | 521 (266 to 921) | 84.72 (43.88 to 151.19) |  | 1330 (689 to 2360) | 84.84 (43.81 to 151.64) | 0.0045 (0.0012 to 0.0077) |
| Georgia | 4812 (2450 to 8837) | 81.09 (41.42 to 146.78) |  | 3678 (1875 to 6664) | 80.73 (41.35 to 146.78) | -0.0154 (-0.018 to -0.0128) |
| Germany | 58616 (29805 to 107482) | 57.99 (29.57 to 105.69) |  | 68850 (34832 to 123722) | 58.97 (29.97 to 105.58) | 0.0456 (-0.0515 to 0.1428) |
| Ghana | 8475 (4371 to 14966) | 84.8 (44.12 to 150.79) |  | 21452 (11103 to 38085) | 85.17 (44.29 to 152.31) | 0.0136 (0.0118 to 0.0154) |
| Greece | 9770 (4980 to 17753) | 78.85 (40.39 to 143.81) |  | 11151 (5649 to 20105) | 78.79 (40.5 to 144.24) | 0.0046 (-0.0099 to 0.019) |
| Greenland | 34 (18 to 62) | 62.1 (32.03 to 112.24) |  | 41 (21 to 74) | 62.32 (32.08 to 112.36) | 0.011 (0.0078 to 0.0141) |
| Grenada | 86 (45 to 153) | 123.09 (64.1 to 217.69) |  | 140 (72 to 249) | 122.67 (63.77 to 217.15) | -0.012 (-0.0134 to -0.0105) |
| Guam | 50 (25 to 90) | 40.8 (20.79 to 74.48) |  | 73 (37 to 134) | 40.79 (20.73 to 74.4) | -0.0016 (-0.0039 to 0.0008) |
| Guatemala | 6655 (3470 to 11704) | 122.33 (63.44 to 216.12) |  | 18842 (9828 to 33547) | 122.65 (63.88 to 216.19) | 0.0094 (0.0082 to 0.0106) |
| Guinea | 3600 (1865 to 6362) | 84.83 (44.02 to 150.77) |  | 7115 (3687 to 12557) | 85.02 (44.2 to 152.12) | 0.0059 (0.0043 to 0.0075) |
| Guinea-Bissau | 537 (277 to 951) | 84.79 (43.97 to 151.51) |  | 1116 (574 to 1980) | 84.94 (44.25 to 151.8) | 0.0046 (-0.001 to 0.0101) |
| Guyana | 739 (383 to 1308) | 122.07 (63.58 to 215.75) |  | 925 (481 to 1632) | 122.06 (63.61 to 216.09) | -0.0003 (-0.0024 to 0.0019) |
| Haiti | 5741 (2976 to 10123) | 122.43 (63.68 to 217.06) |  | 13009 (6738 to 23185) | 122.34 (63.92 to 216.22) | -0.0037 (-0.0098 to 0.0024) |
| Honduras | 3806 (1963 to 6764) | 122.86 (63.6 to 217.03) |  | 10305 (5326 to 18341) | 122.89 (63.78 to 217.2) | -0.0004 (-0.0027 to 0.0019) |
| Hungary | 9619 (4919 to 17485) | 77.78 (40 to 139.64) |  | 10316 (5271 to 18560) | 77.94 (39.82 to 139.24) | 0.0088 (0.0066 to 0.0111) |
| Iceland | 127 (65 to 230) | 47.63 (24.55 to 85.92) |  | 202 (103 to 369) | 48.26 (24.27 to 87.58) | 0.0508 (0.0411 to 0.0605) |
| India | 676639 (352162 to 1202333) | 101.88 (52.89 to 183.89) |  | 1390218 (721788 to 2487108) | 102.26 (53.1 to 184.31) | 0.013 (-0.0041 to 0.0301) |
| Indonesia | 62769 (32034 to 111618) | 42.58 (21.63 to 76.22) |  | 114033 (57835 to 203758) | 42.69 (21.62 to 76.51) | 0.0088 (0.0069 to 0.0107) |
| Iran (Islamic Republic of) | 35296 (18023 to 63621) | 87.7 (44.48 to 157.73) |  | 80432 (40714 to 144806) | 88.01 (44.53 to 158.39) | 0.0409 (-0.0446 to 0.1266) |
| Iraq | 10971 (5751 to 19735) | 92.84 (47.95 to 168.08) |  | 34057 (17665 to 61372) | 93.1 (48.02 to 168.51) | 0.0096 (0.0067 to 0.0126) |
| Ireland | 2205 (1119 to 4016) | 60.21 (30.59 to 109.16) |  | 3556 (1796 to 6480) | 60.19 (30.52 to 109.23) | -0.0015 (-0.0102 to 0.0072) |
| Israel | 3039 (1544 to 5547) | 64.72 (32.72 to 117.74) |  | 6260 (3174 to 11351) | 64.63 (32.81 to 116.78) | 0.0175 (-0.0294 to 0.0644) |
| Italy | 52475 (26882 to 94918) | 74.46 (38.18 to 133.68) |  | 63320 (32654 to 113291) | 74.71 (38.33 to 134.16) | 0.0099 (-0.0176 to 0.0374) |
| Jamaica | 2428 (1265 to 4314) | 123.42 (64.19 to 218.67) |  | 3744 (1949 to 6628) | 122.96 (64.16 to 217.35) | -0.0133 (-0.015 to -0.0115) |
| Japan | 69402 (34901 to 125940) | 45.55 (23.19 to 82.7) |  | 86439 (44316 to 155860) | 45.89 (23.33 to 83.33) | 0.0062 (-0.0406 to 0.053) |
| Jordan | 2336 (1205 to 4236) | 93.33 (48.18 to 168.63) |  | 9660 (5019 to 17570) | 93.09 (48.03 to 168.08) | -0.0064 (-0.0099 to -0.003) |
| Kazakhstan | 12119 (6209 to 21768) | 80.75 (41.46 to 146.42) |  | 15405 (7881 to 27885) | 80.7 (41.32 to 145.69) | -0.0023 (-0.0032 to -0.0014) |
| Kenya | 11981 (6245 to 21096) | 87.83 (45.08 to 157.47) |  | 32680 (16981 to 57889) | 88.02 (45.12 to 158.02) | 0.0081 (0.0063 to 0.01) |
| Kiribati | 23 (12 to 41) | 40.68 (20.84 to 74.37) |  | 41 (21 to 74) | 40.72 (20.81 to 74.69) | 0.0041 (0.0022 to 0.006) |
| Kuwait | 1443 (742 to 2582) | 91.82 (47.25 to 166.28) |  | 4740 (2447 to 8461) | 92.75 (48.03 to 168.06) | 0.0385 (0.0327 to 0.0443) |
| Kyrgyzstan | 2915 (1502 to 5274) | 80.9 (41.31 to 146.38) |  | 4824 (2479 to 8693) | 81.01 (41.34 to 146.37) | 0.0063 (0.0038 to 0.0087) |
| Lao People's Democratic Republic | 1197 (612 to 2180) | 40.81 (20.8 to 75.02) |  | 2602 (1327 to 4736) | 40.85 (20.91 to 74.8) | 0.0041 (0.0004 to 0.0079) |
| Latvia | 2691 (1374 to 4829) | 86.83 (44.53 to 154.59) |  | 2250 (1149 to 3988) | 87.02 (44.59 to 155.7) | 0.0066 (0.0037 to 0.0095) |
| Lebanon | 2527 (1305 to 4592) | 93.61 (48.59 to 169.56) |  | 5089 (2624 to 9189) | 93.84 (48.6 to 169.69) | 0.0079 (0.0032 to 0.0127) |
| Lesotho | 1094 (564 to 1936) | 84.77 (43.85 to 150.83) |  | 1523 (793 to 2685) | 83.97 (43.64 to 149.43) | -0.0326 (-0.036 to -0.0291) |
| Liberia | 1139 (590 to 2022) | 83.88 (43.46 to 149.45) |  | 3000 (1537 to 5322) | 84.12 (43.73 to 149.83) | 0.0091 (0.0068 to 0.0115) |
| Libya | 2700 (1406 to 4858) | 92.83 (48.07 to 167.52) |  | 6757 (3479 to 12059) | 93.07 (48.21 to 168.06) | 0.0091 (0.0069 to 0.0113) |
| Lithuania | 3771 (1935 to 6792) | 91.58 (46.91 to 163.94) |  | 3458 (1763 to 6189) | 91.96 (47.19 to 165.83) | 0.0141 (0.0114 to 0.0168) |
| Luxembourg | 283 (143 to 514) | 60.17 (30.61 to 109) |  | 477 (241 to 865) | 60 (30.51 to 109.14) | -0.0099 (-0.012 to -0.0078) |
| Madagascar | 6534 (3377 to 11538) | 84.56 (43.8 to 151.31) |  | 16119 (8402 to 28544) | 84.91 (43.73 to 151.91) | 0.0148 (0.0102 to 0.0195) |
| Malawi | 5160 (2664 to 9085) | 84.46 (43.57 to 150.92) |  | 10429 (5403 to 18601) | 84.8 (43.98 to 151.79) | 0.0173 (0.0136 to 0.021) |
| Malaysia | 5750 (2937 to 10414) | 40.76 (20.83 to 74.53) |  | 13119 (6671 to 24326) | 40.77 (20.94 to 75.49) | 0.0007 (-0.0036 to 0.005) |
| Maldives | 58 (30 to 106) | 40.62 (20.74 to 74.78) |  | 215 (110 to 392) | 40.6 (20.61 to 74.48) | -0.0016 (-0.0065 to 0.0034) |
| Mali | 4808 (2496 to 8497) | 84.63 (43.98 to 151.04) |  | 11571 (5955 to 20543) | 84.97 (43.95 to 152.49) | 0.0165 (0.0112 to 0.0217) |
| Malta | 248 (126 to 451) | 60.29 (30.74 to 109.66) |  | 358 (182 to 657) | 60.1 (30.69 to 109.14) | -0.0111 (-0.0121 to -0.0101) |
| Marshall Islands | 12 (6 to 21) | 40.64 (20.83 to 74.46) |  | 20 (10 to 37) | 40.43 (20.77 to 74.34) | -0.0197 (-0.0228 to -0.0165) |
| Mauritania | 1179 (609 to 2090) | 85.08 (44.32 to 152.54) |  | 2511 (1300 to 4422) | 85.2 (44.23 to 152.01) | 0.0052 (0.0031 to 0.0073) |
| Mauritius | 412 (211 to 745) | 40.76 (20.82 to 74.55) |  | 635 (323 to 1175) | 40.66 (20.69 to 74.71) | -0.0069 (-0.0104 to -0.0034) |
| Mexico | 79085 (41580 to 140200) | 122.58 (64.04 to 220.33) |  | 158059 (82573 to 284214) | 122.74 (64.16 to 220.76) | 0.003 (-0.0009 to 0.0068) |
| Micronesia (Federated States of) | 29 (15 to 52) | 40.7 (20.74 to 74.06) |  | 38 (19 to 68) | 40.66 (20.78 to 74.38) | -0.0042 (-0.0084 to -0.0001) |
| Monaco | 26 (13 to 47) | 60.4 (30.94 to 109.73) |  | 32 (16 to 58) | 60.22 (30.58 to 108.75) | -0.0098 (-0.0116 to -0.008) |
| Mongolia | 1221 (627 to 2188) | 80.7 (41.09 to 147.03) |  | 2665 (1369 to 4807) | 80.89 (41.37 to 147.09) | 0.0079 (0.0063 to 0.0095) |
| Montenegro | 483 (247 to 874) | 74.52 (38.16 to 134.84) |  | 581 (296 to 1036) | 74.39 (38.05 to 134.22) | -0.0055 (-0.0075 to -0.0035) |
| Morocco | 18529 (9652 to 33565) | 93.75 (48.42 to 169.43) |  | 34361 (17572 to 62050) | 93.56 (48.18 to 169.11) | -0.0067 (-0.0082 to -0.0053) |
| Mozambique | 7300 (3794 to 12892) | 84.35 (43.93 to 150.66) |  | 15641 (8132 to 27858) | 84.46 (43.93 to 150.74) | 0.0027 (-0.0028 to 0.0082) |
| Myanmar | 13166 (6714 to 23981) | 40.76 (20.82 to 74.44) |  | 22185 (11328 to 40506) | 40.92 (21 to 74.95) | 0.0171 (0.0106 to 0.0236) |
| Namibia | 831 (430 to 1476) | 84.57 (43.88 to 151.18) |  | 1680 (864 to 2980) | 84.45 (43.85 to 151.24) | -0.0056 (-0.0089 to -0.0024) |
| Nauru | 3 (2 to 5) | 40.71 (20.62 to 75.28) |  | 3 (2 to 6) | 40.75 (20.76 to 74.28) | 0.0035 (-0.0004 to 0.0073) |
| Nepal | 13719 (7068 to 24910) | 99.78 (51.68 to 179.99) |  | 27540 (14141 to 49676) | 100.46 (51.93 to 181.31) | 0.0234 (0.0199 to 0.0269) |
| Netherlands | 7655 (3927 to 14067) | 43.42 (22.12 to 79.34) |  | 9781 (4973 to 17753) | 43.35 (22.02 to 78.99) | -0.0052 (-0.0174 to 0.0071) |
| New Zealand | 2718 (1384 to 4935) | 73.78 (37.46 to 133.75) |  | 4068 (2083 to 7385) | 74.01 (37.79 to 133.74) | 0.0113 (0.0061 to 0.0166) |
| Nicaragua | 3072 (1592 to 5472) | 122.77 (63.5 to 217.84) |  | 7372 (3823 to 13092) | 122.9 (64.02 to 217.16) | 0.0033 (0.0016 to 0.0049) |
| Niger | 4016 (2055 to 7066) | 84.86 (43.89 to 150.99) |  | 11049 (5713 to 19521) | 85.11 (44.32 to 152.15) | 0.0104 (0.0069 to 0.0139) |
| Nigeria | 54345 (28184 to 96653) | 87.66 (44.87 to 156.94) |  | 126595 (65893 to 224416) | 88.18 (45.28 to 158.46) | 0.0207 (0.0198 to 0.0216) |
| Niue | 1 (0 to 2) | 40.8 (20.93 to 73.98) |  | 1 (0 to 1) | 40.58 (20.91 to 74.63) | -0.0171 (-0.0204 to -0.0138) |
| North Macedonia | 1524 (781 to 2736) | 74.36 (38.13 to 133.6) |  | 2052 (1045 to 3689) | 74.33 (38.31 to 134.38) | -0.0007 (-0.0024 to 0.0011) |
| Northern Mariana Islands | 17 (9 to 31) | 40.58 (20.76 to 74.62) |  | 20 (10 to 38) | 40.66 (20.55 to 74.69) | 0.0105 (0.0072 to 0.0138) |
| Norway | 1962 (1004 to 3541) | 38.51 (19.56 to 68.99) |  | 2674 (1361 to 4880) | 39.1 (19.98 to 71.24) | 0.0593 (0.0456 to 0.0731) |
| Oman | 1313 (678 to 2345) | 91.61 (47.48 to 165.47) |  | 4423 (2240 to 7945) | 91.09 (46.6 to 165.1) | -0.0205 (-0.0557 to 0.0148) |
| Pakistan | 77727 (40635 to 138681) | 102.26 (52.82 to 183.61) |  | 171796 (90027 to 307018) | 102.28 (52.44 to 185.09) | 0.0008 (-0.0004 to 0.002) |
| Palau | 6 (3 to 10) | 40.71 (20.7 to 75.07) |  | 9 (4 to 16) | 40.41 (20.58 to 73.88) | -0.0327 (-0.0427 to -0.0226) |
| Palestine | 1227 (632 to 2244) | 93.81 (48.5 to 170.44) |  | 3675 (1908 to 6624) | 93.14 (48.33 to 168.18) | -0.0247 (-0.0259 to -0.0234) |
| Panama | 2461 (1285 to 4380) | 122.94 (63.98 to 218.6) |  | 5171 (2694 to 9152) | 122.93 (64.06 to 217.39) | -0.0009 (-0.0022 to 0.0005) |
| Papua New Guinea | 1212 (622 to 2210) | 40.47 (20.72 to 74.18) |  | 3187 (1631 to 5774) | 40.43 (20.54 to 73.6) | -0.0037 (-0.0101 to 0.0028) |
| Paraguay | 3843 (1981 to 6788) | 126.01 (65.35 to 223.25) |  | 8451 (4339 to 14962) | 125.91 (65.11 to 223.26) | -0.003 (-0.0049 to -0.0012) |
| Peru | 20775 (10836 to 36747) | 123.09 (64.02 to 217.93) |  | 42571 (22096 to 75320) | 123.26 (64.08 to 218.57) | 0.0053 (0.0041 to 0.0065) |
| Philippines | 19861 (10203 to 35464) | 42.61 (21.55 to 76.45) |  | 43119 (21910 to 76839) | 42.73 (21.68 to 76.63) | 0.0109 (0.0092 to 0.0125) |
| Poland | 43542 (22172 to 76935) | 104.9 (53.65 to 185.41) |  | 53924 (27443 to 95577) | 105.34 (53.64 to 185.83) | 0.0144 (0.0093 to 0.0195) |
| Portugal | 7601 (3848 to 13616) | 65.06 (33.03 to 117.02) |  | 9600 (4864 to 17185) | 65.16 (33.15 to 115.74) | 0.0069 (0.0019 to 0.0119) |
| Puerto Rico | 4452 (2322 to 7890) | 123.13 (64.08 to 219.2) |  | 5565 (2866 to 9898) | 122.96 (63.89 to 217.75) | -0.0057 (-0.0082 to -0.0031) |
| Qatar | 389 (198 to 705) | 90 (46.55 to 163.01) |  | 3055 (1553 to 5480) | 88.75 (45.71 to 160.37) | -0.0485 (-0.0538 to -0.0431) |
| Republic of Korea | 22227 (11306 to 39776) | 52.4 (26.58 to 94.47) |  | 38379 (19211 to 69923) | 52.51 (26.67 to 94.45) | 0.0999 (-0.1971 to 0.3978) |
| Republic of Moldova | 3959 (2020 to 7057) | 86.87 (44.27 to 154.64) |  | 4163 (2101 to 7445) | 86.99 (44.31 to 154.88) | 0.0048 (0.0037 to 0.006) |
| Romania | 19110 (9754 to 34543) | 74.28 (38.04 to 133.57) |  | 19246 (9834 to 34835) | 74.51 (38.09 to 135) | 0.0116 (0.0094 to 0.0138) |
| Russian Federation | 146386 (73979 to 266746) | 85.93 (43.68 to 156.68) |  | 161943 (81853 to 293186) | 86.19 (43.95 to 157.49) | 0.0589 (-0.1085 to 0.2266) |
| Rwanda | 3824 (1962 to 6795) | 84.57 (43.85 to 150.43) |  | 8138 (4207 to 14379) | 84.81 (43.99 to 152.08) | 0.0067 (-0.0024 to 0.0158) |
| Saint Kitts and Nevis | 44 (23 to 78) | 122.92 (63.97 to 218.51) |  | 85 (44 to 151) | 122.76 (63.87 to 217.32) | -0.0039 (-0.0057 to -0.0022) |
| Saint Lucia | 132 (69 to 233) | 122.8 (63.76 to 217.12) |  | 253 (131 to 448) | 122.59 (63.82 to 216.58) | -0.0062 (-0.0083 to -0.0042) |
| Saint Vincent and the Grenadines | 106 (55 to 188) | 123.01 (64.03 to 218.03) |  | 155 (80 to 275) | 122.64 (63.87 to 217.49) | -0.0104 (-0.0123 to -0.0085) |
| Samoa | 49 (25 to 89) | 40.78 (20.83 to 74.14) |  | 73 (37 to 134) | 40.68 (20.66 to 74.96) | -0.0086 (-0.0137 to -0.0036) |
| San Marino | 17 (8 to 30) | 60.27 (30.65 to 108.89) |  | 26 (13 to 47) | 60.33 (30.73 to 109.56) | 0.0037 (0.0028 to 0.0047) |
| Sao Tome and Principe | 67 (35 to 119) | 85.2 (43.93 to 152.38) |  | 139 (72 to 244) | 85.05 (44 to 151.63) | -0.0062 (-0.0069 to -0.0055) |
| Saudi Arabia | 10769 (5599 to 19122) | 91.66 (47.67 to 164.85) |  | 36198 (18614 to 64357) | 91.6 (47.6 to 164.95) | -0.0006 (-0.003 to 0.0018) |
| Senegal | 4046 (2100 to 7164) | 84.76 (44.06 to 151.11) |  | 9366 (4867 to 16521) | 84.91 (44.04 to 151.92) | 0.008 (0.0065 to 0.0095) |
| Serbia | 7957 (4030 to 14373) | 74.42 (37.99 to 134.27) |  | 8453 (4314 to 15354) | 74.33 (38.08 to 134.55) | -0.0045 (-0.0065 to -0.0025) |
| Seychelles | 26 (14 to 48) | 40.98 (20.91 to 74.97) |  | 47 (24 to 87) | 40.7 (20.85 to 74.85) | -0.0229 (-0.0262 to -0.0196) |
| Sierra Leone | 2182 (1126 to 3851) | 84.65 (43.7 to 151.23) |  | 5021 (2590 to 8916) | 84.85 (43.9 to 151.72) | 0.0065 (0.0006 to 0.0125) |
| Singapore | 1780 (913 to 3242) | 56.34 (28.66 to 102.24) |  | 4273 (2170 to 7691) | 56.94 (29.19 to 101.88) | 0.036 (0.0197 to 0.0523) |
| Slovakia | 4207 (2143 to 7573) | 74.27 (37.94 to 133.56) |  | 5348 (2720 to 9623) | 74.33 (38.13 to 134.35) | 0.0027 (0.0012 to 0.0041) |
| Slovenia | 1684 (859 to 3054) | 74.17 (38.05 to 133.45) |  | 2113 (1074 to 3769) | 74.25 (37.97 to 133.8) | 0.0032 (0 to 0.0065) |
| Solomon Islands | 90 (46 to 165) | 40.62 (20.58 to 74.58) |  | 204 (103 to 367) | 40.62 (20.59 to 74.24) | 0.0007 (-0.0031 to 0.0044) |
| Somalia | 3742 (1932 to 6624) | 84.5 (43.63 to 150.48) |  | 10599 (5516 to 18746) | 84.62 (43.81 to 150.69) | 0.0056 (0.0026 to 0.0087) |
| South Africa | 25535 (13362 to 45436) | 87.85 (45.08 to 157.3) |  | 48337 (24945 to 86613) | 87.46 (45.04 to 156.38) | -0.0141 (-0.0185 to -0.0097) |
| South Sudan | 3190 (1656 to 5626) | 83.7 (43.25 to 148.63) |  | 5143 (2661 to 9063) | 84.09 (43.5 to 149.57) | 0.0152 (0.011 to 0.0193) |
| Spain | 24273 (12419 to 44027) | 54.12 (27.58 to 97.51) |  | 33772 (17181 to 61275) | 54.06 (27.5 to 97.4) | 0.0857 (-0.1205 to 0.2923) |
| Sri Lanka | 6167 (3167 to 11224) | 40.77 (20.93 to 74.66) |  | 9871 (5035 to 18133) | 40.85 (20.85 to 74.64) | 0.0069 (0.005 to 0.0087) |
| Sudan | 13036 (6775 to 23334) | 93.59 (48.14 to 169.19) |  | 29515 (15376 to 53081) | 93.45 (48.38 to 169.37) | -0.0056 (-0.0085 to -0.0026) |
| Suriname | 407 (213 to 717) | 122.87 (63.86 to 216.91) |  | 746 (387 to 1319) | 122.52 (63.63 to 216.27) | -0.0104 (-0.0122 to -0.0086) |
| Sweden | 5498 (2827 to 9949) | 51.64 (26.24 to 93.44) |  | 7274 (3747 to 13263) | 55 (28.02 to 99.92) | 0.2112 (0.1758 to 0.2466) |
| Switzerland | 3034 (1556 to 5538) | 35.78 (18.47 to 64.94) |  | 4207 (2148 to 7672) | 35.84 (18.35 to 65.01) | 0.0534 (-0.0379 to 0.1448) |
| Syrian Arab Republic | 7790 (4001 to 13919) | 93.57 (48.08 to 168.91) |  | 13006 (6661 to 23437) | 93.73 (48.28 to 169.47) | 0.006 (0.0015 to 0.0105) |
| Taiwan (Province of China) | 7779 (4017 to 14229) | 38.54 (19.7 to 70.34) |  | 12500 (6304 to 22638) | 40.02 (20.29 to 71.77) | 0.1863 (0.0452 to 0.3276) |
| Tajikistan | 3027 (1557 to 5454) | 80.92 (41.25 to 146.13) |  | 6466 (3305 to 11580) | 80.75 (41.31 to 145.44) | -0.0083 (-0.0101 to -0.0065) |
| Thailand | 20996 (10652 to 38209) | 40.84 (20.8 to 74.88) |  | 36608 (18724 to 67472) | 41.01 (21.03 to 75.29) | 0.0158 (0.0134 to 0.0183) |
| Timor-Leste | 225 (115 to 407) | 40.61 (20.73 to 73.98) |  | 424 (218 to 780) | 40.71 (20.8 to 74.91) | 0.0016 (-0.0256 to 0.0287) |
| Togo | 1866 (961 to 3346) | 84.96 (43.95 to 151.48) |  | 5057 (2616 to 8920) | 85.21 (44.05 to 151.9) | 0.0098 (0.008 to 0.0116) |
| Tokelau | 1 (0 to 1) | 40.96 (20.86 to 74.33) |  | 1 (0 to 1) | 40.68 (20.68 to 74.66) | -0.0216 (-0.0335 to -0.0098) |
| Tonga | 29 (15 to 53) | 40.89 (20.8 to 74.37) |  | 36 (18 to 66) | 40.82 (20.78 to 74.4) | -0.0055 (-0.0089 to -0.0022) |
| Trinidad and Tobago | 1305 (680 to 2317) | 122.89 (64.09 to 217.88) |  | 2028 (1049 to 3596) | 122.68 (63.84 to 217.85) | -0.0054 (-0.0074 to -0.0034) |
| Tunisia | 6347 (3283 to 11595) | 93.88 (48.29 to 168.94) |  | 12093 (6217 to 21913) | 93.89 (48.29 to 169.17) | 0.0006 (-0.0017 to 0.0029) |
| Turkey | 50790 (26301 to 91262) | 102.95 (53.05 to 185.39) |  | 102059 (53178 to 184472) | 109.67 (57.2 to 198.02) | 0.2016 (0.153 to 0.2502) |
| Turkmenistan | 2189 (1130 to 3930) | 81.02 (41.34 to 146.66) |  | 3946 (2025 to 7163) | 80.93 (41.25 to 146.86) | -0.0034 (-0.0057 to -0.0011) |
| Tuvalu | 3 (2 to 6) | 41.01 (20.83 to 75.15) |  | 5 (2 to 8) | 40.65 (20.79 to 73.97) | -0.0314 (-0.0337 to -0.0291) |
| Uganda | 8685 (4530 to 15366) | 84.11 (43.73 to 149.93) |  | 21461 (11110 to 38038) | 84.93 (44.11 to 151.64) | 0.0339 (0.0315 to 0.0363) |
| Ukraine | 55259 (28242 to 99698) | 89.61 (45.91 to 162.41) |  | 52738 (26966 to 94423) | 89.79 (46.08 to 163.81) | 0.0076 (0.0046 to 0.0107) |
| United Arab Emirates | 1545 (781 to 2778) | 90.29 (46.71 to 163.88) |  | 10704 (5441 to 19417) | 89.59 (46.3 to 161.86) | -0.0228 (-0.0316 to -0.014) |
| United Kingdom | 55288 (28283 to 100358) | 79.81 (41.09 to 143.87) |  | 68024 (34406 to 122609) | 79.31 (40.78 to 142.97) | 0.0619 (-0.1554 to 0.2798) |
| United Republic of Tanzania | 13776 (7133 to 24288) | 84.37 (43.81 to 150.23) |  | 33001 (17013 to 58534) | 84.85 (43.98 to 151.18) | 0.0219 (0.0187 to 0.0251) |
| United States of America | 239780 (123302 to 428321) | 83.9 (43.19 to 150.27) |  | 299369 (150725 to 531682) | 73.5 (37.17 to 131.47) | -0.5058 (-0.6064 to -0.4051) |
| United States Virgin Islands | 127 (65 to 224) | 123.5 (64.22 to 219.79) |  | 158 (81 to 280) | 123.2 (63.97 to 218.49) | -0.0075 (-0.0087 to -0.0063) |
| Uruguay | 3505 (1801 to 6299) | 105.28 (54.17 to 189.52) |  | 4242 (2195 to 7597) | 105.19 (54.32 to 187.87) | -0.0024 (-0.0039 to -0.001) |
| Uzbekistan | 12371 (6400 to 22352) | 80.88 (41.57 to 147.2) |  | 25080 (12805 to 44984) | 80.83 (41.4 to 145.85) | -0.0013 (-0.0036 to 0.001) |
| Vanuatu | 43 (22 to 76) | 40.57 (20.68 to 73.95) |  | 96 (49 to 175) | 40.59 (20.78 to 73.86) | 0.0016 (-0.004 to 0.0073) |
| Venezuela (Bolivarian Republic of) | 18317 (9488 to 32590) | 122.94 (64.14 to 217.04) |  | 36836 (19071 to 65023) | 123.03 (63.74 to 217.04) | 0.0023 (0.0006 to 0.004) |
| Viet Nam | 21701 (11183 to 39480) | 41.09 (21.08 to 75.13) |  | 43630 (22286 to 80462) | 41.08 (21.03 to 75.32) | 0.0005 (-0.0018 to 0.0028) |
| Yemen | 7602 (3894 to 13532) | 93.22 (48.09 to 168.01) |  | 21861 (11379 to 39147) | 93.27 (47.96 to 169.35) | 0.0019 (-0.0002 to 0.004) |
| Zambia | 4039 (2109 to 7111) | 84.49 (43.8 to 150.52) |  | 10452 (5355 to 18425) | 84.55 (43.64 to 150.77) | 0.0022 (-0.0011 to 0.0054) |
| Zimbabwe | 5451 (2821 to 9651) | 84.79 (43.84 to 152.1) |  | 9349 (4822 to 16441) | 84.63 (43.91 to 150.81) | -0.0057 (-0.0094 to -0.0021) |

UI: uncertainty interval, CI: confidence interval, AAPC: average annual percent change, YLDs: Years Lived with Disability.
