# Supplementary material for: Burden of gastroesophageal reflux disease in 204 countries and territories, 1990–2019: a systematic analysis for the Global Burden of disease study 2019
Source: BMC Public Health. 2023 Mar 29;23:582. doi: 10.1186/s12889-023-15272-z (PMC10053627; doi:10.1186/s12889-023-15272-z)
Supplement: Supplementary file 6 — Table S5. Changes in YLDs number according to population-level determinants and causes from 1990 to 2019. [file 12889_2023_15272_MOESM6_ESM.docx]

Table S5. Changes in YLDs number according to population-level determinants and causes from 1990 to 2019.

| Location | Overll difference ^a^ | Change due to Population-level determinants (% contribute to the total changes) | | |
| --- | --- | --- | --- | --- |
|  |  | Aging ^b^ | Population ^c^ | Epidemiological change ^d^ |
| Global | 2626125 | 636013.742 (24.22%) | 1855332.77 (70.65%) | 134778.34 (5.13%) |
| **Sex** |  |  |  |  |
| Female | 1376509 | 333414.142 (24.22%) | 979639.299 (71.17%) | 63455.802 (4.61%) |
| Male | 1249616 | 301890.633 (24.16%) | 876508.484 (70.14%) | 71216.491 (5.7%) |
| **SDI** |  |  |  |  |
| High SDI | 215831.6 | 76284.008 (35.34%) | 163985.618 (75.98%) | -24438.05 (-11.32%) |
| High-middle SDI | 390605.5 | 175829.689 (45.01%) | 250319.744 (64.09%) | -35543.927 (-9.1%) |
| Middle SDI | 863346.1 | 270688.637 (31.35%) | 487258.753 (56.44%) | 105398.661 (12.21%) |
| Low-middle SDI | 743888.2 | 180554.357 (24.27%) | 547116.159 (73.55%) | 16217.654 (2.18%) |
| Low SDI | 410767.2 | 13000.743 (3.16%) | 398005.399 (96.89%) | -238.914 (-0.06%) |
| **Region** |  |  |  |  |
| High-income Asia Pacific | 35810.51 | 22496.765 (62.82%) | 10797.035 (30.15%) | 2516.712 (7.03%) |
| High-income North America | 68546.39 | 22679.215 (33.09%) | 81636.663 (119.1%) | -35769.492 (-52.18%) |
| Western Europe | 72917.89 | 28568.872 (39.18%) | 44690.002 (61.29%) | -340.983 (-0.47%) |
| Australasia | 9015.785 | 1933.759 (21.45%) | 7100.373 (78.75%) | -18.347 (-0.2%) |
| Andean Latin America | 40911.86 | 10868.241 (26.57%) | 29983.959 (73.29%) | 59.656 (0.15%) |
| Tropical Latin America | 148900.6 | 53118.439 (35.67%) | 96811.026 (65.02%) | -1028.913 (-0.69%) |
| Central Latin America | 160336.1 | 52486.65 (32.74%) | 107470.836 (67.03%) | 378.619 (0.24%) |
| Southern Latin America | 28418.43 | 7469.275 (26.28%) | 21007.944 (73.92%) | -58.788 (-0.21%) |
| Caribbean | 23189.55 | 7207.881 (31.08%) | 16066.78 (69.28%) | -85.112 (-0.37%) |
| Central Europe | 15218.75 | 19520.267 (128.26%) | -5977.438 (-39.28%) | 1675.918 (11.01%) |
| Eastern Europe | 12914.48 | 25821.776 (199.94%) | -13203.528 (-102.24%) | 296.235 (2.29%) |
| Central Asia | 27813.99 | 7795.71 (28.03%) | 20048.726 (72.08%) | -30.45 (-0.11%) |
| North Africa and Middle East | 319831.4 | 80066.834 (25.03%) | 236922.506 (74.08%) | 2842.05 (0.89%) |
| South Asia | 904322.2 | 208334.836 (23.04%) | 691266.493 (76.44%) | 4720.833 (0.52%) |
| Southeast Asia | 137803.4 | 46815.653 (33.97%) | 90606.89 (65.75%) | 380.899 (0.28%) |
| East Asia | 247983.1 | 130107.466 (52.47%) | 118679.095 (47.86%) | -803.478 (-0.32%) |
| Oceania | 2462.68 | 271.015 (11%) | 2194.187 (89.1%) | -2.522 (-0.1%) |
| Western Sub-Saharan Africa | 156911.6 | -1958.44 (-1.25%) | 158202.369 (100.82%) | 667.706 (0.43%) |
| Eastern Sub-Saharan Africa | 137660.3 | 7057.481 (5.13%) | 129908.973 (94.37%) | 693.883 (0.5%) |
| Central Sub-Saharan Africa | 45774.85 | 751.526 (1.64%) | 44762.691 (97.79%) | 260.63 (0.57%) |
| Southern Sub-Saharan Africa | 29381 | 8763.832 (29.83%) | 20782.871 (70.74%) | -165.708 (-0.56%) |
| **Nation** |  |  |  |  |
| Afghanistan | 16015.78 | -1004.873 (-6.27%) | 17073.952 (106.61%) | -53.297 (-0.33%) |
| Albania | 301.533 | 503.116 (166.85%) | -204.649 (-67.87%) | 3.066 (1.02%) |
| Algeria | 22453.6 | 7733.224 (34.44%) | 14753.055 (65.7%) | -32.678 (-0.15%) |
| American Samoa | 6.102 | 2.457 (40.27%) | 3.697 (60.59%) | -0.052 (-0.85%) |
| Andorra | 30.567 | 7.569 (24.76%) | 22.955 (75.1%) | 0.043 (0.14%) |
| Angola | 10853.91 | -327.297 (-3.02%) | 11122.821 (102.48%) | 58.382 (0.54%) |
| Antigua and Barbuda | 57.465 | 16.85 (29.32%) | 40.863 (71.11%) | -0.248 (-0.43%) |
| Argentina | 18141.17 | 3946.466 (21.75%) | 14236.937 (78.48%) | -42.23 (-0.23%) |
| Armenia | 355.253 | 555.82 (156.46%) | -207.372 (-58.37%) | 6.805 (1.92%) |
| Australia | 7665.669 | 1567.04 (20.44%) | 6095.36 (79.52%) | 3.269 (0.04%) |
| Austria | 1944.575 | 741.499 (38.13%) | 1225.167 (63%) | -22.091 (-1.14%) |
| Azerbaijan | 4045.636 | 1335.019 (33%) | 2721.844 (67.28%) | -11.227 (-0.28%) |
| Bahamas | 240.198 | 75.11 (31.27%) | 165.663 (68.97%) | -0.575 (-0.24%) |
| Bahrain | 1183.344 | 213.033 (18%) | 975.383 (82.43%) | -5.072 (-0.43%) |
| Bangladesh | 82532.54 | 30583.218 (37.06%) | 50763.996 (61.51%) | 1185.327 (1.44%) |
| Barbados | 133.15 | 60.017 (45.07%) | 74.247 (55.76%) | -1.114 (-0.84%) |
| Belarus | 478.622 | 1289.387 (269.4%) | -818.295 (-170.97%) | 7.53 (1.57%) |
| Belgium | 1791.664 | 486.221 (27.14%) | 1235.854 (68.98%) | 69.589 (3.88%) |
| Belize | 303.985 | 61.631 (20.27%) | 242.787 (79.87%) | -0.433 (-0.14%) |
| Benin | 4480.068 | 144.713 (3.23%) | 4323.75 (96.51%) | 11.605 (0.26%) |
| Bermuda | 22.389 | 12.274 (54.82%) | 10.201 (45.56%) | -0.086 (-0.38%) |
| Bhutan | 319.687 | 156.988 (49.11%) | 161.526 (50.53%) | 1.173 (0.37%) |
| Bolivia (Plurinational State of) | 7638.839 | 1606.899 (21.04%) | 6014.467 (78.74%) | 17.473 (0.23%) |
| Bosnia and Herzegovina | -226.453 | 750.715 (-331.51%) | -967.753 (427.35%) | -9.415 (4.16%) |
| Botswana | 1060.097 | 312.914 (29.52%) | 755.91 (71.31%) | -8.727 (-0.82%) |
| Brazil | 144293.1 | 52136.714 (36.13%) | 93191.164 (64.58%) | -1034.803 (-0.72%) |
| Brunei Darussalam | 128.263 | 34.898 (27.21%) | 93.036 (72.54%) | 0.329 (0.26%) |
| Bulgaria | -581.215 | 960.54 (-165.26%) | -1554.033 (267.38%) | 12.278 (-2.11%) |
| Burkina Faso | 7586.147 | 249.516 (3.29%) | 7289.474 (96.09%) | 47.157 (0.62%) |
| Burundi | 3597.337 | -4.081 (-0.11%) | 3611.557 (100.4%) | -10.139 (-0.28%) |
| Cabo Verde | 251.202 | 79.479 (31.64%) | 173.484 (69.06%) | -1.761 (-0.7%) |
| Cambodia | 3467.562 | 1047.745 (30.22%) | 2416.212 (69.68%) | 3.605 (0.1%) |
| Cameroon | 11673.78 | 324.517 (2.78%) | 11311.235 (96.89%) | 38.023 (0.33%) |
| Canada | 8951.374 | 2335.412 (26.09%) | 6654.618 (74.34%) | -38.656 (-0.43%) |
| Central African Republic | 1560.569 | 12 (0.77%) | 1541.88 (98.8%) | 6.689 (0.43%) |
| Chad | 4751.928 | -550.625 (-11.59%) | 5310.534 (111.76%) | -7.981 (-0.17%) |
| Chile | 9538.607 | 3259.863 (34.18%) | 6284.039 (65.88%) | -5.295 (-0.06%) |
| China | 239253.7 | 126356.248 (52.81%) | 114096.578 (47.69%) | -1199.131 (-0.5%) |
| Colombia | 31042.92 | 10538.189 (33.95%) | 20388.685 (65.68%) | 116.043 (0.37%) |
| Comoros | 273.873 | 81.98 (29.93%) | 190.852 (69.69%) | 1.041 (0.38%) |
| Congo | 2185.049 | 333.719 (15.27%) | 1847.906 (84.57%) | 3.424 (0.16%) |
| Cook Islands | 1.779 | 1.776 (99.83%) | -0.002 (-0.11%) | 0.005 (0.28%) |
| Costa Rica | 3353.102 | 1069.195 (31.89%) | 2282.626 (68.08%) | 1.281 (0.04%) |
| Croatia | 1.315 | 539.313 (41012.4%) | -536.715 (-40814.83%) | -1.283 (-97.57%) |
| Cuba | 4056.003 | 2774.9 (68.41%) | 1299.561 (32.04%) | -18.458 (-0.46%) |
| Cyprus | 512.51 | 117.499 (22.93%) | 395.044 (77.08%) | -0.033 (-0.01%) |
| Czechia | 1816.324 | 1419.398 (78.15%) | 418.569 (23.04%) | -21.643 (-1.19%) |
| C么te d'Ivoire | 9625.573 | 1107.581 (11.51%) | 8446.667 (87.75%) | 71.325 (0.74%) |
| Democratic People's Republic of Korea | 4008.197 | 1373.146 (34.26%) | 2684.617 (66.98%) | -49.566 (-1.24%) |
| Democratic Republic of the Congo | 29930.26 | 712.001 (2.38%) | 29033.872 (97.01%) | 184.39 (0.62%) |
| Denmark | 871.799 | 260.54 (29.89%) | 634.017 (72.73%) | -22.758 (-2.61%) |
| Djibouti | 606.236 | 127.013 (20.95%) | 478.051 (78.86%) | 1.172 (0.19%) |
| Dominica | 14.875 | 16.966 (114.06%) | -1.71 (-11.5%) | -0.381 (-2.56%) |
| Dominican Republic | 6315.137 | 1881.905 (29.8%) | 4457.831 (70.59%) | -24.599 (-0.39%) |
| Ecuador | 11477.14 | 2707.785 (23.59%) | 8770.629 (76.42%) | -1.272 (-0.01%) |
| Egypt | 42693.81 | 5925.953 (13.88%) | 36807.967 (86.21%) | -40.115 (-0.09%) |
| El Salvador | 2750.623 | 1341.146 (48.76%) | 1396.413 (50.77%) | 13.064 (0.47%) |
| Equatorial Guinea | 585.439 | -18.79 (-3.21%) | 602.51 (102.92%) | 1.719 (0.29%) |
| Eritrea | 2549.08 | 282.724 (11.09%) | 2255.151 (88.47%) | 11.205 (0.44%) |
| Estonia | -50.796 | 186.007 (-366.18%) | -236.375 (465.34%) | -0.428 (0.84%) |
| Eswatini | 344.625 | 115.274 (33.45%) | 236.132 (68.52%) | -6.781 (-1.97%) |
| Ethiopia | 35084.78 | 1377.676 (3.93%) | 33515.438 (95.53%) | 191.665 (0.55%) |
| Fiji | 114.545 | 51.533 (44.99%) | 63.806 (55.7%) | -0.794 (-0.69%) |
| Finland | 939.08 | 460.308 (49.02%) | 624.93 (66.55%) | -146.158 (-15.56%) |
| France | 9303.401 | 3244.853 (34.88%) | 5993.506 (64.42%) | 65.042 (0.7%) |
| Gabon | 659.619 | 96.836 (14.68%) | 561.268 (85.09%) | 1.515 (0.23%) |
| Gambia | 809.334 | 54.251 (6.7%) | 753.715 (93.13%) | 1.368 (0.17%) |
| Georgia | -1133.45 | 538.43 (-47.5%) | -1650.658 (145.63%) | -21.217 (1.87%) |
| Germany | 10233.53 | 4760.411 (46.52%) | 4508.22 (44.05%) | 964.895 (9.43%) |
| Ghana | 12977.02 | 1795.742 (13.84%) | 11121.276 (85.7%) | 60.005 (0.46%) |
| Greece | 1381.287 | 1335.715 (96.7%) | 52.525 (3.8%) | -6.953 (-0.5%) |
| Greenland | 6.788 | 5.08 (74.84%) | 1.56 (22.98%) | 0.148 (2.18%) |
| Grenada | 53.421 | 26.254 (49.15%) | 27.552 (51.58%) | -0.385 (-0.72%) |
| Guam | 22.757 | 7.648 (33.61%) | 15.128 (66.48%) | -0.019 (-0.08%) |
| Guatemala | 12187.83 | 1889.491 (15.5%) | 10259.353 (84.18%) | 38.984 (0.32%) |
| Guinea | 3514.787 | -376.451 (-10.71%) | 3877.868 (110.33%) | 13.37 (0.38%) |
| Guinea-Bissau | 579.812 | 43.399 (7.49%) | 535.361 (92.33%) | 1.052 (0.18%) |
| Guyana | 185.4 | 145.727 (78.6%) | 39.497 (21.3%) | 0.176 (0.09%) |
| Haiti | 7267.989 | 939.786 (12.93%) | 6330.245 (87.1%) | -2.042 (-0.03%) |
| Honduras | 6498.851 | 1299.397 (19.99%) | 5194.746 (79.93%) | 4.708 (0.07%) |
| Hungary | 696.962 | 1241.226 (178.09%) | -570.003 (-81.78%) | 25.739 (3.69%) |
| Iceland | 75.504 | 20.41 (27.03%) | 53.17 (70.42%) | 1.924 (2.55%) |
| India | 713579.1 | 168816.23 (23.66%) | 540970.102 (75.81%) | 3792.786 (0.53%) |
| Indonesia | 51264.04 | 17900.281 (34.92%) | 33180.645 (64.72%) | 183.117 (0.36%) |
| Iran (Islamic Republic of) | 45136.32 | 20516.199 (45.45%) | 24436.944 (54.14%) | 183.181 (0.41%) |
| Iraq | 23085.92 | 3842.917 (16.65%) | 19199.719 (83.17%) | 43.288 (0.19%) |
| Ireland | 1350.954 | 431.507 (31.94%) | 921.163 (68.19%) | -1.716 (-0.13%) |
| Israel | 3220.665 | 422.357 (13.11%) | 2804.341 (87.07%) | -6.033 (-0.19%) |
| Italy | 10845.14 | 6553.93 (60.43%) | 4020.676 (37.07%) | 270.535 (2.49%) |
| Jamaica | 1316.1 | 628.807 (47.78%) | 698.377 (53.06%) | -11.084 (-0.84%) |
| Japan | 17037.33 | 13808.932 (81.05%) | 2417.323 (14.19%) | 811.075 (4.76%) |
| Jordan | 7324.154 | 1265.49 (17.28%) | 6072.673 (82.91%) | -14.009 (-0.19%) |
| Kazakhstan | 3286.758 | 1494.875 (45.48%) | 1796.991 (54.67%) | -5.108 (-0.16%) |
| Kenya | 20699.43 | 3420.14 (16.52%) | 17245.562 (83.31%) | 33.73 (0.16%) |
| Kiribati | 17.61 | 1.89 (10.73%) | 15.684 (89.06%) | 0.036 (0.2%) |
| Kuwait | 3297.178 | 575.777 (17.46%) | 2679.565 (81.27%) | 41.836 (1.27%) |
| Kyrgyzstan | 1909.43 | 347.785 (18.21%) | 1556.245 (81.5%) | 5.4 (0.28%) |
| Lao People's Democratic Republic | 1404.991 | 308.737 (21.97%) | 1094.774 (77.92%) | 1.48 (0.11%) |
| Latvia | -440.717 | 306.589 (-69.57%) | -753.249 (170.91%) | 5.943 (-1.35%) |
| Lebanon | 2562.581 | 673.217 (26.27%) | 1880.019 (73.36%) | 9.345 (0.36%) |
| Lesotho | 429.482 | 184.597 (42.98%) | 256.59 (59.74%) | -11.705 (-2.73%) |
| Liberia | 1861.641 | 65.519 (3.52%) | 1789.268 (96.11%) | 6.854 (0.37%) |
| Libya | 4056.83 | 1497.881 (36.92%) | 2542.661 (62.68%) | 16.288 (0.4%) |
| Lithuania | -313.158 | 562.245 (-179.54%) | -889.109 (283.92%) | 13.706 (-4.38%) |
| Luxembourg | 194.69 | 13.445 (6.91%) | 182.597 (93.79%) | -1.352 (-0.69%) |
| Madagascar | 9585.644 | 585.83 (6.11%) | 8958.28 (93.46%) | 41.534 (0.43%) |
| Malawi | 5268.992 | -126.068 (-2.39%) | 5363.548 (101.79%) | 31.512 (0.6%) |
| Malaysia | 7368.199 | 1752.114 (23.78%) | 5612.998 (76.18%) | 3.087 (0.04%) |
| Maldives | 156.576 | 47.472 (30.32%) | 109.6 (70%) | -0.496 (-0.32%) |
| Mali | 6763.763 | -574.061 (-8.49%) | 7307.218 (108.03%) | 30.606 (0.45%) |
| Malta | 110.45 | 52.11 (47.18%) | 59.404 (53.78%) | -1.064 (-0.96%) |
| Marshall Islands | 8.846 | 4.441 (50.2%) | 4.466 (50.49%) | -0.061 (-0.69%) |
| Mauritania | 1331.358 | 52.105 (3.91%) | 1276.264 (95.86%) | 2.989 (0.22%) |
| Mauritius | 222.955 | 122.298 (54.85%) | 101.889 (45.7%) | -1.232 (-0.55%) |
| Mexico | 78974.14 | 28933.277 (36.64%) | 49935.539 (63.23%) | 105.321 (0.13%) |
| Micronesia (Federated States of) | 8.777 | 7.039 (80.2%) | 1.77 (20.17%) | -0.032 (-0.36%) |
| Monaco | 6.132 | 0.309 (5.04%) | 5.947 (96.98%) | -0.124 (-2.02%) |
| Mongolia | 1444.254 | 519.416 (35.96%) | 921.27 (63.79%) | 3.568 (0.25%) |
| Montenegro | 98.453 | 87.911 (89.29%) | 11.625 (11.81%) | -1.083 (-1.1%) |
| Morocco | 15832.08 | 5282.679 (33.37%) | 10590.77 (66.89%) | -41.365 (-0.26%) |
| Mozambique | 8341.097 | -697.645 (-8.36%) | 9031.537 (108.28%) | 7.205 (0.09%) |
| Myanmar | 9018.697 | 3073.805 (34.08%) | 5876.046 (65.15%) | 68.846 (0.76%) |
| Namibia | 848.237 | 162.955 (19.21%) | 686.824 (80.97%) | -1.542 (-0.18%) |
| Nauru | 0.417 | 0.152 (36.45%) | 0.264 (63.31%) | 0.001 (0.24%) |
| Nepal | 13821.03 | 3254.967 (23.55%) | 10428.344 (75.45%) | 137.718 (1%) |
| Netherlands | 2126.148 | 834.103 (39.23%) | 1314.872 (61.84%) | -22.827 (-1.07%) |
| New Zealand | 1350.116 | 363.708 (26.94%) | 975.608 (72.26%) | 10.8 (0.8%) |
| Nicaragua | 4299.561 | 1370.851 (31.88%) | 2921.297 (67.94%) | 7.413 (0.17%) |
| Niger | 7032.981 | -442.457 (-6.29%) | 7455.973 (106.01%) | 19.465 (0.28%) |
| Nigeria | 72250.44 | -4937.476 (-6.83%) | 76681.186 (106.13%) | 506.733 (0.7%) |
| Niue | -0.091 | 0.14 (-153.85%) | -0.226 (248.35%) | -0.005 (5.49%) |
| North Macedonia | 528.282 | 346.258 (65.54%) | 182.826 (34.61%) | -0.802 (-0.15%) |
| Northern Mariana Islands | 3.424 | 3.462 (101.11%) | -0.089 (-2.6%) | 0.051 (1.49%) |
| Norway | 712.064 | 120.348 (16.9%) | 554.744 (77.91%) | 36.972 (5.19%) |
| Oman | 3110.076 | 738.692 (23.75%) | 2385.91 (76.72%) | -14.526 (-0.47%) |
| Pakistan | 94069.79 | 7086.668 (7.53%) | 86908.787 (92.39%) | 74.334 (0.08%) |
| Palau | 3.127 | 1.673 (53.5%) | 1.508 (48.23%) | -0.054 (-1.73%) |
| Palestine | 2448.389 | 334.677 (13.67%) | 2126.077 (86.84%) | -12.365 (-0.51%) |
| Panama | 2710.193 | 584.571 (21.57%) | 2125.591 (78.43%) | 0.031 (0%) |
| Papua New Guinea | 1974.825 | 160.101 (8.11%) | 1814.8 (91.9%) | -0.076 (0%) |
| Paraguay | 4607.476 | 1081.891 (23.48%) | 3529.698 (76.61%) | -4.113 (-0.09%) |
| Peru | 21795.88 | 6621.96 (30.38%) | 15128.572 (69.41%) | 45.344 (0.21%) |
| Philippines | 23257.32 | 4885.148 (21%) | 18295.612 (78.67%) | 76.563 (0.33%) |
| Poland | 10381.23 | 8468.54 (81.58%) | 1697.155 (16.35%) | 215.535 (2.08%) |
| Portugal | 1999.048 | 1398.272 (69.95%) | 586.968 (29.36%) | 13.808 (0.69%) |
| Puerto Rico | 1113.195 | 977.755 (87.83%) | 144.13 (12.95%) | -8.69 (-0.78%) |
| Qatar | 2665.81 | 227.085 (8.52%) | 2451.866 (91.97%) | -13.141 (-0.49%) |
| Republic of Korea | 16151.73 | 9467.831 (58.62%) | 6627.116 (41.03%) | 56.779 (0.35%) |
| Republic of Moldova | 204.394 | 747.059 (365.5%) | -548.455 (-268.33%) | 5.79 (2.83%) |
| Romania | 135.791 | 3282.047 (2416.98%) | -3209.94 (-2363.88%) | 63.684 (46.9%) |
| Russian Federation | 15557.57 | 17160.609 (110.3%) | -2005.308 (-12.89%) | 402.267 (2.59%) |
| Rwanda | 4313.987 | 659.481 (15.29%) | 3638.715 (84.35%) | 15.791 (0.37%) |
| Saint Kitts and Nevis | 40.732 | 14.689 (36.06%) | 26.136 (64.17%) | -0.093 (-0.23%) |
| Saint Lucia | 120.836 | 59.872 (49.55%) | 61.276 (50.71%) | -0.312 (-0.26%) |
| Saint Vincent and the Grenadines | 49.035 | 38.332 (78.17%) | 11.085 (22.61%) | -0.382 (-0.78%) |
| Samoa | 23.875 | 6.981 (29.24%) | 17.002 (71.21%) | -0.108 (-0.45%) |
| San Marino | 9.256 | 1.934 (20.89%) | 7.324 (79.13%) | -0.002 (-0.02%) |
| Sao Tome and Principe | 71.532 | 13.718 (19.18%) | 57.952 (81.02%) | -0.138 (-0.19%) |
| Saudi Arabia | 25428.99 | 6724.193 (26.44%) | 18694.042 (73.51%) | 10.753 (0.04%) |
| Senegal | 5320.579 | 615.055 (11.56%) | 4695.222 (88.25%) | 10.302 (0.19%) |
| Serbia | 496.396 | 945.306 (190.43%) | -435.893 (-87.81%) | -13.017 (-2.62%) |
| Seychelles | 20.784 | 7.52 (36.18%) | 13.499 (64.95%) | -0.235 (-1.13%) |
| Sierra Leone | 2838.984 | -80.33 (-2.83%) | 2910.31 (102.51%) | 9.004 (0.32%) |
| Singapore | 2493.193 | 663.2 (26.6%) | 1799.91 (72.19%) | 30.083 (1.21%) |
| Slovakia | 1141.502 | 874.75 (76.63%) | 263.307 (23.07%) | 3.445 (0.3%) |
| Slovenia | 428.624 | 302.505 (70.58%) | 124.125 (28.96%) | 1.994 (0.47%) |
| Solomon Islands | 114.078 | 17.998 (15.78%) | 96.102 (84.24%) | -0.022 (-0.02%) |
| Somalia | 6856.654 | -22.903 (-0.33%) | 6872.728 (100.23%) | 6.829 (0.1%) |
| South Africa | 22801.13 | 7011.006 (30.75%) | 15955.653 (69.98%) | -165.525 (-0.73%) |
| South Sudan | 1952.64 | 8.434 (0.43%) | 1922.989 (98.48%) | 21.217 (1.09%) |
| Spain | 9498.929 | 4314.064 (45.42%) | 5220.896 (54.96%) | -36.031 (-0.38%) |
| Sri Lanka | 3703.821 | 1520.246 (41.05%) | 2167.399 (58.52%) | 16.176 (0.44%) |
| Sudan | 16479.27 | 1217.581 (7.39%) | 15282.592 (92.74%) | -20.902 (-0.13%) |
| Suriname | 338.694 | 97.292 (28.73%) | 242.899 (71.72%) | -1.497 (-0.44%) |
| Sweden | 1775.885 | 186.247 (10.49%) | 1152.957 (64.92%) | 436.681 (24.59%) |
| Switzerland | 1172.945 | 262.728 (22.4%) | 905.942 (77.24%) | 4.275 (0.36%) |
| Syrian Arab Republic | 5216.326 | 2833.989 (54.33%) | 2356.491 (45.18%) | 25.846 (0.5%) |
| Taiwan (Province of China) | 4721.191 | 2453.89 (51.98%) | 1851.951 (39.23%) | 415.35 (8.8%) |
| Tajikistan | 3439.32 | 630.079 (18.32%) | 2815.239 (81.85%) | -5.998 (-0.17%) |
| Thailand | 15611.85 | 8118.928 (52%) | 7379.865 (47.27%) | 113.061 (0.72%) |
| Timor-Leste | 199.515 | 12.727 (6.38%) | 185.746 (93.1%) | 1.042 (0.52%) |
| Togo | 3190.656 | 546.792 (17.14%) | 2635.202 (82.59%) | 8.662 (0.27%) |
| Tokelau | -0.009 | 0.059 (-655.56%) | -0.064 (711.11%) | -0.004 (44.44%) |
| Tonga | 6.888 | 4.433 (64.36%) | 2.498 (36.27%) | -0.043 (-0.62%) |
| Trinidad and Tobago | 722.739 | 402.995 (55.76%) | 322.598 (44.64%) | -2.854 (-0.39%) |
| Tunisia | 5746.111 | 2346.791 (40.84%) | 3397.812 (59.13%) | 1.508 (0.03%) |
| Turkey | 51269.35 | 18669.66 (36.41%) | 27932.682 (54.48%) | 4667.01 (9.1%) |
| Turkmenistan | 1757.871 | 648.916 (36.91%) | 1112.525 (63.29%) | -3.57 (-0.2%) |
| Tuvalu | 1.267 | 0.153 (12.08%) | 1.147 (90.53%) | -0.033 (-2.6%) |
| Uganda | 12775.41 | -213.452 (-1.67%) | 12844.329 (100.54%) | 144.531 (1.13%) |
| Ukraine | -2521.43 | 5745.976 (-227.89%) | -8391.546 (332.81%) | 124.138 (-4.92%) |
| United Arab Emirates | 9159.022 | 1395.119 (15.23%) | 7782.275 (84.97%) | -18.372 (-0.2%) |
| United Kingdom | 12735.92 | 3069.722 (24.1%) | 10195.668 (80.05%) | -529.468 (-4.16%) |
| United Republic of Tanzania | 19225.29 | 1262.032 (6.56%) | 17825.293 (92.72%) | 137.962 (0.72%) |
| United States of America | 59588.93 | 20108.595 (33.75%) | 74709.839 (125.38%) | -35229.507 (-59.12%) |
| United States Virgin Islands | 31.054 | 27.845 (89.67%) | 3.618 (11.65%) | -0.409 (-1.32%) |
| Uruguay | 736.732 | 309.394 (42%) | 431.179 (58.53%) | -3.841 (-0.52%) |
| Uzbekistan | 12708.91 | 3099.997 (24.39%) | 9607.286 (75.59%) | 1.624 (0.01%) |
| Vanuatu | 53.871 | 6.809 (12.64%) | 47.027 (87.3%) | 0.035 (0.06%) |
| Venezuela (Bolivarian Republic of) | 18518.89 | 6376.644 (34.43%) | 12121.564 (65.46%) | 20.685 (0.11%) |
| Viet Nam | 21929.55 | 8832.671 (40.28%) | 13095.188 (59.71%) | 1.695 (0.01%) |
| Yemen | 14259.12 | 2070.862 (14.52%) | 12178.38 (85.41%) | 9.876 (0.07%) |
| Zambia | 6413.205 | 536.977 (8.37%) | 5873.586 (91.59%) | 2.642 (0.04%) |
| Zimbabwe | 3897.42 | 961.542 (24.67%) | 2944.66 (75.55%) | -8.782 (-0.23%) |

a.Change in YLDs number between year 2019 and 1990

b.Change in YLDs number due to change in the age structure

c.Change in YLDs number due to change in population number

d.Change in YLDs number due to epidemiologic changes. Epidemiologic changes refer to the YLDs number change when age structure and population hold constant

YLDs: Years Lived with Disability; SDI: Socio-demographic index.
