# Supplementary material for: Burden of gastroesophageal reflux disease in 204 countries and territories, 1990–2019: a systematic analysis for the Global Burden of disease study 2019
Source: BMC Public Health. 2023 Mar 29;23:582. doi: 10.1186/s12889-023-15272-z (PMC10053627; doi:10.1186/s12889-023-15272-z)
Supplement: Supplementary file 7 — Table S6. Frontier YLDs, and effective difference by country or territory. [file 12889_2023_15272_MOESM7_ESM.docx]

Table S6: Frontier YLDs, and effective difference by country or territory.

| **Location** | **SDI** | **Age standardized YLDs** | **Frontier YLDs** | **Effective difference** | **Effective difference rank (Age standardized YLDs rank)** |
| --- | --- | --- | --- | --- | --- |
| Afghanistan | 0.343 | 92.21 (47.65 to 167.08) | 40.34 | 51.87 | 128 (148) |
| Albania | 0.681 | 50.67 (25.97 to 92.11) | 31.24 | 19.43 | 46 (41) |
| Algeria | 0.652 | 93.66 (48.63 to 169.01) | 31.37 | 62.29 | 160 (158) |
| American Samoa | 0.712 | 40.5 (20.66 to 74.2) | 31.25 | 9.25 | 29 (9) |
| Andorra | 0.894 | 60.04 (30.75 to 109.03) | 31.26 | 28.78 | 55 (50) |
| Angola | 0.47 | 84.83 (44.07 to 151.86) | 34.47 | 50.36 | 119 (112) |
| Antigua and Barbuda | 0.743 | 122.96 (63.84 to 217.33) | 31.31 | 91.65 | 193 (191) |
| Argentina | 0.708 | 105.14 (54.26 to 187.94) | 31.26 | 73.88 | 169 (168) |
| Armenia | 0.689 | 80.92 (41.33 to 147.23) | 31.23 | 49.69 | 115 (87) |
| Australia | 0.839 | 65.36 (33.27 to 118.9) | 31.27 | 34.09 | 64 (59) |
| Austria | 0.849 | 78.52 (40.18 to 140.19) | 31.26 | 47.26 | 101 (76) |
| Azerbaijan | 0.683 | 80.87 (41.37 to 146.52) | 31.24 | 49.63 | 113 (85) |
| Bahrain | 0.751 | 91.14 (46.92 to 164.49) | 31.29 | 59.85 | 148 (145) |
| Bangladesh | 0.483 | 100.18 (52.01 to 179.08) | 33.98 | 66.2 | 164 (162) |
| Barbados | 0.742 | 123.24 (64.16 to 218.35) | 31.27 | 91.97 | 201 (200) |
| Belarus | 0.745 | 87.13 (44.48 to 156.14) | 31.28 | 55.86 | 140 (135) |
| Belgium | 0.851 | 65.6 (33.14 to 117.89) | 31.25 | 34.35 | 65 (60) |
| Belize | 0.603 | 123 (63.83 to 218.39) | 32.8 | 90.21 | 180 (192) |
| Benin | 0.352 | 85.06 (43.87 to 152.23) | 40.24 | 44.81 | 98 (123) |
| Bermuda | 0.813 | 123.42 (64.09 to 218.38) | 31.25 | 92.17 | 202 (202) |
| Bhutan | 0.455 | 100.27 (51.81 to 182.11) | 35.18 | 65.09 | 163 (163) |
| Bolivia | 0.566 | 122.87 (63.88 to 218.07) | 33.39 | 89.47 | 175 (184) |
| Bosnia and Herzegovina | 0.718 | 74.24 (37.97 to 133.88) | 31.27 | 42.96 | 70 (64) |
| Botswana | 0.634 | 84.1 (43.77 to 150.21) | 31.74 | 52.37 | 130 (93) |
| Brazil | 0.64 | 124.74 (64.6 to 222.06) | 31.54 | 93.2 | 203 (203) |
| Brunei | 0.823 | 49.83 (25.58 to 90.21) | 31.27 | 18.56 | 45 (40) |
| Bulgaria | 0.764 | 74.47 (38.12 to 134.13) | 31.26 | 43.2 | 78 (72) |
| Burkina Faso | 0.257 | 85.21 (44.09 to 152) | 83.72 | 1.49 | 7 (129) |
| Burundi | 0.284 | 84.44 (43.59 to 150.92) | 40.65 | 43.79 | 81 (96) |
| C?te d'Ivoire | 0.408 | 84.61 (44.1 to 151.08) | 40.24 | 44.37 | 86 (102) |
| Cambodia | 0.469 | 40.92 (20.81 to 74.92) | 34.3 | 6.61 | 11 (31) |
| Cameroon | 0.49 | 84.82 (43.83 to 151.72) | 33.86 | 50.96 | 122 (111) |
| Canada | 0.873 | 55.3 (28.34 to 100.28) | 31.27 | 24.03 | 51 (46) |
| Cape Verde | 0.525 | 85.18 (43.99 to 152.87) | 33.42 | 51.76 | 126 (127) |
| Central African Republic | 0.274 | 84.43 (43.87 to 150.61) | 44.66 | 39.77 | 66 (95) |
| Chad | 0.238 | 84.71 (43.81 to 151.35) | 83.81 | 0.9 | 5 (108) |
| Chile | 0.759 | 105.03 (53.95 to 187.75) | 31.26 | 73.77 | 167 (167) |
| China | 0.686 | 34.94 (17.73 to 63.02) | 31.25 | 3.69 | 9 (1) |
| Colombia | 0.633 | 123.23 (63.88 to 218.22) | 31.82 | 91.41 | 187 (199) |
| Comoros | 0.455 | 85.1 (44.13 to 152.4) | 35.27 | 49.83 | 116 (124) |
| Congo (Brazzaville) | 0.568 | 84.62 (43.94 to 150.79) | 33.38 | 51.23 | 125 (103) |
| Cook Islands | 0.764 | 40.72 (20.95 to 74.13) | 31.28 | 9.44 | 35 (23) |
| Costa Rica | 0.68 | 123.1 (64.09 to 218.52) | 31.24 | 91.86 | 198 (197) |
| Croatia | 0.794 | 74.25 (37.85 to 133.59) | 31.28 | 42.97 | 71 (65) |
| Cuba | 0.668 | 122.85 (64.23 to 217.61) | 31.3 | 91.55 | 191 (183) |
| Cyprus | 0.841 | 60.23 (30.83 to 108.84) | 31.27 | 28.96 | 58 (54) |
| Denmark | 0.89 | 74.28 (38.37 to 132.3) | 31.27 | 43.01 | 73 (67) |
| Djibouti | 0.459 | 84.86 (43.81 to 150.7) | 34.73 | 50.13 | 118 (116) |
| Dominica | 0.729 | 122.64 (63.77 to 217.36) | 31.23 | 91.41 | 186 (177) |
| Dominican Republic | 0.592 | 123.03 (63.96 to 217.58) | 33.06 | 89.97 | 179 (193) |
| DR Congo | 0.382 | 84.51 (43.89 to 150.49) | 40.24 | 44.27 | 85 (99) |
| Ecuador | 0.64 | 123.09 (63.94 to 217.87) | 31.51 | 91.58 | 192 (195) |
| Egypt | 0.658 | 93.37 (48.28 to 168.4) | 31.23 | 62.14 | 159 (155) |
| El Salvador | 0.573 | 122.96 (63.99 to 217.11) | 33.38 | 89.58 | 178 (189) |
| Equatorial Guinea | 0.685 | 84.6 (43.77 to 151.22) | 31.3 | 53.3 | 132 (101) |
| Eritrea | 0.396 | 84.62 (43.68 to 151.77) | 40.24 | 44.39 | 87 (105) |
| Estonia | 0.835 | 87 (44.46 to 155.83) | 31.27 | 55.74 | 138 (133) |
| eSwatini | 0.577 | 84.01 (43.55 to 149.39) | 33.38 | 50.63 | 121 (91) |
| Ethiopia | 0.343 | 88.06 (45.23 to 158.29) | 40.34 | 47.72 | 104 (139) |
| Federated States of Micronesia | 0.58 | 40.66 (20.78 to 74.38) | 33.39 | 7.27 | 16 (16) |
| Fiji | 0.664 | 40.52 (20.47 to 74.46) | 31.26 | 9.26 | 30 (10) |
| Finland | 0.856 | 80.06 (41.03 to 143.9) | 31.3 | 48.76 | 108 (79) |
| France | 0.834 | 53.16 (26.92 to 95.68) | 31.29 | 21.87 | 48 (43) |
| Gabon | 0.656 | 84.65 (43.69 to 151.12) | 31.26 | 53.39 | 133 (107) |
| Georgia | 0.702 | 80.73 (41.35 to 146.78) | 31.28 | 49.45 | 111 (81.5) |
| Georgia | 0.841 | 80.73 (41.35 to 146.78) | 31.27 | 49.46 | 112 (81.5) |
| Germany | 0.898 | 58.97 (29.97 to 105.58) | 31.24 | 27.74 | 53 (48) |
| Ghana | 0.557 | 85.17 (44.29 to 152.31) | 33.4 | 51.76 | 127 (126) |
| Greece | 0.794 | 78.79 (40.5 to 144.24) | 31.28 | 47.51 | 103 (77) |
| Greenland | 0.761 | 62.32 (32.08 to 112.36) | 31.27 | 31.06 | 61 (56) |
| Grenada | 0.669 | 122.67 (63.77 to 217.15) | 31.32 | 91.35 | 185 (179) |
| Guam | 0.813 | 40.79 (20.73 to 74.4) | 31.26 | 9.53 | 37 (27) |
| Guatemala | 0.526 | 122.65 (63.88 to 216.19) | 33.39 | 89.26 | 174 (178) |
| Guinea | 0.325 | 85.02 (44.2 to 152.12) | 40.4 | 44.62 | 92 (122) |
| Guinea-Bissau | 0.355 | 84.94 (44.25 to 151.8) | 40.23 | 44.72 | 97 (120) |
| Guyana | 0.618 | 122.06 (63.61 to 216.09) | 32.53 | 89.53 | 177 (172) |
| Haiti | 0.432 | 122.34 (63.92 to 216.22) | 38.95 | 83.39 | 172 (173) |
| Honduras | 0.496 | 122.89 (63.78 to 217.2) | 33.73 | 89.16 | 173 (185) |
| Hungary | 0.791 | 77.94 (39.82 to 139.24) | 31.29 | 46.65 | 100 (75) |
| Iceland | 0.869 | 48.26 (24.27 to 87.58) | 31.27 | 17 | 44 (39) |
| India | 0.566 | 102.26 (53.1 to 184.31) | 33.39 | 68.87 | 166 (165) |
| Indonesia | 0.66 | 42.69 (21.62 to 76.51) | 31.29 | 11.4 | 41 (35) |
| Iran | 0.67 | 88.01 (44.53 to 158.39) | 31.28 | 56.73 | 142 (137) |
| Iraq | 0.671 | 93.1 (48.02 to 168.51) | 31.29 | 61.81 | 157 (152) |
| Ireland | 0.867 | 60.19 (30.52 to 109.23) | 31.29 | 28.9 | 57 (52) |
| Israel | 0.803 | 64.63 (32.81 to 116.78) | 31.24 | 33.39 | 62 (57) |
| Italy | 0.801 | 74.71 (38.33 to 134.16) | 31.25 | 43.46 | 80 (74) |
| Jamaica | 0.684 | 122.96 (64.16 to 217.35) | 31.24 | 91.72 | 196 (190) |
| Japan | 0.87 | 45.89 (23.33 to 83.33) | 31.27 | 14.62 | 43 (38) |
| Jordan | 0.731 | 93.09 (48.03 to 168.08) | 31.27 | 61.82 | 158 (151) |
| Kazakhstan | 0.723 | 80.7 (41.32 to 145.69) | 31.27 | 49.43 | 110 (80) |
| Kenya | 0.508 | 88.02 (45.12 to 158.02) | 33.42 | 54.59 | 134 (138) |
| Kiribati | 0.527 | 40.72 (20.81 to 74.69) | 33.42 | 7.31 | 18 (24) |
| Kuwait | 0.851 | 92.75 (48.03 to 168.06) | 31.26 | 61.49 | 155 (149) |
| Kyrgyzstan | 0.596 | 81.01 (41.34 to 146.37) | 33.11 | 47.9 | 105 (89) |
| Laos | 0.49 | 40.85 (20.91 to 74.8) | 33.8 | 7.04 | 14 (29) |
| Latvia | 0.82 | 87.02 (44.59 to 155.7) | 31.27 | 55.75 | 139 (134) |
| Lebanon | 0.708 | 93.84 (48.6 to 169.69) | 31.32 | 62.52 | 161 (160) |
| Lesotho | 0.507 | 83.97 (43.64 to 149.43) | 33.56 | 50.41 | 120 (90) |
| Liberia | 0.37 | 84.12 (43.73 to 149.83) | 40.23 | 43.89 | 83 (94) |
| Libya | 0.709 | 93.07 (48.21 to 168.06) | 31.28 | 61.79 | 156 (150) |
| Lithuania | 0.843 | 91.96 (47.19 to 165.83) | 31.27 | 60.69 | 153 (147) |
| Luxembourg | 0.895 | 60 (30.51 to 109.14) | 31.27 | 28.73 | 54 (49) |
| Madagascar | 0.396 | 84.91 (43.73 to 151.91) | 40.22 | 44.68 | 94 (117) |
| Malawi | 0.384 | 84.8 (43.98 to 151.79) | 40.23 | 44.57 | 88 (109) |
| Malaysia | 0.737 | 40.77 (20.94 to 75.49) | 31.33 | 9.44 | 36 (26) |
| Maldives | 0.562 | 40.6 (20.61 to 74.48) | 33.39 | 7.21 | 15 (13) |
| Mali | 0.263 | 84.97 (43.95 to 152.49) | 83.71 | 1.26 | 6 (121) |
| Malta | 0.801 | 60.1 (30.69 to 109.14) | 31.26 | 28.85 | 56 (51) |
| Marshall Islands | 0.544 | 40.43 (20.77 to 74.34) | 33.41 | 7.02 | 13 (7) |
| Mauritania | 0.496 | 85.2 (44.23 to 152.01) | 33.97 | 51.23 | 124 (128) |
| Mauritius | 0.705 | 40.66 (20.69 to 74.71) | 31.27 | 9.39 | 32 (17) |
| Mexico | 0.649 | 122.74 (64.16 to 220.76) | 31.25 | 91.49 | 190 (181) |
| Moldova | 0.696 | 86.99 (44.31 to 154.88) | 31.26 | 55.74 | 137 (132) |
| Monaco | 0.902 | 60.22 (30.58 to 108.75) | 31.24 | 28.98 | 59 (53) |
| Mongolia | 0.606 | 80.89 (41.37 to 147.09) | 32.82 | 48.07 | 107 (86) |
| Montenegro | 0.791 | 74.39 (38.05 to 134.22) | 31.25 | 43.14 | 77 (71) |
| Morocco | 0.548 | 93.56 (48.18 to 169.11) | 33.4 | 60.16 | 150 (157) |
| Mozambique | 0.307 | 84.46 (43.93 to 150.74) | 40.47 | 43.99 | 84 (98) |
| Myanmar | 0.521 | 40.92 (21 to 74.95) | 33.4 | 7.53 | 20 (32) |
| Namibia | 0.612 | 84.45 (43.85 to 151.24) | 32.57 | 51.88 | 129 (97) |
| Nauru | 0.618 | 40.75 (20.76 to 74.28) | 32.59 | 8.16 | 22 (25) |
| Nepal | 0.422 | 100.46 (51.93 to 181.31) | 40.23 | 60.23 | 151 (164) |
| Netherlands | 0.883 | 43.35 (22.02 to 78.99) | 31.3 | 12.06 | 42 (37) |
| New Zealand | 0.84 | 74.01 (37.79 to 133.74) | 31.28 | 42.73 | 68 (62) |
| Nicaragua | 0.517 | 122.9 (64.02 to 217.16) | 33.4 | 89.5 | 176 (186) |
| Niger | 0.162 | 85.11 (44.32 to 152.15) | 84.28 | 0.83 | 4 (125) |
| Nigeria | 0.515 | 88.18 (45.28 to 158.46) | 33.49 | 54.69 | 135 (140) |
| Niue | 0.711 | 40.58 (20.91 to 74.63) | 31.32 | 9.26 | 31 (11) |
| North Korea | 0.558 | 36.24 (18.5 to 65.15) | 33.39 | 2.85 | 8 (3) |
| North Macedonia | 0.744 | 74.33 (38.31 to 134.38) | 31.25 | 43.08 | 76 (70) |
| Northern Mariana Islands | 0.771 | 40.66 (20.55 to 74.69) | 31.26 | 9.41 | 33 (18) |
| Norway | 0.913 | 39.1 (19.98 to 71.24) | 31.29 | 7.81 | 21 (4) |
| Oman | 0.783 | 91.09 (46.6 to 165.1) | 31.32 | 59.77 | 146 (144) |
| Pakistan | 0.449 | 102.28 (52.44 to 185.09) | 35.74 | 66.54 | 165 (166) |
| Palau | 0.738 | 40.41 (20.58 to 73.88) | 31.27 | 9.14 | 28 (6) |
| Palestine | 0.588 | 93.14 (48.33 to 168.18) | 33.31 | 59.84 | 147 (153) |
| Panama | 0.686 | 122.93 (64.06 to 217.39) | 31.24 | 91.69 | 195 (187) |
| Papua New Guinea | 0.394 | 40.43 (20.54 to 73.6) | 40.24 | 0.19 | 2 (8) |
| Paraguay | 0.638 | 125.91 (65.11 to 223.26) | 31.47 | 94.44 | 204 (204) |
| Peru | 0.648 | 123.26 (64.08 to 218.57) | 31.29 | 91.96 | 200 (201) |
| Philippines | 0.623 | 42.73 (21.68 to 76.63) | 32.19 | 10.54 | 40 (36) |
| Poland | 0.802 | 105.34 (53.64 to 185.83) | 31.32 | 74.02 | 170 (170) |
| Portugal | 0.743 | 65.16 (33.15 to 115.74) | 31.28 | 33.88 | 63 (58) |
| Puerto Rico | 0.814 | 122.96 (63.89 to 217.75) | 31.28 | 91.67 | 194 (188) |
| Qatar | 0.83 | 88.75 (45.71 to 160.37) | 31.24 | 57.52 | 143 (141) |
| Romania | 0.76 | 74.51 (38.09 to 135) | 31.26 | 43.25 | 79 (73) |
| Russia | 0.805 | 86.19 (43.95 to 157.49) | 31.29 | 54.9 | 136 (131) |
| Rwanda | 0.429 | 84.81 (43.99 to 152.08) | 40.23 | 44.58 | 90 (110) |
| Saint Kitts and Nevis | 0.746 | 122.76 (63.87 to 217.32) | 31.28 | 91.48 | 189 (182) |
| Saint Lucia | 0.67 | 122.59 (63.82 to 216.58) | 31.26 | 91.33 | 184 (175) |
| Saint Vincent and the Grenadines | 0.627 | 122.64 (63.87 to 217.49) | 32.3 | 90.34 | 182 (176) |
| Samoa | 0.641 | 40.68 (20.66 to 74.96) | 31.56 | 9.12 | 27 (19) |
| San Marino | 0.884 | 60.33 (30.73 to 109.56) | 31.28 | 29.04 | 60 (55) |
| Saudi Arabia | 0.805 | 91.6 (47.6 to 164.95) | 31.29 | 60.31 | 152 (146) |
| Senegal | 0.389 | 84.91 (44.04 to 151.92) | 40.22 | 44.69 | 95 (118) |
| Serbia | 0.767 | 74.33 (38.08 to 134.55) | 31.27 | 43.06 | 74 (69) |
| Seychelles | 0.724 | 40.7 (20.85 to 74.85) | 31.28 | 9.43 | 34 (21) |
| Sierra Leone | 0.347 | 84.85 (43.9 to 151.72) | 40.28 | 44.57 | 89 (114) |
| Singapore | 0.861 | 56.94 (29.19 to 101.88) | 31.26 | 25.68 | 52 (47) |
| Slovakia | 0.812 | 74.33 (38.13 to 134.35) | 31.26 | 43.07 | 75 (68) |
| Slovenia | 0.84 | 74.25 (37.97 to 133.8) | 31.26 | 42.99 | 72 (66) |
| Solomon Islands | 0.407 | 40.62 (20.59 to 74.24) | 40.24 | 0.38 | 3 (14) |
| Somalia | 0.081 | 84.62 (43.81 to 150.69) | 84.48 | 0.14 | 1 (104) |
| South Africa | 0.678 | 87.46 (45.04 to 156.38) | 31.3 | 56.16 | 141 (136) |
| South Korea | 0.878 | 52.51 (26.67 to 94.45) | 31.28 | 21.22 | 47 (42) |
| South Sudan | 0.363 | 84.09 (43.5 to 149.57) | 40.23 | 43.86 | 82 (92) |
| Spain | 0.767 | 54.06 (27.5 to 97.4) | 31.29 | 22.77 | 49 (44) |
| Sri Lanka | 0.69 | 40.85 (20.85 to 74.64) | 31.25 | 9.6 | 38 (30) |
| Sudan | 0.515 | 93.45 (48.38 to 169.37) | 33.44 | 60.01 | 149 (156) |
| Suriname | 0.636 | 122.52 (63.63 to 216.27) | 31.72 | 90.8 | 183 (174) |
| Sweden | 0.872 | 55 (28.02 to 99.92) | 31.25 | 23.74 | 50 (45) |
| Switzerland | 0.929 | 35.84 (18.35 to 65.01) | 31.32 | 4.52 | 10 (2) |
| Syria | 0.619 | 93.73 (48.28 to 169.47) | 32.54 | 61.19 | 154 (159) |
| Taiwan (province of China) | 0.868 | 40.02 (20.29 to 71.77) | 31.24 | 8.78 | 25 (5) |
| Tajikistan | 0.539 | 80.75 (41.31 to 145.44) | 33.4 | 47.35 | 102 (83) |
| Tanzania | 0.423 | 84.85 (43.98 to 151.18) | 40.23 | 44.62 | 93 (115) |
| Thailand | 0.687 | 41.01 (21.03 to 75.29) | 31.25 | 9.76 | 39 (33) |
| The Bahamas | 0.796 | 123.09 (64.04 to 217.48) | 31.27 | 91.83 | 197 (196) |
| The Gambia | 0.399 | 84.84 (43.81 to 151.64) | 40.24 | 44.59 | 91 (113) |
| Timor-Leste | 0.514 | 40.71 (20.8 to 74.91) | 33.43 | 7.28 | 17 (22) |
| Togo | 0.417 | 85.21 (44.05 to 151.9) | 40.24 | 44.97 | 99 (130) |
| Tokelau | 0.626 | 40.68 (20.68 to 74.66) | 32.2 | 8.49 | 23 (20) |
| Tonga | 0.636 | 40.82 (20.78 to 74.4) | 31.72 | 9.11 | 26 (28) |
| Trinidad and Tobago | 0.757 | 122.68 (63.84 to 217.85) | 31.26 | 91.42 | 188 (180) |
| Tunisia | 0.672 | 93.89 (48.29 to 169.17) | 31.26 | 62.63 | 162 (161) |
| Turkey | 0.748 | 109.67 (57.2 to 198.02) | 31.28 | 78.38 | 171 (171) |
| Turkmenistan | 0.67 | 80.93 (41.25 to 146.86) | 31.26 | 49.66 | 114 (88) |
| Tuvalu | 0.589 | 40.65 (20.79 to 73.97) | 33.32 | 7.33 | 19 (15) |
| Uganda | 0.404 | 84.93 (44.11 to 151.64) | 40.23 | 44.7 | 96 (119) |
| UK | 0.847 | 79.31 (40.78 to 142.97) | 31.29 | 48.01 | 106 (78) |
| Ukraine | 0.736 | 89.79 (46.08 to 163.81) | 31.28 | 58.51 | 145 (143) |
| United Arab Emirates | 0.88 | 89.59 (46.3 to 161.86) | 31.3 | 58.29 | 144 (142) |
| Uruguay | 0.697 | 105.19 (54.32 to 187.87) | 31.32 | 73.87 | 168 (169) |
| USA | 0.859 | 73.5 (37.17 to 131.47) | 31.28 | 42.22 | 67 (61) |
| Uzbekistan | 0.631 | 80.83 (41.4 to 145.85) | 31.74 | 49.09 | 109 (84) |
| Vanuatu | 0.485 | 40.59 (20.78 to 73.86) | 33.93 | 6.65 | 12 (12) |
| Venezuela | 0.607 | 123.03 (63.74 to 217.04) | 32.77 | 90.26 | 181 (194) |
| Vietnam | 0.617 | 41.08 (21.03 to 75.32) | 32.34 | 8.74 | 24 (34) |
| Virgin Islands | 0.799 | 123.2 (63.97 to 218.49) | 31.29 | 91.91 | 199 (198) |
| Yemen | 0.412 | 93.27 (47.96 to 169.35) | 40.24 | 53.02 | 131 (154) |
| Zambia | 0.505 | 84.55 (43.64 to 150.77) | 33.48 | 51.07 | 123 (100) |
| Zimbabwe | 0.476 | 84.63 (43.91 to 150.81) | 34.6 | 50.03 | 117 (106) |

SDI: Socio-demographic index; UI: uncertainty interval; YLDs: Years Lived with Disability.
